# Covalency of M–N Bonds in Isomorphous Lanthanide and Actinide 5‑(2-Pyridyl)‑1*H*‑tetrazolate Complexes

**DOI:** 10.1021/jacsau.5c01374

**Published:** 2026-02-27

**Authors:** Zhuanling Bai, Madeline C. Martelles, Qiang Gao, Nicholas B. Beck, Jacob P. Brannon, Joseph M. Sperling, Thomas E. Albrecht

**Affiliations:** Department of Chemistry and Nuclear Science and Engineering Center, 3557 Colorado School of Mines, Golden, Colorado 80401, United States

**Keywords:** Chemical Bonding in Lanthanides and Actinides, Chemistry of Transuranium Elements, Coordination Complexes of Plutonium, Americium, and Curium, Perioidic Trends in the f-block, Soft-Donor Ligands with Actinides

## Abstract

Experimental and computational analyses of [M­(pdtz)_3_(H_2_O)_3_]·3.5H_2_O (M^3+^ = Pu^3+^–Cm^3+^, La^3+^–Nd^3+^, and Sm^3+^–Ho^3+^, pdtz^–^ = 5-(2-pyridyl)-1*H*-tetrazolate) were conducted to understand potential differences in bonding between lanthanide and actinide complexes with a N-donor ligand. Structural analyses show that the An–N bond distances in the Pu^3+^, Am^3+^, and Cm^3+^ complexes are within error of one another. Whereas in the lanthanide series, there is a nearly linear decrease in the Ln–N bond lengths from La^3+^ to Ho^3+^ (excluding Pm^3+^). The An–N bond lengths are ∼0.015 Å shorter than their similarly-sized lanthanide analogs, in agreement with computational results that suggest greater covalent character in these bonds versus those with lanthanides. QTAIM analysis indicates that the An–N orbital mixing remains essentially unchanged from Pu^3+^ to Cm^3+^, consistent with the nearly identical An–N bond lengths. However, upon deconvolution of the NLMOs into orbital compositions, the metal orbital contributions to An–N bonding decreases slightly overall wherein the 6d involvement remains constant, 7s involvement slightly increases, and 5f participation decreases. The molecular orbital energy diagram indicates that energy degeneracy between the 5f metal and 2p ligand orbitals increases from Pu^3+^ to Cm^3+^ and counteracts the contraction of the 5f orbtials. Together with prior reports of decreasing energy degeneracy between 5f and 3p orbitals from Np^3+^ to Cf^3+^, these observations provide guidance on understanding how chemical bonding evolves in the actinide series.

## Graphical abstract

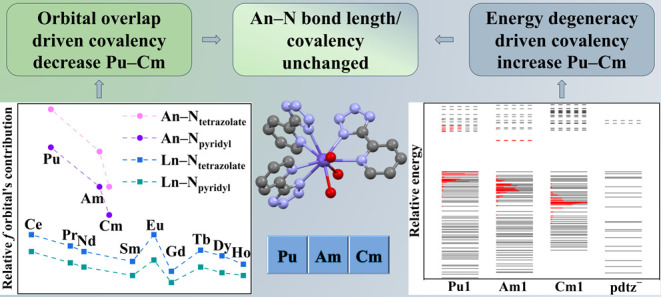

## Introduction

Understanding the nature of chemical bonding in f-element complexes is currently a focus of intense research, driven by both academic interests in f-orbital bonding and industrial demands.^1^ Although definitive support for this interpretation has yet to be established, increased orbital mixing is widely regarded as a key factor underlying the preference of soft-donor ligands for actinides over lanthanides, and thus plays a critical role in controlling trivalent An/Ln separations.^2–4^ In particular, variations in covalent bonding contributions are thought to influence the efficiency of some solvent extraction processes used to separate Am^3+^ and Cm^3+^ from lanthanides.^5,6^ Such separations are essential for nuclear waste storage and for partitioning and transmuting transuranium elements.^7–10^ Neptunium, along with uranium and plutonium, can be extracted from dissolved nuclear fuel in processes like Plutonium Uranium Reduction Extraction (PUREX)^11^ or the COEX^12^ process.^13^ However, the separation of Am^3+^ and Cm^3+^ from their lanthanide counterparts, Ln^3+^, is challenging owing to similarities in the oxidation state and ionic radii.^5^ Hence, more structural, spectroscopic, and quantum mechanical studies of lanthanide and actinide compounds with soft-donor ligands (for example, C, N, S, Cl, etc.) are needed to advance the separations of actinides and lanthanides.

Since Seaborg’s proposal that the separation of actinides from lanthanides is caused by 5f orbitals participating in covalent bonding,^14^ a broad range of compounds have been investigated to understand the degree of 5f-orbital hybridization with different donor atoms.^1^ For example, in AnCp_3_ (An = Th–Cm; Cp^–^ = η^5^-C_5_H_5_), analysis of the molecular orbitals reveals 5f contributions to forming An-L bonds and spin densities increase from Th to Am. However, atoms-in-molecules analysis shows the covalency in An–Cp is arising mainly from the energy degeneracy of metal and ligand orbitals rather than from significant orbital overlap.^15^ Two principal components of orbital mixing in actinide compounds are recognized: one governed by orbital overlap and the other by energy degeneracy.^6,16^ Studies of actinide–dipicolinate complexes reveal that heavier actinides (Bk, Cf) exhibit greater covalency than earlier ones.^17^ Electronic structure calculations conducted by Kelley and co-workers indicate that although the An–L bonding remains largely ionic, the reduced 5f orbital energies toward the end of the series enhance orbital mixing through energy degeneracy with ligand 2p orbitals. This demonstrates that covalency in heavy actinides can be enhanced by energy degeneracy when only a small degree of orbital overlap is present.^18^ Orbital overlap is a prerequisite for orbital mixing and orbital energy degeneracy alone cannot lead to covalent bonding. Likewise, in [An­(mnt)_4_]^5–^ (An^3+^ = Np^3+^, Pu^3+^, Am^3+^, Cm^3+^, and Cf^3+^, mnt^2–^ = maleonitrile-1,2-dithiolate), the 5f orbitals are energetically degenerate with sulfur 3p orbitals from neptunium to americium, leading to significant Np/Pu–S bond contraction and stabilization and observation of a nonlinear bond length trend across the actinide series. In the curium and californium complexes, the 5f orbitals lie lower in energy and instead become degenerate with the delocalized π orbitals of the mnt^2–^ ligand.^19^ Here, we pose a question: how does the energy degeneracy between the N-containing ligand 2p orbitals and actinide 5f orbitals evolve across the actinide series?

Although the aforementioned studies have contributed toward a fundamental understanding of An–L bonding, from a disposal and waste management perspective the CHON principle is typically followed,^20^ and heterocyclic N-donor ligands are often preferred. Examples of such ligands for complexing actinides include azides,^21^ phenanthroline derivatives, 2,4,6-*tris*(2-pyridyl)-*s*-triazine, 2,6-*bis*(5,6-dialkyl-1,2,4-triazin-3-yl)­pyridine, and 6,6′-*bis*(5,6-dialkyl-1,2,4-triazin-3-yl)-2,2′-bipyridine derivatives.^2^ Liquid–liquid extraction is the primary method for performing the challenging separation of An^3+^/Ln^3+^, as exemplified by the SANEX process that uses hydrophobic N-donor ligands for selective separation.^22,23^ The SANEX process using *i*Pr-BTP (2,6-*bis*(5,6-iso-propyl-1,2,4-triazin-3-yl)-pyridine) was tested on An­(III)/Ln­(III) solutions from DIAMEX, but solvent radiolysis destroyed *i*Pr-BTP, prompting the development of more robust BTPs, and alternative BTP-derived ligands were selected for future SANEX flowsheets.^24^ Similar to BTP ligands, the tetrazolate-based ligand, 6-(tetrazol-5-yl)-2,2′-bipyridine (HN_4_bipy), forms 1:3 metal–ligand complexes with stability constants corresponding to a theoretical separation factor of SF_Cm(III)/Eu(III)_ ≈500.^25^ This emphasizes that An/Ln–N bond covalency should be enhanced in the design of more selective extractants.^26–30^ For example, [Am­(EtBTP)_3_]^3+^ exhibits shorter M–N bond lengths than those observed for [Nd­(EtBTP)_3_]^3+^ by 0.0158(18) Å due to back-donation that is not observed in the Nd complex.^31^

Here, the 5-(2-pyridyl)-1*H*-tetrazole (**Hpdtz**) ligand was employeed to provide a range of chemically-distinct N-donor sites that should create a wide variety of coordination modes that will aid in comparisons of transuranium coordination chemistry with other nitrogen-donor complexes that have been previously reported.^32–37^ This work reports on the syntheses, crystal structures, spectroscopy, and quantum mechanical simulations of Pu­(III), Am­(III), and Cm­(III) coordination complexes along with an isostructural series of lanthanide-pdtz complexes [M­(pdtz)_3_(H_2_O)_3_]·3.5H_2_O (M^3+^ = Pu^3+^–Cm^3+^, La^3+^–Nd^3+^, and Sm^3+^–Ho^3+^, pdtz^–^ = 5-(2-pyridyl)-1*H*-tetrazolate, Scheme 1) to investigate the discrete differences in chemical bonding between the lanthanide and actinide complexes.

**Scheme 1:**
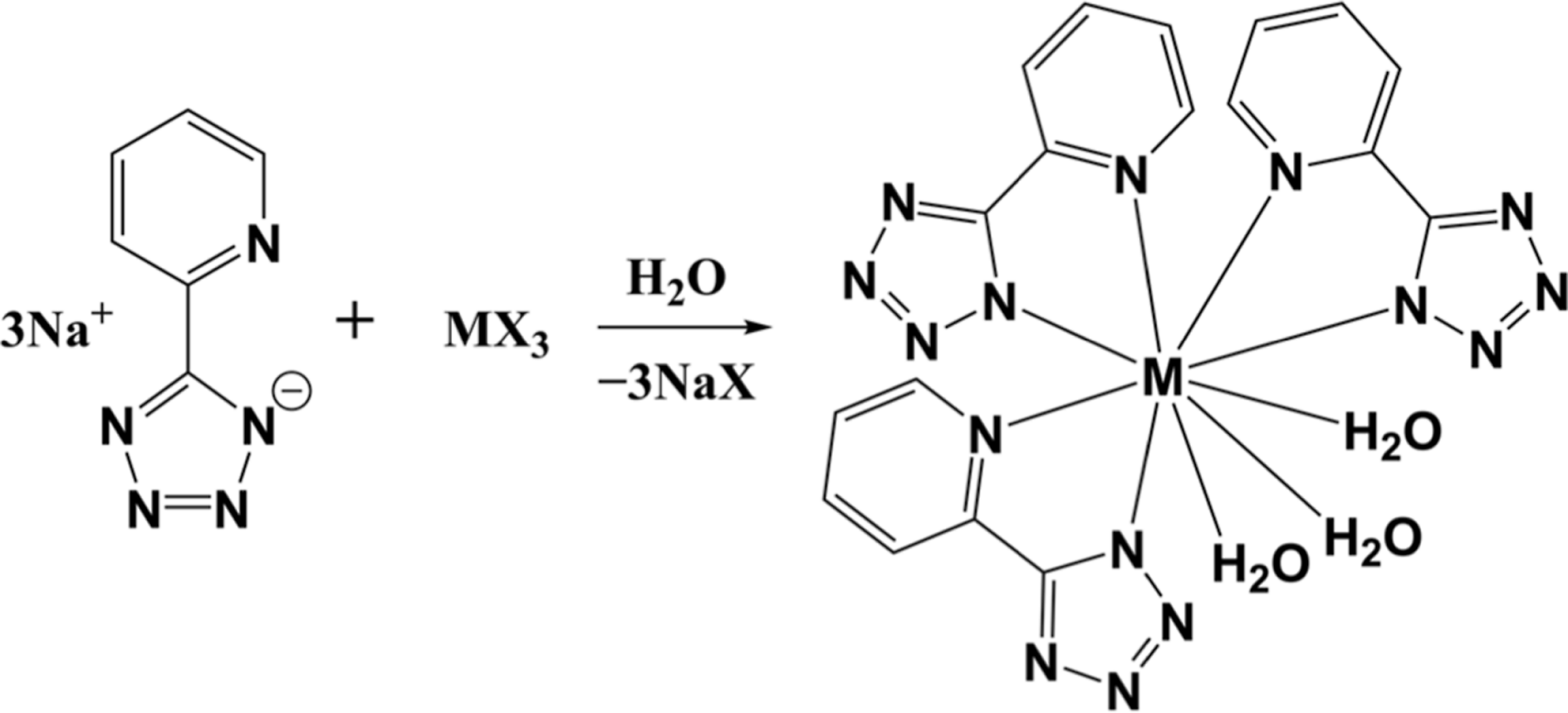
Synthesis of [M­(pdtz)_3_(H_2_O)_3_]·3.5H_2_O (M^3+^ = Pu^3+^–Cm^3+^, La^3+^–Nd^3+^, and Sm^3+^–Ho^3+^, pdtz^–^ = 5-(2-Pyridyl)-1*H*-tetrazolate)

## Results and Discussion

[M­(pdtz)_3_(H_2_O)_3_]·3.5H_2_O (pdtz^–^ = 5-(2-pyridyl)-1*H*-tetrazolate and M^3+^ = Pu^3+^–Cm^3+^, La^3+^–Nd^3+^, and Sm^3+^–Ho^3+^) were synthesized via salt metathesis reactions of MX_3_·*n*H_2_O (X^–^ = Cl^–^ or Br^–^) and Na­(pdtz) in aqueous media. Attempts to prepare the corresponding U^3+^ and Np^3+^ complexes were unsuccessful due to redox instability under the reaction conditions. As shown in Figure 1, these f-element–pdtz complexes crystallize in the monoclinic space group *P*2_1_/*n*, and are isostructural with the previously reported [Ln­(pdtz)_3_(H_2_O)_3_]·3.5H_2_O complexes (Ln = La^33^ and Gd^32^). In addition, the reaction of excess hydrated LnCl_3_ (Ln = La^3+^, Gd^3+^, Ho^3+^, Yb^3+^, Y^3+^) with sodium 5-(2-pyridyl)­tetrazolate in water afforded [Ln­(pytz)_2_(H_2_O)_5_]Cl (Ln = La^3+^, Gd^3+^, Ho^3+^), [Ho­(pdtz)_3_(H_2_O)_4_]·4H_2_O, and [Ln_2_(pdtz)_4_(μ–OH)_2_(H_2_O)_4_]·2H_2_O (Ln = Yb^3+^ and Y^3+^).^38^

**Figure 1:**
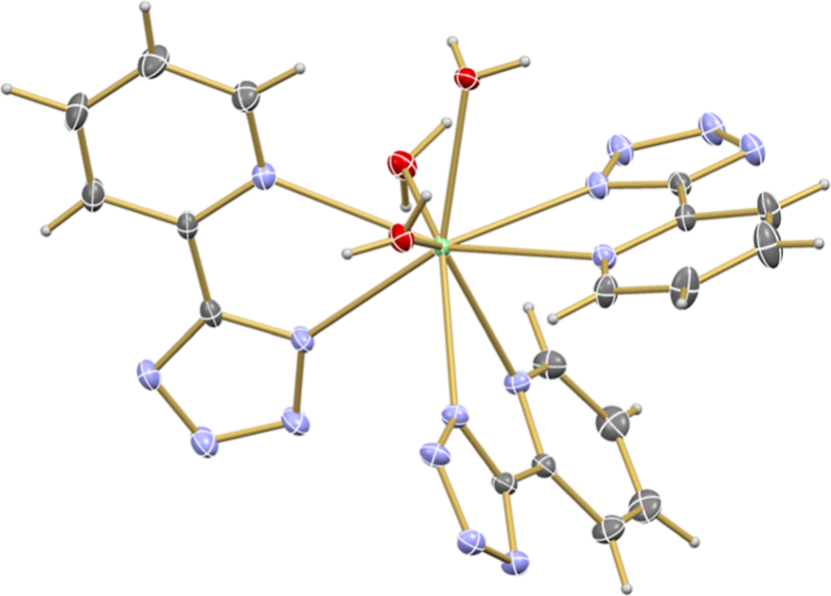
Molecular structure of [M­(pdtz)_3_(H_2_O)_3_]·3.5H_2_O, with **Cm1** as an example, drawn with displacement ellipsoids at the 50% probability level at 100 K (M^3+^ = Pu^3+^–Cm^3+^, La^3+^–Nd^3+^, and Sm^3+^–Ho^3+^, light green: N, light blue: O, red: C, light gray: H). Noncoordinated water molecules are omitted for clarity.

In the [M­(pdtz)_3_(H_2_O)_3_]·3.5H_2_O structures, the M^3+^ centers adopt a nine-coordinate geometry and are coordinated by three water molecules and three bidentate pdtz^–^ ligands. Each pdtz^–^ ligand binds through one tetrazolate nitrogen atom and one pyridyl nitrogen atom, resulting in a geometry that is closest to a distorted capped square antiprism, as determined by SHAPE calculations (detailed in Section S7).^39,40^ Additionally, there are 3.5 noncoordinating water molecules that participate in an extensive hydrogen-bonding network with the pdtz^–^ ligands and coordinated water molecules (discussed in Section S6). Further characterization and descriptions of the PXRD data, solid-state UV–vis–NIR absorption spectroscopy, and Raman and ^1^H NMR (in D_2_O) spectra of **Ln1** are provided in Section S2.

Figure 2 exhibits the average bond length (Å) of M–O, M–N_tetrazolate_, and M–N_pyridyl_ with trend lines plotted against the six-coordinate ionic radii of the corresponding trivalent metals with detailed individual bond lengths provided in Supporting Information, Sections S2–S6. ^VI^M­(III) stands for six-coordinated trivalent metal ions. Six-coordinate radii were used because no nine-coordinate Cm­(III) ionic radius data have been reported. Analysis of the average bond lengths reveals a nearly linear decrease in the Ln–OH_2_ and Ln–N bond lengths across the lanthanide series from lanthanum to holmium (excluding promethium), in accordance with the well-established lanthanide contraction. Additionally, all M–N_tetrazolate_ bonds are significantly shorter than M–N_pyridyl_ bonds, with an average difference of approximately 0.1 Å. Notably, the An–N bond lengths in **Pu1**, **Am1**, and **Cm1** are statistically indistinguishable within experimental error, as are the An–OH_2_ distances. This is unusual because in many other An^3+^ compounds a linear decrease in the average bond length with respect to ionic radius is observed as exemplified by [An­(H_2_O)_9_]­(CF_3_SO_3_)_3_ (An^3+^ = U^3+^–Cf^3+^ excl. Bk^3+^)^41^ and actinide mellitates (An^3+^ = Np^3+^–Cf^3+^).^42–47^

**Figure 2:**
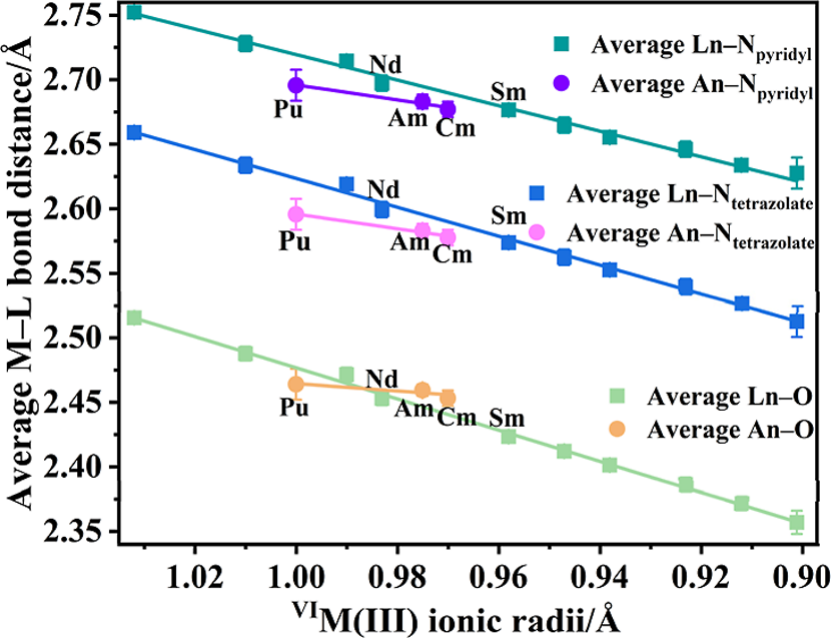
Average bond length (Å) at 100 K of M–O, M–N_tetrazolate_, and M–N_pyridyl_ of isostructural f-element-pdtz complexes, [M­(pdtz)_3_(H_2_O)_3_]·3.5H_2_O, with trend lines versus the corresponding trivalent metal six-coordinated ionic radii. ^VI^M­(III) stands for six-coordinated trivalent metal ions. The reported errors correspond to three times the standard deviation of the measured values.

The Pu–OH_2_ bond lengths (2.427(4) – 2.484(4) Å) for **Pu1** are shorter than Ce–OH_2_ in **Ce1** (2.4563(18) Å – 2.5089(18) Å) and comparable to Pr–OH_2_ in **Pr1** (2.4392(15) Å – 2.4915(15) Å) and Nd–OH_2_ in **Nd1** (2.4253(12) – 2.4722(13) Å). In contrast, the Pu–N bond lengths in **Pu1** (2.585(3) – 2.706(4) Å) are shorter than Ce–N in **Ce1** (2.620(2) Å – 2.738(2) Å) and are comparable within experimental error to the Pr–N (2.6079(17) Å – 2.7242(18) Å) in **Pr1** as well as Nd–N bond lengths [2.5868(14) – 2.7095(14) Å] in **Nd1**. These Pu–OH_2_ and Pu–N bonds are consistent with those of recently reported ^IX^Pu­(III)-tetrazolate complexes, such as [Pu­(pmtz)_3_(H_2_O)_3_]·8H_2_O,^48^ [(Pu­(pmtz)_2_(H_2_O)_3_)_2_(μ-pmtz)]_2_(pmtz)_2_·14H_2_O^48^ (pmtz^–^ = 5-(pyrimidyl)­tetrazolate), [M­(Hdtb)­(H_2_O)_8_]­(dtb)·11H_2_O (dtb^2–^ = 1,3-di­(tetrazolate-5-yl)­benzene),^49^ and Na_2_[Pu­(Hdtp)­(dtp)_2_(H_2_O)_4_]·9H_2_O (H_2_dtp = 2,3-di-1*H*-tetrazol-5-ylpyrazine).^50^

The An–OH_2_ bond lengths (2.4282(15) – 2.4822(16) Å) for **Am1** and (2.4235(19) – 2.471(2) Å) for **Cm1** are within the experimental error of those in **Nd1** (2.4253(12) – 2.4722(13) Å). In fact, the bond length ranges for all three actinide complexes fall within the error range of the Nd–OH_2_ bonds in **Nd1**. However, the Am–N bond lengths (2.5711(18) – 2.6958(18) Å) for **Am1** and the Cm–N bond lengths (2.569(2) – 2.693(2) Å) for **Cm1** are statistically shorter than Nd–N bond lengths in **Nd1** and are also within error of the Sm–N bond lengths ranging from 2.5604(13) to 2.6940(13) Å, shown in Figure 2. The average Am–N_pyridyl_ and Cm–N_pyridyl_ bonds are shorter than average Nd–N_pyridyl_ (Δ = 0.0142(18) and 0.020(2) Å, respectively). The average Am–N_tetrazolate_ and Cm–N_tetrazolate_ bonds are shorter than average Nd–N_tetrazolate_ (Δ = 0.0165(18) and 0.022(2) Å, respectively). Furthermore, these An–OH_2_ and An–N bonds fall within the range of recently reported corresponding An^3+^ complexes, such as [Am­(pda)­(NO_3_)­(H_2_O)_2_]·H_2_O^51^ (H_2_pda = 1,10-phenanthroline-2,9-dicarboxylic acid), Am­(tpyNO_2_)­(NO_3_)_3_(H_2_O)·THF^52^ (tpyNO_2_ = 4′-nitrophenyl terpyridyl), [Cm­(Hdpa)­(H_2_dpa)­(H_2_O)_2_Cl]­Cl·2H_2_O (H_2_dpa = 2,6-pyridinedicarboxylic acid),^53^ and [(Cm­(pmtz)_2_(H_2_O)_3_)_2_(μ-pmtz)]_2_(pmtz)_2_·14H_2_O.^54^

Analysis of electron density has been widely used to quantify f-element covalency.^6^ To gain deeper insight into the chemical bonding in [M­(pdtz)_3_(H_2_O)_3_]·3.5H_2_O, Bader’s Quantum Theory of Atoms in Molecules (QTAIM) was applied to analyze electron density, energy densities (potential, kinetic, and total), and delocalization indices at the bond critical points, as shown in Figure 3. Fully detailed metrics can be found in Section S9 in the Supporting Information. Larger values of electron density (ρ­(r)) tend to, in part, indicate greater covalent contributions. More negative values of the bond strength parameter (*H*(r)/ρ­(r)) correspond to more covalent character. |*V*(r)|/*G*(r) is the ratio of the potential energy density to the kinetic energy density at a bond critical point, and it serves as a measure of bond covalency, with values greater than 1 indicating a shared (covalent) interaction and values less than 1 indicating a closed-shell (ionic) interaction. The delocalization index (δ­(r)) quantifies the number of electrons shared between two atoms in a molecule, derived from the second order reduced density matrix. Formally, it is the covariance of the electron populations of the two atoms and serves as a measure of both covalency and bond order.^55^

**Figure 3:**
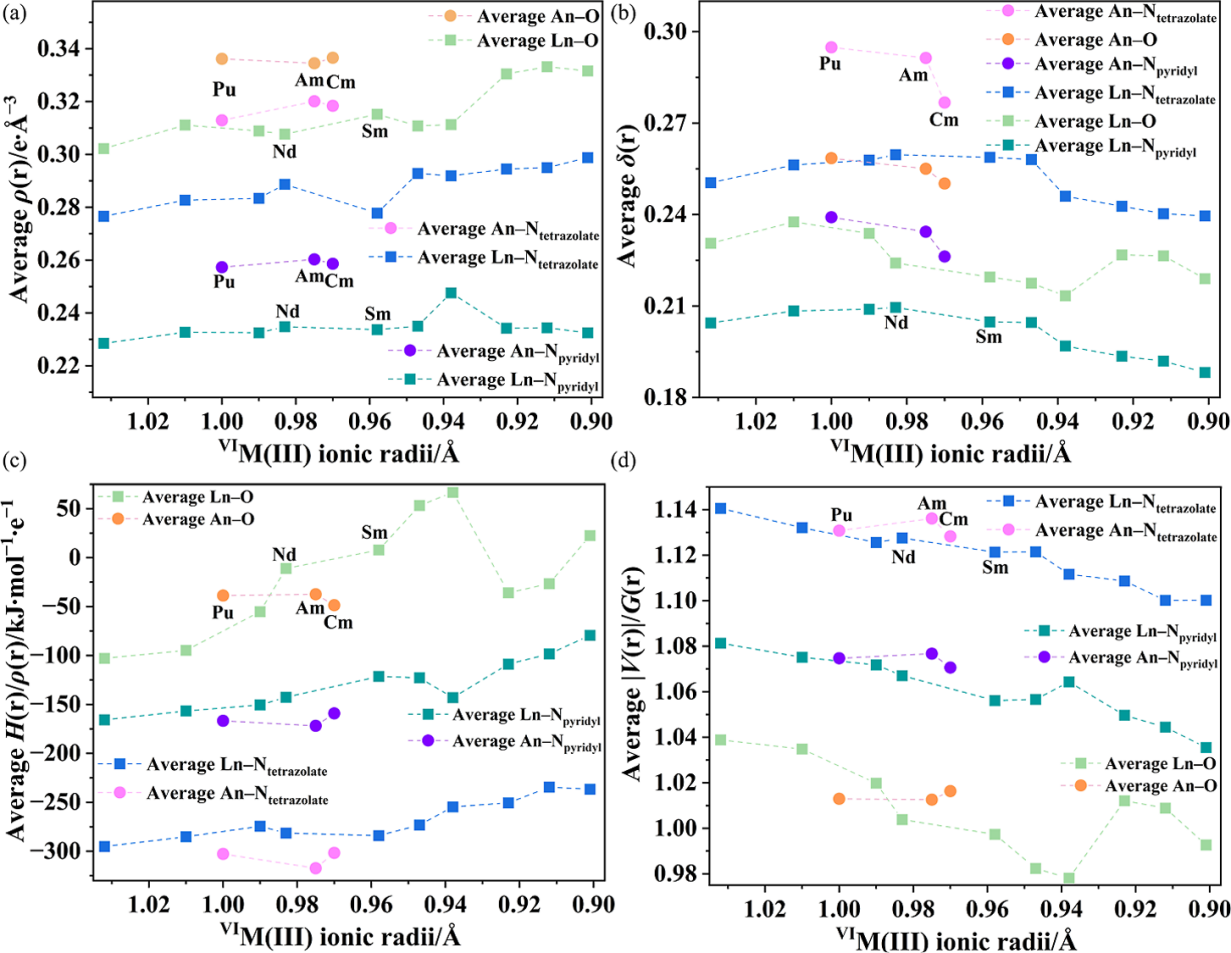
QTAIM metrics of M–OH_2_ and M–N_tetrazolate_ and M–N_pyridyl_ bonds in [M­(pdtz)_3_(H_2_O)_3_]·3.5H_2_O, including (a) the electron density ρ­(r) in e·Å^–3^, (b) delocalization index, δ­(r), (c) bond strength parameter *H*(r)/ρ­(r), and (d) |*V*(r)|/*G*(r) at the bond critical points (BCPs).^55^

In general, larger electron density ρ­(r), more negative potential (*V*) and total (*H*) energy densities, and a higher bond strength parameter *H*(r)/ρ­(r) and |*V*(r)|/*G*(r) at the BCPs of An–L bonds indicate stronger bonding compared to Ln–L bonds. Using the same comparison, M–N_tetrazolate_ bonds are stronger than M–N_pyridyl_ bonds. Across the Ln–L series from **La1** to **Ho1**, the metrics remain largely unchanged, showing only minor variations without a clear trend. In the An–L series from **Pu1** to **Cm1**, the electron density parameters are also largely constant. However, the delocalization index δ­(r) for the An–L bonds exhibits a downward trend, indicating progressively fewer electrons are shared between An^3+^ and the ligands from **Pu1** to **Cm1**.

To distinguish between stabilization arising from ionic and covalent effects, the interacting quantum atom (IQA) approach was employed. *V*_XC_, the exchange-correlation part of the two-electron interaction energy, and *V*_XC_/*E*_int_, the exchange-correlation contributions to the total interaction energy between the metal center and ligand in IQA theory, have recently been introduced as a covalency metric for the f-block.^57–59^ In this method, the interatomic energy (*E*_int_) between two atoms is decomposed into a classic electrostatic term (*V*_cl_), including nuclear–nuclear repulsion, electron–electron Coulombic repulsion, and electron–nuclear attraction energies, and an exchange-correlation term (*V*_XC_) serving as a reliable descriptor of the covalent contribution to the interatomic energy. More definitions can be found in Section S11 in the SI. The significantly more negative number of classical electrostatic interaction, *V*_cl_, than exchange-correlation part of two-electron interaction, *V*_XC_ means the electrostatic interactions dominate the interactions, but the covalency contributions are also not negligible at more than 8% of the total energy. As shown in Figure 4a,b, the more negative value of exchange-correlation and larger exchange-correlation contributions to the total interaction energy of M–N_tetrazolate_ bonds indicate greater covalency compared to M–N_pyridyl_ bonds.

**Figure 4:**
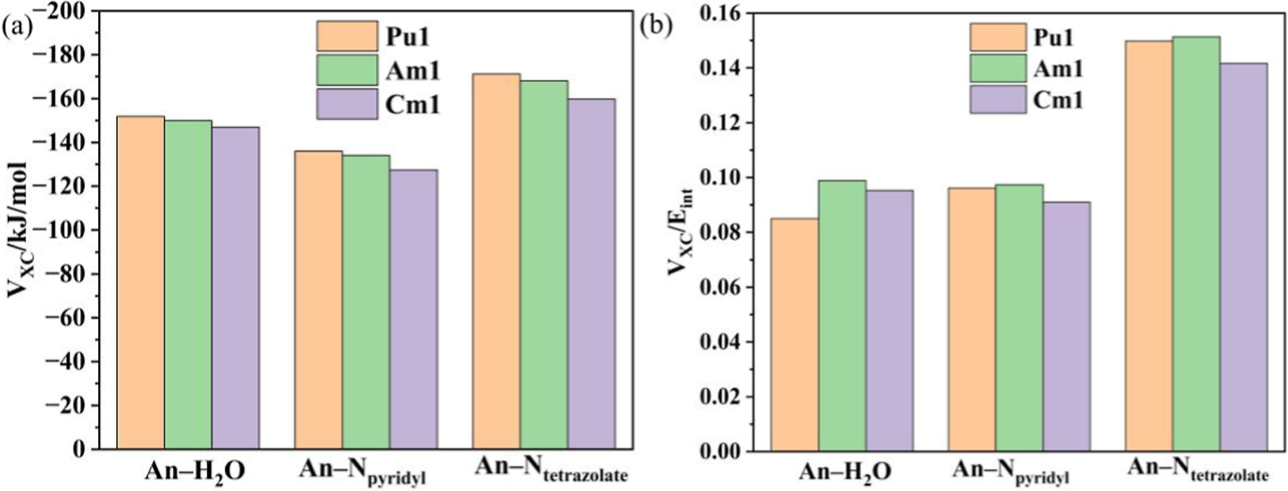
Energy decomposition on the basis of IQA definition including (a) *V*_XC_ and (b) *V*_XC_/*E*_int_.^56^

Consistent with the largely unchanged bonding density parameters of M–L bonds from **Pu1** to **Cm1**, the relatively constant *V*_XC_ and *V*_XC_/*E*_int_ values indicate that the degree of covalency remains relatively constant across the series from **Pu1** to **Cm1**. While *V*_XC_ and *V*_XC_/*E*_int_ values decrease slightly from those of **Am1** to those of **Cm1**, the differences are minor and lie within the computational uncertainty. Although fewer electrons are shared between the two atoms from **Pu1** to **Cm1** as discussed previously in delocalization index δ­(r) results, it is likely that increased energy degeneracy compensates for this reduction in electron sharing, thereby maintaining the overall covalent character.

To further elucidate spatial orbital overlap and energy degeneracy-driven covalency of M–L bonds in [M­(pdtz)_3_(H_2_O)_3_]·3.5H_2_O, Natural Localized Molecular Orbitals (NLMOs) analysis (Figure 5) was performed. The metal contribution to NLMOs corresponding to the Ln–OH_2_, Ln–N_pyridyl_, and Ln–N_tetrazolate_ bonds remains relatively consistent with only a slight increase across the lanthanide series, ranging from 3.3% to 3.5%, 4.6% to 4.8%, and 5.3% to 6.1%, respectively. In contrast, in the actinide series, the metal contribution to NLMOs in An–OH_2_, An–N_pyridyl_, and An–N_tetrazolate_ gradually decreases from **Pu1** to **Cm1**, falling from 4.6% to 4.3%, 6.8% to 5.7%, and 8.2% to 7.3%, respectively. This **Pu1**–**Cm1** trend aligns with the previously discussed decrease in the exchange-correlation component of the two-electron interaction and the reduced delocalization index δ­(r) values.

**Figure 5:**
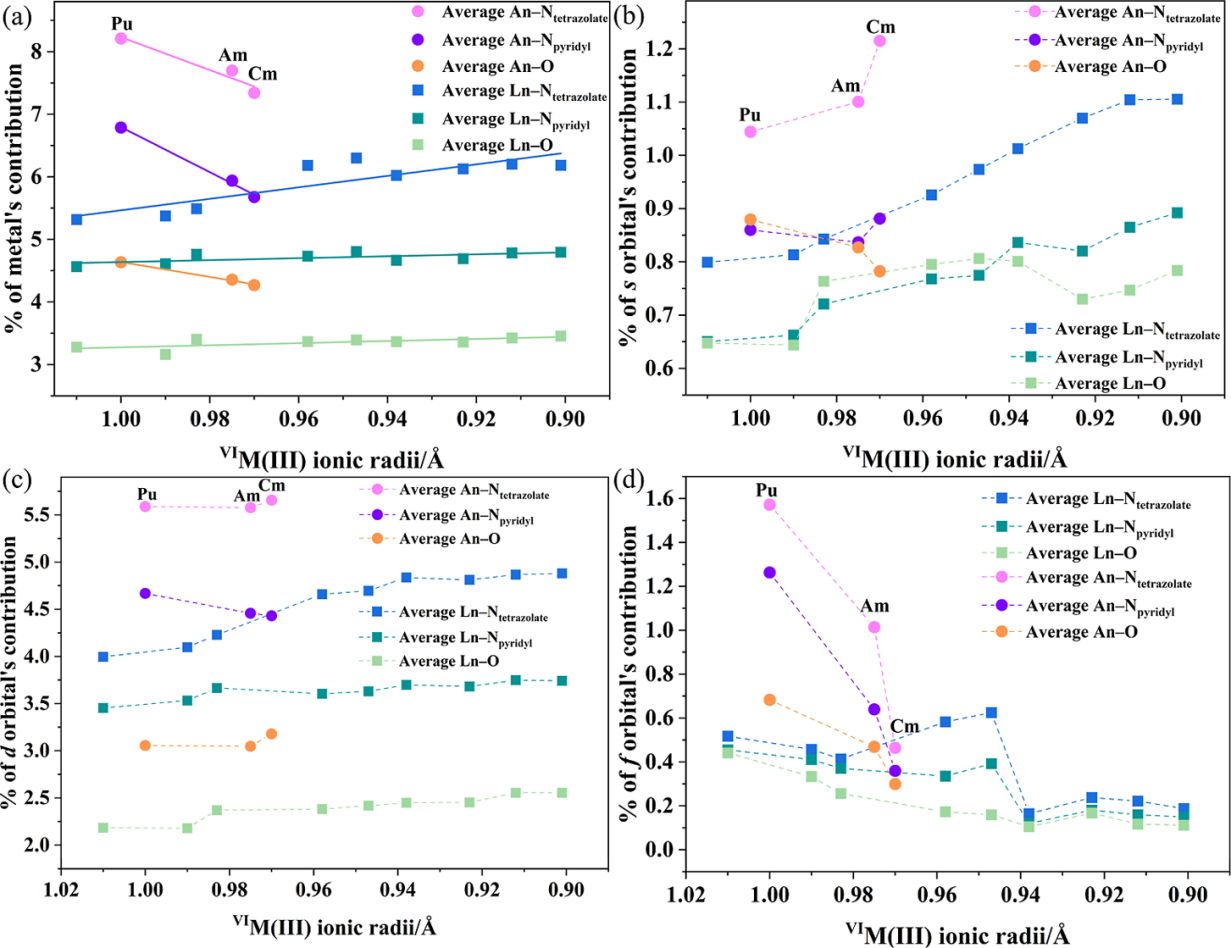
(a) Total metal contribution, (b) 6s/7s orbital contribution, (c) 5d/6d orbital contribution, and (d) 4f/5f orbital contribution of M–O, M–N_tetrazolate_, and M–N_pyridyl_ bonds.

The dominant metal contribution to the NLMOs of M–N bonds arises from d orbitals (5d for Ln^3+^, and 6d for An^3+^), that remain essentially constant across the series. The s orbital involvement (6s for Ln^3+^, and 7s for An^3+^) in NLMOs of M–N bonds shows a slight increase in M–N bonds. Conversely, f-orbital participation decreases across both the lanthanide and actinide series but at different rates. In actinides, it drops sharply, with M–O, M–N_tetrazolate_, and M–N_pyridyl_ contributions falling from 0.68% to 0.30%, 1.26% to 0.36%, and 1.57% to 0.46%, respectively. In contrast, lanthanides exhibit only minimal, slightly fluctuating reductions, ranging from 0.46% to 0.11%, as shown in Figure 5d.

In addition, Wiberg bond indices (WBIs) were calculated. As shown in Figure S10.1, while the Ln–OH_2_ and Ln–N bond lengths exhibit a linear decrease across the series due to the lanthanide contraction, their corresponding WBI bond orders remain relatively constant. This trend underscores the dominant role of electrostatic interactions in lanthanide chemistry, where 4f orbitals do not participate in bonding due to their localized spatial distribution. In contrast, although the Pu–L, Am–L, and Cm–L bond lengths fall within the margin of error of one another, the WBI bond orders for both An–OH_2_ and An–N bonds systematically decrease from **Pu1** to **Cm1**, consistent with decreased 5f-orbital participation. The divergence originates from the relativistic expansion of 5f orbitals in actinides, enabling variable covalent interactions and underscoring a fundamental dichotomy between lanthanide and actinide bonding paradigms.

Notably, the bond orders of the M–OH_2_ and M–N_pyridyl_ bonds are nearly identical, deviating from the general trend wherein ligands with increased polarizability typically exhibit greater orbital overlap and stronger covalent interactions with actinide ions. This anomaly contrasts with expectations from previous studies, showing that f orbitals share more electrons with softer ligands.^60^ The likely reason is that N donors are more diffuse than O donors, resulting in lower electron density within a fixed volume and consequently a decreased WBI. In addition, An–L bonds are stronger than corresponding Ln–L bonds, and average bond orders of M–N_tetrazolate_ bonds are greater than that of M–N_pyridyl_ bonds shown in Section S10. Exhibiting the same trend as the bond lengths, the average ΔWBI of the M–N_tetrazolate_ bonds between the actinides and their lanthanide analogs are slightly greater than that of the M–N_pyridyl_, for example, the M–N_tetrazolate_ ΔWBI average (0.0777) is slightly greater than the M–N_pyridyl_ ΔWBI (0.0595) average between **Pu1** and **Nd1**.

The molecular orbital diagram (Figure 6) indicates that as the 5f orbital energy decreases from Pu^3+^ to Cm^3+^, the 5f orbitals become more degenerate with the pdtz^–^ molecular orbitals, leading to enhanced energy degeneracy-driven covalency. This discovery aligns with findings in An­(dpa)_3_^3–^ complexes (An^3+^ = Am^3+^–Cf^3+^), where energy degeneracy-driven covalency between the 2p orbitals and 5f orbitals enables the later actinides to engage in more covalent interactions than the earlier ones, despite the decreasing 5f orbital energy across the series limiting orbital mixing.^18^ However, in [An­(mnt)_4_]^5–^ (An^3+^ = Np^3+^, Pu^3+^, Am^3+^, Cm^3+^, and Cf^3+^), the 5f orbitals are nearly energetically degenerate with sulfur 3p orbitals from neptunium to americium. For the later actinides, curium and californium, the 5f orbitals lie deeper in energy and instead become degenerate with the delocalized π orbitals of the mnt^2–^ ligand.^19^ These findings suggest that appropriate ligand selection is crucial for tuning energy degeneracy-driven covalency effects across the actinide series. This insight also has important implications for the rational design of ligands for actinide–lanthanide separations.

**Figure 6:**
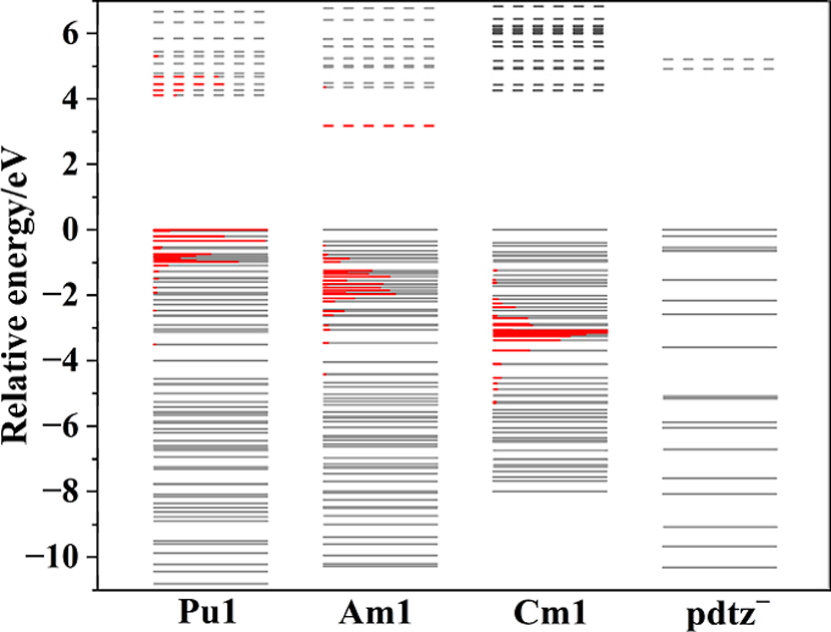
Molecular orbital energy diagram (normalized to the HOMO) of [M­(pdtz)_3_(H_2_O)_3_]·3.5H_2_O. Red bars indicate the 5f orbital contributions to the molecular orbitals, with their lengths proportional to the percentage contribution in each canonical MO. Solid and dashed lines represent occupied orbitals and unoccupied orbitals, respectively.

To further validate the reliability of the above results, the electronic structures of **Pu1**, **Am1**, and **Cm1** were examined using a complete active space multiconfigurational approach with second-order perturbative corrections (NEVPT2), as implemented in the ORCA 6.1.1 program.^61^ The CASSCF ground-state wave functions were subsequently used to analyze orbital compositions via natural localized molecular orbitals (NLMO) from the natural bond orbital (NBO) analysis,^62^ while molecular electron densities were examined using QTAIM calculations performed with MultiWFN.^63^ The resulting M–N bond orbital compositions and electron densities and detailed discussion for **Pu1**, **Am1**, and **Cm1** are presented in Section S11 and show trends consistent with those obtained from the discussed analysis. These results indicate that the DFT approach reasonably reproduces the multiconfigurational descriptions.

Overall, the electron density, energy density, and exchange-correlation component of the two-electron interaction energy for the An–N bonds indicate that the covalency remains nearly unchanged from **Pu1** to **Cm1**. However, analyses of delocalization indexes, 5f orbital participation, and WBIs reveal a decrease in metal and 5f orbital contributions to the An–N bonds from Pu^3+^ to Cm^3+^. The molecular orbital energy diagrams for the **Pu1**–**Cm1** complexes suggest an increasing energy degeneracy-driven covalency across this range. Additionally, M–N_tetrazolate_ bonds exhibit greater covalency than M–N_pyridyl_ bonds. The trends in bond lengths, metal contributions, and f-orbital involvement differ markedly between the lanthanide and actinide series, with An–N bonds showing covalency stronger than that of their Ln–N counterparts.

To gain a more comprehensive understanding of these newly synthesized transuranium compounds, absorption spectra were recorded for both the solid-state and solution phases of [An­(pdtz)_3_(H_2_O)_3_]·3.5H_2_O (An^3+^ = Pu^3+^, Am^3+^, and Cm^3+^). Single crystals and DMSO solutions were collected using a CRAIC microspectrophotometer and an Agilent Technologies Cary Series 6000i UV–vis–NIR spectrophotometer, respectively. The overlaid spectra from the solid-state and solution phases display the characteristic Laporte-forbidden 5f → 5f transitions of Pu­(III), Am­(III), and Cm­(III). The solution-phase UV–vis–NIR spectra exhibit broader peaks, attributable to the possibility that DMSO is exchanged with the coordinating water in the metal inner sphere. The assignment to be discussed here is based on reported compounds, and the fully quantitative assignments should be conducted by combining calculations, taking the influence of the ligand field and spin–orbit coupling into account.

As shown in Figure 7a, the solid-state and solution-phase spectra of **Pu1** display the characteristic Laporte-forbidden 5f → 5f transitions of Pu­(III) from the predominantly ^6^H_5/2_ ground state, consistent with reported Pu­(III) compounds such as ^242^Pu_2_(C_6_(CO_2_)_6_)­(H_2_O)_9_·H_2_O, ^242^Pu_2_(C_6_(CO_2_)_6_)­(H_2_O)_8_·2H_2_O,^46^ [(H_3_O)­(18-crown-6)]­[Pu­(H_2_O)_4_(18-crown-6)]­(ClO_4_)_4_ · 2H_2_O,^64^ etc. The Russell–Saunders term assignments for these transitions are based on calculations for PuCl_3_ by Carnall and co-workers.^65^ Higher-energy transitions are obscured by metal-to-ligand charge transfer (MLCT) bands in the range of 21,500 cm^–1^ to 31,500 cm^–1^ (Figure S12.1). Two of the most intense Pu^3+^ peaks are observed for group L and group M (^6^H_5/2_ → ^4^L_13/2_, ^4^K_11/2_, ^4^I_9/2_, ^4^P_5/2_) at 17,286 cm^–1^ and group K (^6^H_5/2_ → ^4^ M_15/2_) at 16,352 cm^–1^. Additional intense 5f → 5f transitions characteristic of Pu^3+^ are also observed in both phases, including group H (^6^H_5/2_ → ^6^F_11/2_), 14,911 cm^–1^; group F (^6^H_5/2_ → ^6^H_15/2_, ^6^F_9/2_), in the range 11,711 cm^–1^–13,473 cm^–1^; group E (^6^H_5/2_ → ^6^H_13/2_) at 11,090 cm^–1^; group D (^6^H_5/2_ → ^6^F_7/2_) at 9,769 cm^–1^; group C (^6^H_5/2_ → ^6^H_11/2_) at 9,040 cm^–1^; group B (^6^H_5/2_ → ^6^F_3/2_, ^6^H_5/2_, ^6^H_9/2_) in the range 6,000 cm^–1^–7,470 cm^–1^.

**Figure 7:**
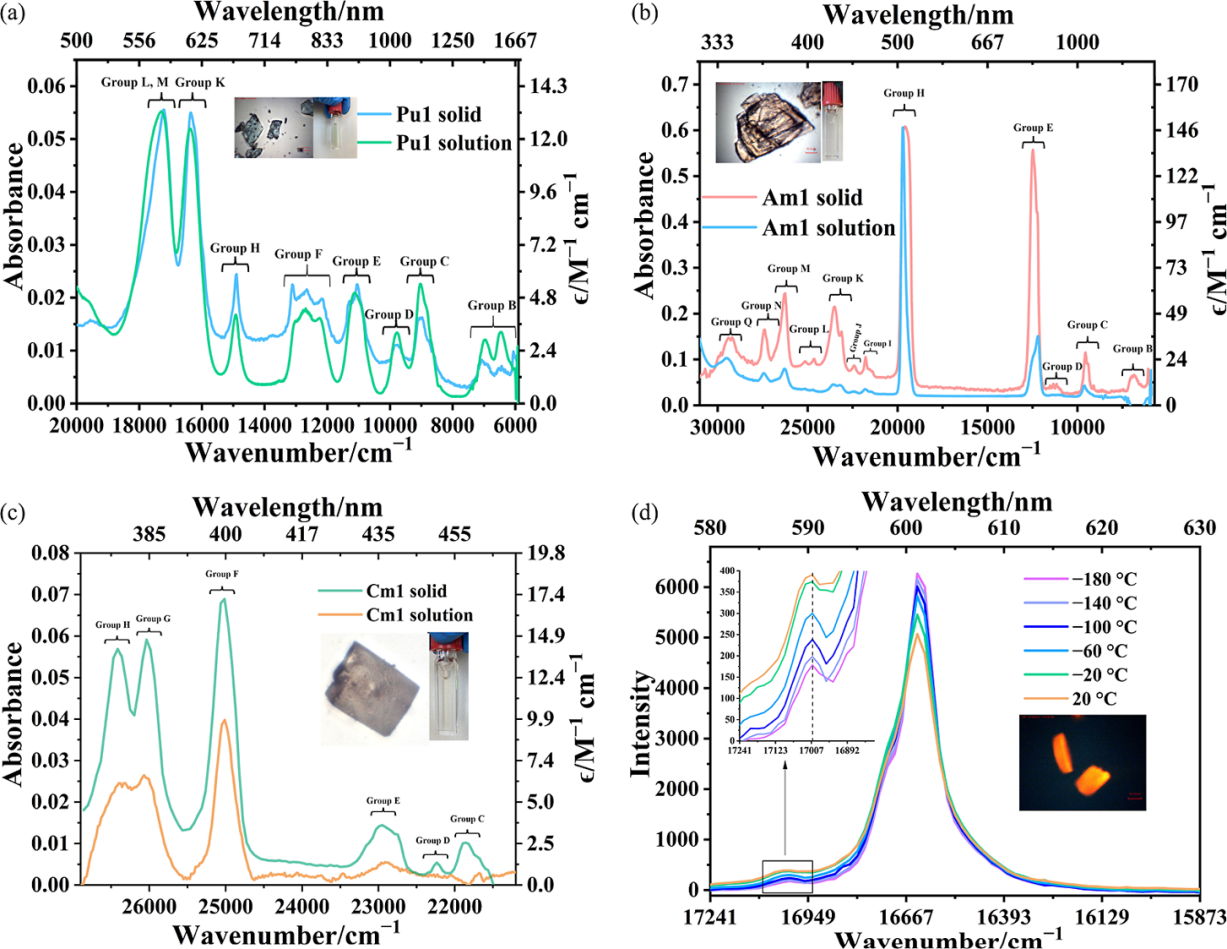
Solid-state and solution-phase (in DMSO) absorption UV–vis–NIR spectra of (a) **Pu1**, (b) **Am1**, and (c) **Cm1** at room temperature (inserted are images of the crystals and solutions used for collection). (d) Temperature-dependent phosphorescence spectra of **Cm1**. (Inserted are crystals and the peaks from 580 to 595 nm.)

The transition assignments of **Am1** are based on calculated Am^3+^ transitions in AmCl_3_ ^66^ reported by Carnall and co-workers. Like recently reported trivalent compounds,^67,68^ the room-temperature absorption spectra of **Am1**, in both solid and solution phases, exhibit three characteristic Laporte-forbidden 5f → 5f transitions of the Am^3+^ ion, hypersensitive group H, ^7^F_0_ → ^5^L_6_, at 19,591 cm^–1^; group E, ^7^F_0_ → ^7^F_6_, at 12,490 cm^–1^; and group C, ^7^F_0_ → ^7^F_4_, at 9,538 cm^–1^. Seven additional weak absorption groups appear in the higher-energy range of 21,100–30,800 cm^–1^ corresponding well with the calculated Am^3+^ transitions in AmCl_3_.^66^ These are typically assigned as follows: Group I, ^7^F_0_ → ^5^D_2_, ^5^G_2_ (21,272 cm^–1^–22,067 cm^–1^); group J, ^7^F_0_ → ^5^H_3_ (22,408 cm^–1^); group K, ^7^F_0_ → ^5^H_5,4_ (split, 22,714 cm^–1^–24,332 cm^–1^); group L, ^7^F_0_ → ^5^D_3_, ^5^L_6_ (24,660 cm^–1^, 25,151 cm^–1^); group M, ^7^F_0_ → ^5^G_4_, ^5^L_8_ (26,270 cm^–1^); groups N and O, ^7^F_0_ → ^5^G_2,5_ (27,413 cm^–1^); and group Q, ^7^F_0_ → ^5^I_4_, ^5^I_6_, ^5^H_5,4_ (28,670 cm^–1^–30,357 cm^–1^). In the solid state, group B, ^7^F_0_ → ^7^F_3_, at 6,854 cm^–1^ and group D, ^7^F_0_ → ^5^L_5_, from 10,914 cm^–1^ to 11,671 cm^–1^ are also observed but have low intensity.

The absorption spectra of **Cm1** are predominantly observed in the visible region, a consequence of its half-filled 5f^7^ electron configuration. Energy-level group assignments are based on the free-ion energy-level scheme of Cm^3+^_(aq)_ reported by Carnall and Rajnak.^69^ The **Cm1** absorption spectra in the solid and in DMSO display sharp, well-defined 5f → 5f transitions consistent with those reported for the Cm^3+^ ion in perchloric acid media^69^ [Cm­(H_2_O)_8_]­(Hdtp)­(dtp)·H_2_O (H_2_dtp = 2,3-di­(tetrazol-5-yl)­pyrazine)^70^ [Cm­(H_2_O)_9_]­[CF_3_SO_3_]_3_,^71^ Cm­(S_2_CNEt_2_)_3_(N_2_C_12_H_8_),^28^ Cm_2_[(C_6_(CO_2_)_6_]­(H_2_O)_8_·2H_2_O,^47^ [(Cm­(pmtz)_2_(H_2_O)_3_)_2_(μ-pmtz)]_2_(pmtz)_2_·26H_2_O,^54^ and [Cm­(pydtc)_4_]^−^ (pydtc^–^ = pyrrolidinedithiocarbamate).^47^ In both solid and solution phases, group E, at 22,934 cm^–1^ (^8^S_7/2_ → ^6^I_9/2_), group F at 25,021 cm^–1^ (^8^S_7/2_ → ^6^I_11/2,17/2_), group G, at 26,019 cm^–1^ (^8^S_7/2_ → ^6^I_13/2_ and ^6^D_9/2_), and group H, at 26,411 cm^–1^ (^8^S_7/2_ → ^6^I_15/2_) are observed. In the solid state, two additional weak transitions are assigned as group C (^8^S_7/2_ → ^6^I_7/2_), at 21,846 cm^–1^, and group D (^8^S_7/2_ → ^6^P_3/2_), at 22,231 cm^–1^.

Photoluminescence spectra of **Cm1** acquired as a function of temperature ranging from 20 °C to −180 °C in 40 °C increments with 420 nm excitation (Figure 7d) show the primary peak centered at 16,622 cm^–1^ (601.6 nm), close to the value observed for [(Cm­(pmtz)_2_(H_2_O)_3_)_2_(μ-pmtz)]_2_(pmtz)_2_·14H_2_O (λ_max_ = 16,570 cm^–1^).^54^ **Cm1** emits at lower energy than [Cm­(H_2_O)_9_]­[CF_3_SO_3_]_3_ (λ_max_ = 16,900 cm^–1^),^71^ [Cm­(H_2_O)_8_]­(Hdtp)­(dtp)·H_2_O (λ_max_ = 16,808 cm^–1^)^70^ and Cm^3+^ in aqueous media at room temperature (λ_max_ = 16,840 cm^–1^)^72^ because these only contain Cm–OH_2_ bonds. Likewise, Cm1 is expected to emit at higher energy than Cm­(Hdpa)_3_·H_2_O (λ_max_ = 16,366 cm^–1^),^17^ [Cm­(Hdpa)­(H_2_dpa)­(H_2_O)_2_Cl]­Cl·2H_2_O (λ_max_ = 16,666 cm^–1^),^53^ Cm­(S_2_CNEt_2_)_3_(N_2_C_12_H_8_) (λ_max_ = 16,366 cm^–1^) because these contain Cm–N and Cm–S bonds,^28^ as well as [NH_4_]­[Cm­(pydtc)_4_]·2CH_3_OH (λ_max_ = 16,366 cm^–1^) that only possesses Cm–S bonds^47^ and Cp′_3_Cm (λ_max_ = ∼15,723 cm^–1^), with Cm–C bonds.^73^ Being consistent with some of the compounds mentioned above, cooling from 20 to −180 °C results in a decrease in vibrational relaxation leading to a greater emission intensity without shifting the peak position (Figure 7d). It should be noted that photoluminescence spectra show a large splitting of 381 cm^–1^ of the primary peak. In comparison, a splitting of 743 cm^–1^ is observed for [NH_4_]­[Cm­(pydtc)_4_]·2CH_3_OH^47^ due to ligand-field strength of the dithiocarbamate ligands.^74^

## Conclusions

In summary, the combination of detailed structural and spectroscopic data with a quatum mechanical analyses of [M­(pdtz)_3_(H_2_O)_3_]·3.5H_2_O (M^3+^ = Pu^3+^–Cm^3+^, La^3+^–Nd^3+^, and Sm^3+^–Ho^3+^) reveals distinct bonding trends between the actinide and lanthanide complexes. The actinide M–OH_2_ and M–N bond lengths remain nearly constant across the Pu^3+^ to Cm^3+^ series, while the lanthanide bond lengths decrease nearly linearly across the 4f-block. Computational analyses indicate that the overall actinide covalency across the Pu^3+^ to Cm^3+^ series is largely maintained because while the contributions of the 5f orbitals to An–L bonds decreases, the 5f metal orbitals and ligand 2p orbitals become more degenerate in energy.

Considering the preceding data, it can be inferred that 5f orbitals in the early actinides (prior to Am) exhibit stronger energy degeneracy driven covalency with 3p orbitals in sulfur-based ligands (mnt^2–^ = maleonitrile-1,2-dithiolate); whereas 5f orbitals in the later actinides (after Am) display greater energy degeneracy covalency with 2p orbitals from nitrogen- or oxygen-based ligands (*e.g.* in dpa^2–^ = 2,6-pyridinedicarboxylate and pdtz^–^ = 5-(2-pyridyl)-1*H*-tetrazolate complexes). Additionally, all M–N_tetrazolate_ bonds are consistently shorter and more covalent than M–N_pyridyl_ bonds, highlighting the influence of ligand choice on bond character. This also reveals a correlation between nitrogen electronegativity and the degree of M–N covalency. These findings provide insights into the tuning of energy degeneracy-driven covalency across the actinide series, thereby facilitating the development of more selective extractants for actinide elements.

## Supplementary Material



## Notes

Additional images of reactions and crystals, crystallographic data, crystal structures, PXRD, Raman spectra, absorption spectra, ^1^H NMR spectra of **Ln1**, SHAPE results, additional bond length analysis, computational data generated in this study, and a table of N-donor ligands for practical An/Ln separations (PDF)
