# Supplementary material for: Covalency of M–N Bonds in Isomorphous Lanthanide and Actinide 5‑(2-Pyridyl)‑1H‑tetrazolate Complexes
Source: JACS Au. 2026 Feb 27;6(3):1563–72. doi: 10.1021/jacsau.5c01374 (PMC13014186; doi:10.1021/jacsau.5c01374)
Supplement: Supplementary file 1 [file au5c01374_si_001.pdf]

Supporting Information

Covalency of M–N Bonds in Isomorphous Lanthanide and Actinide 5-(2-Pyridyl)-1*H*-Tetrazolate Complexes

Zhuanling Bai, Madeline C. Martelles, Qiang Gao, Nicholas B. Beck, Jacob P. Brannon, Joseph M. Sperling,* Thomas E. Albrecht*

Department of Chemistry and Nuclear Science and Engineering Center, Colorado School of Mines, Golden, Colorado 80401, United States

*Corresponding authors. Email: jsperling@mines.edu; thomas.albrecht@mines.edu

Contents

Section S1: Experimental, computational details and images of reactions....................3
Section S2: Crystallographic data, PXRD, and spectra of **Ln1** 10
Section S3: Crystallographic data for **Pu1**, **Am1** and **Cm1**..19
Section S4: Additional crystal structures 22
Section S5: SHAPE results 24
Section S6: Additional bond length analysis 25
Section S7: The quantum theory of atoms in molecules (QTAIM) and natural localized molecular orbital (NLMO) 28
Section S8: Wiberg bond indices (WBIs) 37
Section S9: Interacting quantum atoms (IQA)..39
Section S10: Absorption UV-vis-NIR spectrum of **Pu1**..42
Section S11: CASSCF Calculations...43
Section S12: Cartesian coordinates for all structures..59
Section S13: Table of N-donors' ligands for practical An/Ln separation....................76
Section S14: Reference 77

Section S1: Experimental, computational details and images of reactions

Method

Caution! ^{239}Pu ($t_{1/2}$ = 24,065 years) poses severe health risks due to its α emission and the radiotoxicity from its α-, β-, and γ-emitting daughters. ^{243}Am ($t_{1/2}$ = 7,370 years) also presents significant health dangers through its α and γ-emitting, along with risks from a short-lived, high-energy β- and γ-emitting ^{239}Np ($t_{1/2}$ = 2.356 days) daughter product. ^{248}Cm, ($t_{1/2}$ = 348,000 y, specific activity = 4.14 μCi/mg) is notably hazardous due to its intense α-particle emission and nearly 8% spontaneous fission rate, generating a high neutron flux that is challenging to shield and elevate the biological dose by a factor of ten compared to typical γ-emitting isotopes in transuranium facilities. Consequently, all experiments involving these isotopes were carried out in a radiation lab equipped with a HEPA filtered hood. As synthesized products were only handled when coated with solvent or immersion oil, and radiation levels were continuously monitored following all characterizations.

Materials

5-(2-pyridyl)-1*H*-tetrazole (**Hpdtz**) (97%, Sigma-Aldrich), DI H_2O, Dimethyl sulfoxide-d6 (99.9 atom % D, Sigma-Aldrich), Deuterium oxide (D_2O, 99.9 atom % D, Sigma-Aldrich), HCl (37%, Sigma-Aldrich), $HBr_{(aq)}$ (48%, Sigma-Aldrich), diethyl ether (99%, Fisher Scientific) NH_4OH (32%, Sigma-Aldrich), are all used as received. lanthanide sesquioxide (99.8%, Sigma-Aldrich). $LnCl_3·6H_2O$ were synthesized by dissolving lanthanide sesquioxide in 12 M aqueous HCl and then evaporating it to supersaturation at 150 °C in a box furnace. Upon agitation of the solution, $LnCl_3·6H_2O$ crystals formed immediately. ^{243}Am and ^{248}Cm were provided by the Radiochemical Engineering Development Center (REDC) at Oak Ridge National Laboratory (ORNL).

Synthesis

$[Ln(pdtz)_3(H_2O)_3]·3.5H_2O$: A mixture containing 0.1 mmol of $LnCl_3·6H_2O$ in 0.1 mL of DI water and 0.045 g (0.3 mmol) of Hpdtz dissolved in 0.7 mL of hot 0.1 M NaOH solution was allowed to sit at room temperature for 12 hours, leading to the formation of crystalline solids. They were washed with cold distilled water (3 × 1 mL), isolated through filtration, and then dried at room temperature. These solids are appropriate for single crystal X-ray diffraction analysis.

La: 43 mg (0.062 mmol) $[La(pdtz)_3(H_2O)_3]·3.5H_2O$, 62% based on $LaCl_3·6H_2O$.
Ce: 58 mg (0.082 mmol) $[Ce(pdtz)_3(H_2O)_3]·3.5H_2O$, 82% based on $CeCl_3·6H_2O$.
Pr: 63 mg (0.09 mmol) $[Pr(pdtz)_3(H_2O)_3]·3.5H_2O$, 90% based on $PrCl_3·6H_2O$.
Nd: 60 mg (0.086 mmol) $[Nd(pdtz)_3(H_2O)_3]·3.5H_2O$, 86% based on $NdCl_3·6H_2O$.
Sm: 62 mg (0.088 mmol) $[Sm(pdtz)_3(H_2O)_3]·3.5H_2O$, 88% based on $SmCl_3·6H_2O$.
Eu: 64 mg (0.09 mmol) $[Eu(pdtz)_3(H_2O)_3]·3.5H_2O$, 90% based on $EuCl_3·6H_2O$.
Gd: 76 mg (0.092 mmol) $[Gd(pdtz)_3(H_2O)_3]·3.5H_2O$, 92% based on $GdCl_3·6H_2O$.
Tb: 63 mg (0.089 mmol) $[Tb(pdtz)_3(H_2O)_3]·3.5H_2O$, 89% based on $TbCl_3·6H_2O$.
Dy: 62 mg (0.086 mmol) $[Dy(pdtz)_3(H_2O)_3]·3.5H_2O$, 86% based on $DyCl_3·6H_2O$.
Ho: 51 mg (0.072 mmol) $[Ho((pdtz)_3(H_2O)_3]·3H_2O$, 72% based on $HoCl_3·6H_2O$.

The solid-state absorption UV-vis-NIR and Raman spectra of these compounds are shown in **Section S2**. The assignments of these 4*f* → 4*f* transitions can reference recently reported Lanthanide pyrithione complexes.[1]

Synthesis of Pu1: **[^{239}Pu(pdtz)$_3$(H$_2$O)$_3$]·3.5H$_2$O**. A 0.3 mL stock solution of ^{239}Pu stock solution (10 mg/1 mL HCl (1 M), containing 3 mg ^{239}Pu, 0.0126 mmol) was evaporated to dryness in a 20 mL scintillation vial. The residue was then dissolved in 1 mL of DI water and transferred to a centrifuge tube. After adding an excess of $NH_4OH_{(aq)}$, the mixture was centrifuged, and the resultant solid was washed three times with 2 mL of DI water each. This solid was subsequently dissolved in an excess of $HBr_{(aq)}$, and the solution was gently evaporated under a heat lamp while a stream of nitrogen was passed over the vial to form solid $^{239}PuBr_3 \cdot nH_2O$. This solid was washed three times with diethyl ether (2 mL each time) and then redissolved in 0.2 mL of DI water. To this solution, 0.01 g (0.075 mmol) of Hpdtz dissolved in 0.35 mL of hot 0.2 M NaOH was added. Crystals suitable for single crystal X-ray diffraction formed within 10 minutes. Subsequently, the crystals were separated from the mother liquor to avoid co-crystallization with NaBr. The solid state and solution phase (in DMSO) absorption UV-vis-NIR spectra of **Pu1** are shown in **Figure 7a**.

Synthesis of Am1: **[^{243}Am(pdtz)$_3$(H$_2$O)$_3$]·3.5H$_2$O**. A 0.5 mL stock solution of ^{243}Am stock solution (2 mg/mL HCl (1 M), containing 1 mg ^{243}Am, 0.0041 mmol) was evaporated to a residue in a 20 mL scintillation vial. The resulting residue was dissolved in 0.3 mL DI water and transferred into a shell vial. A solution of Hpdtz (4 mg, 0.03 mmol) in 0.15 mL hot 0.2 M NaOH was added to the Am(III) solution, yielding crystals suitable for single-crystal X-ray diffraction within 6 hours. The crystals were then removed from the mother liquor to ensure that sodium chloride did not co-crystallize. The solid state and solution phase (in DMSO) absorption UV-vis-NIR spectra of **Am1** are shown in **Figure 7b**.

Synthesis of Cm1: **[^{248}Cm(pdtz)$_3$(H$_2$O)$_3$]·3.5H$_2$O**. ^{248}Cm solid after conducting a series of synthesis experiments was dissolved in 2 M HCl and the resulting solution was treated with excess concentrated NH_4OH to precipitate $^{248}Cm(OH)_3$. The precipitate was collected by centrifugation (1000 rpm, 15 min), rinsed three times with DI water, and redissolved in 2 M HCl to afford a $^{248}CmCl_3$ solution (about 1 mg, 0.004 mmol, ^{248}Cm content). The solution evaporated to dryness, and the residue was dissolved in 0.3 mL DI water. A solution of Hpdtz (4 mg, 0.03 mmol) in 0.15 mL hot 0.2 M NaOH was added, and the mixture was left to stand overnight, yielding crystals suitable for single-crystal X-ray diffraction. Subsequently, the crystals were separated from the mother liquor to avoid co-crystallization with sodium chloride. The solid state and solution phase (in DMSO) absorption and phosphorescence spectra of **Cm1** are shown in **Figure 7c and 6d**.

Crystallographic Studies

Single crystals were carefully selected under immersion oil and mounted on a MiTeGen micro loop. They were then aligned with the incident X-ray beam using a digital camera connected to the APEX4 software[2] on a Bruker D8 Quest single crystal X-ray diffractometer. Collection strategies were devised using Mo K_α X-ray radiation (λ = 0.71073 Å) at a temperature of 100 K. The evaluation, integration, and reduction of the diffraction data were conducted using the APEX4 software suite.[2] The diffraction data underwent absorption correction using the multi-scan method with the software

Multiscan SADABS 2016/2. Structures were subsequently refined utilizing direct methods through the SHELXTL suite in the program *OLEX-2*.[4–6] CCDC deposition numbers 2420699-2420711 contain detailed crystallographic data.

Powder X-ray Diffraction (PXRD) data were collected from 5 to 50° with a step of 0.02° and the time for data collection was 1 s on a Powder XRD-Bruker diffractometer with Cu K_{α} radiation (λ= 1.54056 Å) source at 30 kV and 30 mA, divergent beam optics, and a Rigaku D/tex detector at room temperature. The experimental PXRD results were compared to simulated spectra generated using the Cambridge Crystallographic Data Centre Mercury software (Mercury 2024.2.0 (Build 415171)).[7]

Spectroscopy

Solid UV-vis-NIR Spectroscopy. Absorbance and photoluminescence measurements in the UV-vis-NIR region were performed on single crystals on a glass slide coated with Paratone-N oil using a Craic 20/20 PVTM dual microspectrophotometer, employing a mercury lamp for absorption and a xenon lamp for luminescence. The absorbance data collection durations were optimized using Craic Technologies Minerva software, and spectra were recorded in the range of 320 to 1,700 nm, utilizing a 75 W Xenon lamp as the light source.

For variable-temperature photoluminescence measurements, selected crystals were transferred to a quartz slide and positioned in a Linkam LTS420 temperature-controlled stage on the CRAIC microspectrophotometer. The stage was cooled to the target temperature at 5 °C/min and then purged with nitrogen for 5 minutes prior to data acquisition. The photoluminescence data were collected in 40 °C increments from 20 °C to −180 °C.

Solution UV-vis-NIR Spectroscopy. Solution samples were analyzed using an Agilent Technologies Cary Series UV-Vis-NIR spectrophotometer 6000i, utilizing small volume quartz cuvettes at a resolution of 0.1 nm. As-synthesized crystals were dissolved in 2 mL DMSO. The concentration of **Pu1**, **Am1** and **Cm1** are about 1.5 mg/mL (0.006 mmol/mL), 0.5 mg/mL (0.002 mmol/mL), 0.5 mg/mL (0.002 mmol/mL), respectively.

Raman spectra. Solid-state Raman spectra of **Ln1** were collected using a Bruker SENTERRA II Raman Spectrograph, equipped with OPUS software (version 8.7, Build 8.7.41). Spectra were collected over the ranges 45–1450 cm^{-1} and 50–3630 cm^{-1}, with spectral resolutions of 1.5 and 4 cm^{-1}, respectively. Excitation was provided by a 785 nm laser (100 mW), using two coadditions and an integration time of 1000 ms. Dry crystal samples were prepared on glass slides and mounted on the instrument stage for measurement.

^{1}H NMR. ^{1}H NMR spectra were collected on a JEOL ECA-500 NMR Spectrometer using D_2O as the solvent, with residual solvent serving as the internal reference. Crystalline samples were dissolved in D_2O, loaded into P535 NMR tubes, and measured at room temperature. It should be noted that the solubility of **Gd1**, **Tb1**, **Dy1**, and **Ho1** in D_2O is extremely low and easy to recrystallize, resulting in correspondingly weak ^{1}H NMR signal intensities. We also attempted dissolution in DMSO-d_6 (Dimethyl sulfoxide-d_6); however, higher concentrations did not improve

signal quality because the strong paramagnetic of these trivalent lanthanides leads to rapid nuclear relaxation and severe line broadening, often rendering signals unobservable.

Computational Details.

All calculations were based on experimentally determined single-crystal X-ray diffraction (XRD) structures. Wiberg bond indices (WBIs), natural localized molecular orbitals (NLMOs) (the NBO6 program[8] linked to ADF, and Quantum Theory of Atoms in Molecules (QTAIM)[9] were performed using the Amsterdam Density Functional (ADF) engine within the AMS 2024.106 software. The hybrid generalized gradient approximation (GGA) functional PBE0[10] was used to compute the electron density for the QTAIM analysis.[9] A triple-ζ Slater-type orbital basis set (STO-TZ2P)[11] was employed. Relativistic effects were treated using the scalar relativistic zero-order regular approximation (ZORA).[12] Numerical quality was set to “very good,” with the analysis level set to “Full” and a grid spacing of 0.2 Bohr. The NLMO calculations employed the PBE0 functional[10] with no frozen-core approximation, and the TZ2P basis set[11] was used for all atoms. Scalar relativistic effects were accounted for via the zero-order regular approximation (ZORA).[12] Computational accuracy was set to "very good." Interacting Quantum Atom (IQA) analysis was carried out with AIMAll (19.10.12, Professional)[13] using all-electron wavefunctions. These wavefunctions were obtained from single-point energy calculations on the XRD structures, performed in ORCA[14] and processed with the ‘orca_2aim’ from the single-point output.

It should be noted that DFT calculations for actinide coordination chemistry have inherent limitations. They provide a topological analysis of the ground-state electron density, offering insights into bonding and atomic interactions, but do not capture excited states or multiconfigurational effects, which are particularly important for heavy elements like actinides and lanthanides. Additionally, results can be sensitive to the quality of the electron density, and small variations in geometry can significantly affect bond critical points and derived properties. Therefore, in this study, we rely directly on the experimentally determined crystal structures of all *f*-block complexes.

Images of reactions and crystals

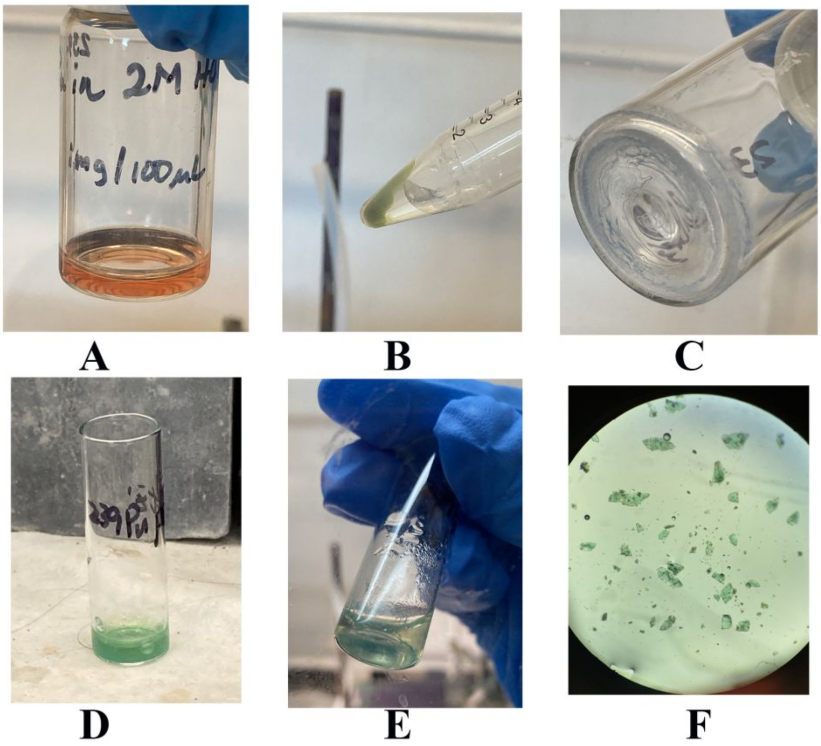

Figure. S1.1. A. Stock solution of $^{239}Pu^{3+}$ (1 mg/0.1 mL). B. $^{239}Pu(OH)_x$ after centrifuging. C. $^{239}PuBr_3{\cdot}nH_2O$. D. Reaction solution. E. Crystals of **Pu1** grown from aqueous solution overnight. F. Crystals of **Pu1** used for single-crystal X-ray diffraction and solid-state UV-vis-NIR spectroscopy.

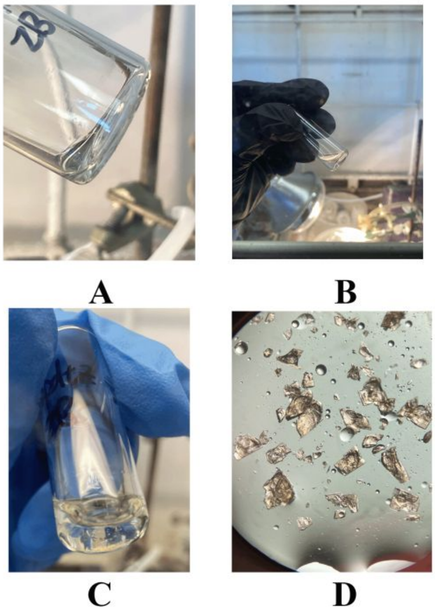

Figure S1.2. A. Stock solution of $^{243}Am^{3+}$ (2 mg/1 mL). B. Reaction solution. C. Crystals of **Am1** grown from aqueous solution overnight. D. Crystals of **Am1** used for single-crystal X-ray diffraction and solid-state UV-vis-NIR spectroscopy.

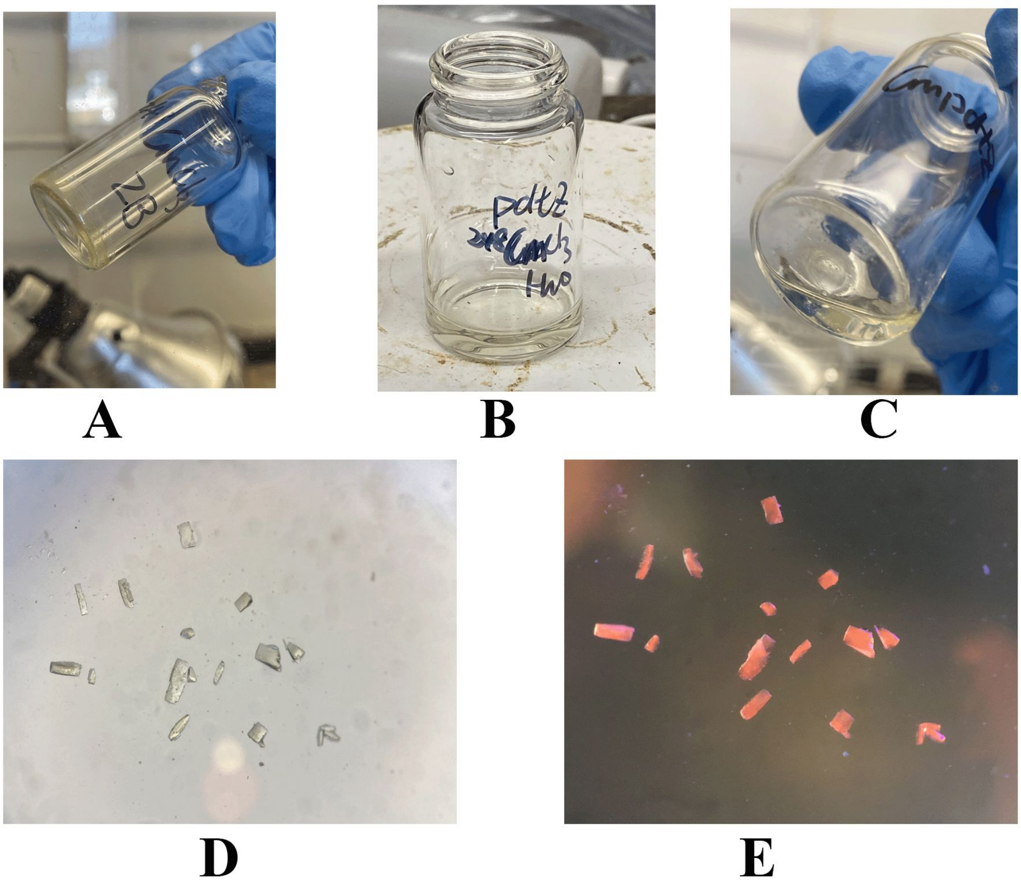

Figure S1.3. A. Solid of $^{248}CmCl_3$. B. Reaction solution. C. Crystals of **Cm1** grown from aqueous solution overnight. D. Crystals of **Cm1** used for single-crystal X-ray diffraction and solid-state UV-vis-NIR spectroscopy. E. Photoluminescence of **Cm1**.

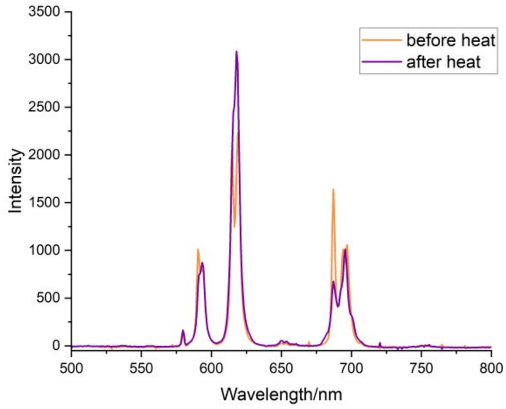

Figure S1.4. Phosphorescence spectra of **Eu1** before and after heating at 100 °C.

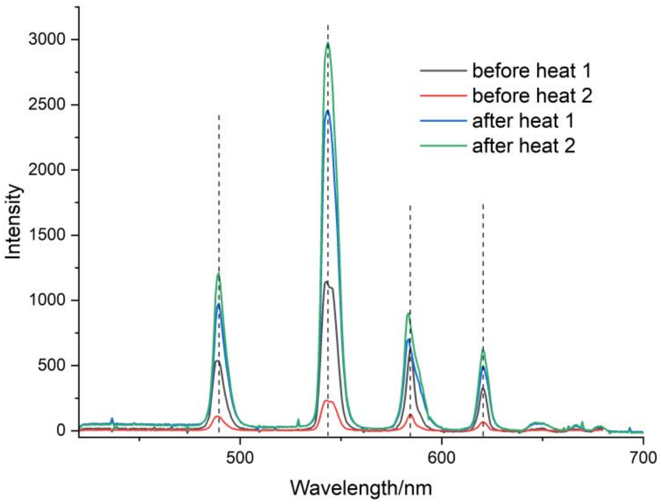

Figure S1.5. Phosphorescence spectra of **Tb1** before and after heating at 100 °C.

To investigate whether the removal of lattice water molecules induces structural changes that could affect the spectroscopic properties, **Eu1** and **Tb1** were heated at 100 °C for approximately 2 hours, after which their photoluminescence (PL) spectra were recorded. As shown in **Figures S1.4** and **S1.5**, no significant changes were observed for **Eu1** and **Tb1**.

Section S2: Crystallographic data, PXRD, and spectra of Ln1

Table S2.1. Crystal data and structure refinement.

Identification code	**La1**	**Ce1**
Empirical formula	$C_{18}H_{25}LaN_{15}O_{6.5}$	$C_{18}H_{25}CeN_{15}O_{6.5}$
Formula weight/g·mol^{-1}	694.44	695.65
Temperature/K	100	100
Crystal system	monoclinic	monoclinic
Space group	$P2_1/n$	$P2_1/n$
a/Å	9.1928(4)	9.1386(3)
b/Å	17.7266(8)	17.7271(7)
c/Å	16.9315(8)	16.8916(7)
α/°	90	90
β/°	99.5270(10)	99.5620(10)
γ/°	90	90
Volume/Å^3	2721.1(2)	2698.43(18)
Z	4	4
ρ_{calc}g/cm^3	1.695	1.712
μ/mm^{-1}	1.636	1.753
F(000)	1388.0	1392.0
Crystal size/mm^3	0.365 × 0.261 × 0.254	0.191 × 0.148 × 0.076
Radiation	Mo K_α (λ = 0.71073 Å)	Mo K_α (λ = 0.71073 Å)
2θ range/°	4.744 to 61.11	4.768 to 61.134
Index ranges	$-13 \le h \le 13$, $-25 \le k \le 25$, $-24 \le l \le 24$	$-13 \le h \le 13$, $-25 \le k \le 25$, $-21 \le l \le 24$
Reflections collected	69010	70259
Independent reflections	8332 [R_{int} = 0.0341, R_{sigma} = 0.0190]	8259 [R_{int} = 0.0487, R_{sigma} = 0.0266]
Data/restraints/parameters	8332/0/387	8259/0/387
Goodness-of-fit on F^2	1.141	1.193
Final R indexes [I≥2σ (I)]	R_1 = 0.0265, wR_2 = 0.0596	R_1 = 0.0353, wR_2 = 0.0689
Final R indexes [all data]	R_1 = 0.0277, wR_2 = 0.0601	R_1 = 0.0395, wR_2 = 0.0702
Largest diff. peak/hole/e Å^{-3}	2.60/−0.99	1.92/−2.13
Identification code	**Pr1**	**Nd1**
Empirical formula	$C_{18}H_{25}N_{15}O_{6.5}Pr$	$C_{18}H_{25}N_{15}NdO_{6.5}$
Formula weight/g·mol^{-1}	696.44	699.77
Temperature/K	100	100.00
Crystal system	monoclinic	monoclinic
Space group	$P2_1/n$	$P2_1/n$
a/Å	9.1190(2)	9.0812(4)
b/Å	17.7054(5)	17.6987(6)
c/Å	16.8555(4)	16.8054(6)
α/°	90	90

β/°	99.6400(10)	99.6790(10)
γ/°	90	90
Volume/Å^3	2682.99(12)	2662.61(18)
Z	4	4
ρ_{calc}g/cm^3	1.724	1.746
μ/mm^{-1}	1.883	2.017
F(000)	1396.0	1400.0
Crystal size/mm^3	0.173 × 0.162 × 0.057	0.21 × 0.137 × 0.077
Radiation	Mo K_α (λ = 0.71073 Å)	Mo K_α (λ = 0.71073 Å
2θ range/°	4.776 to 61.12	4.602 to 59.19
Index ranges	$-13 \le h \le 13$, $-25 \le k \le 25$, $-24 \le l \le 23$	$-12 \le h \le 12$, $-24 \le k \le 24$, $-23 \le l \le 23$
Reflections collected	69612	81426
Independent reflections	8201 [R_{int} = 0.0400, R_{sigma} = 0.0230]	7492 [R_{int} = 0.0255, R_{sigma} = 0.0117]
Data/restraints/parameters	8201/6/396	7492/0/459
Goodness-of-fit on F^2	1.073	1.238
Final R indexes [I≥2σ (I)]	R_1 = 0.0258, wR_2 = 0.0599	R_1 = 0.0202, wR_2 = 0.0422
Final R indexes [all data]	R_1 = 0.0292, wR_2 = 0.0614	R_1 = 0.0206, wR_2 = 0.0423
Largest diff. peak/hole/e Å^{-3}	1.84/−0.79	0.96/−1.04
Identification code	**Sm1**	**Eu1**
Empirical formula	$C_{18}H_{25}N_{15}O_{6.5}Sm$	$C_{18}H_{25}EuN_{15}O_{6.5}$
Formula weight/g·mol^{-1}	705.88	707.49
Temperature/K	100	100
Crystal system	monoclinic	monoclinic
Space group	$P2_1/n$	$P2_1/n$
a/Å	9.0379(6)	9.0203(5)
b/Å	17.6730(12)	17.6733(10)
c/Å	16.7718(11)	16.7461(10)
α/°	90	90
β/°	99.746(2)	99.712(2)
γ/°	90	90
Volume/Å^3	2640.2(3)	2631.4(3)
Z	4	4
ρ_{calc}g/cm^3	1.776	1.786
μ/mm^{-1}	2.292	2.452
F(000)	1408.0	1412.0
Crystal size/mm^3	0.346 × 0.302 × 0.15	0.219 × 0.176 × 0.133
Radiation	Mo K_α (λ = 0.71073 Å)	Mo K_α (λ = 0.71073 Å)
2θ range/°	4.61 to 61.038	4.61 to 61.064
Index ranges	$-12 \le h \le 12$, $-25 \le k \le 25$, $-23 \le l \le 23$	$-12 \le h \le 12$, $-25 \le k \le 25$, $-23 \le l \le 23$

Reflections collected	66683	82712
Independent reflections	8058 [R_{int} = 0.0245, R_{sigma} = 0.0129]	8023 [R_{int} = 0.0281, R_{sigma} = 0.0134]
Data/restraints/parameters	8058/3/468	8023/0/465
Goodness-of-fit on F^2	1.230	1.157
Final R indexes [I≥2σ (I)]	R_1 = 0.0180, wR_2 = 0.0402	R_1 = 0.0177, wR_2 = 0.0403
Final R indexes [all data]	R_1 = 0.0184, wR_2 = 0.0403	R_1 = 0.0182, wR_2 = 0.0405
Largest diff. peak/hole/e $Å^{-3}$	0.79/−0.86	1.09/−0.79
Identification code	**Gd1**	**Tb1**
Empirical formula	$C_{18}H_{25}GdN_{15}O_{6.5}$	$C_{18}H_{25}N_{15}O_{6.5}Tb$
Formula weight/g·mol^{-1}	712.78	714.45
Temperature/K	100.00	100
Crystal system	monoclinic	monoclinic
Space group	$P2_1/n$	$P2_1/n$
a/Å	9.0101(3)	8.9834(4)
b/Å	17.6704(7)	17.6574(8)
c/Å	16.7222(6)	16.7132(8)
α/°	90	90
β/°	99.7200(10)	99.788(2)
γ/°	90	90
Volume/Å^3	2624.16(17)	2612.5(2)
Z	4	4
ρ_{calc}g/cm^3	1.804	1.816
μ/mm^{-1}	2.596	2.776
F(000)	1416.0	1420.0
Crystal size/mm^3	0.268 × 0.201 × 0.187	0.404 × 0.145 × 0.105
Radiation	Mo K_α (λ = 0.71073 Å)	Mo K_α (λ = 0.71073 Å)
2θ range/°	4.942 to 61.09	4.946 to 61.074
Index ranges	$-12 \le h \le 12$, $-25 \le k \le 25$, $-23 \le l \le 23$	$-12 \le h \le 12$, $-25 \le k \le 25$, $-23 \le l \le 23$
Reflections collected	81875	92282
Independent reflections	8001 [R_{int} = 0.0237, R_{sigma} = 0.0111]	7984 [R_{int} = 0.0278, R_{sigma} = 0.0126]
Data/restraints/parameters	8001/0/460	7984/0/388
Goodness-of-fit on F^2	1.212	1.171
Final R indexes [I≥2σ (I)]	R_1 = 0.0171, wR_2 = 0.0376	R_1 = 0.0168, wR_2 = 0.0391
Final R indexes [all data]	R_1 = 0.0175, wR_2 = 0.0377	R_1 = 0.0171, wR_2 = 0.0393
Largest diff. peak/hole/e $Å^{-3}$	1.27/−0.78	0.68/−0.85
Identification code	**Dy1**	**Ho1**
Empirical formula	$C_{18}H_{25}DyN_{15}O_{6.5}$	$C_{18}H_{24}HoN_{15}O_6$
Formula weight/g·mol^{-1}	718.03	711.45

Temperature/K	100	100
Crystal system	monoclinic	monoclinic
Space group	$P2_1/n$	$P2_1/n$
a/Å	8.9635(3)	8.9232(6)
b/Å	17.6526(6)	17.8024(10)
c/Å	16.6753(6)	16.7130(11)
α/°	90	90
β/°	99.7540(10)	100.108(2)
γ/°	90	90
Volume/Å^3	2600.38(16)	2613.7(3)
Z	4	4
ρ_{calc}g/cm^3	1.834	1.808
μ/mm^{-1}	2.942	3.093
F(000)	1424.0	1408.0
Crystal size/mm^3	0.296 × 0.16 × 0.113	0.272 × 0.173 × 0.09
Radiation	Mo K_α (λ = 0.71073 Å)	Mo K_α (λ = 0.71073 Å
2θ range/°	4.852 to 61.124	4.858 to 57.396
Index ranges	$-12 \le h \le 12$, $-25 \le k \le 25$, $-23 \le l \le 23$	$-10 \le h \le 12$, $-24 \le k \le 24$, $-22 \le l \le 22$
Reflections collected	76126	67639
Independent reflections	7956 [R_{int} = 0.0275, R_{sigma} = 0.0158]	6730 [R_{int} = 0.0613, R_{sigma} = 0.0299]
Data/restraints/parameters	7956/0/388	6730/3/377
Goodness-of-fit on F^2	1.133	1.232
Final R indexes [I≥2σ (I)]	R_1 = 0.0164, wR_2 = 0.0385	R_1 = 0.0435, wR_2 = 0.0905
Final R indexes [all data]	R_1 = 0.0167, wR_2 = 0.0386	R_1 = 0.0463, wR_2 = 0.0915
Largest diff. peak/hole/e Å^{-3}	0.79/−0.75	2.01/−2.58

R_1, = $\sum||F_c|-|F_c|/\sum|F_o|$. $\omega R_2 = [\sum\{\omega(F_o^2-F_c^2)^2/\sum\omega(F_o^2)^2\}^{1/2}$

Table S2.2. Bond Lengths for **Ln1**.

Bonds	Length/Å				
	**La1**	**Ce1**	**Pr1**	**Nd1**	**Sm1**
Ln1–O2	2.5226(14)	2.4980(17)	2.4822(14)	2.4617(12)	2.4318(11)
Ln1–O3	2.5386(14)	2.5089(18)	2.4915(15)	2.4722(13)	2.4436(12)
Ln1–O1	2.4856(14)	2.4563(18)	2.4392(15)	2.4253(12)	2.3948(11)
Ln1–N6	2.7391(17)	2.712(2)	2.7003(18)	2.6811(14)	2.6568(13)
Ln1–N1	2.7606(17)	2.738(2)	2.7242(18)	2.7095(14)	2.6940(13)
Ln1–N11	2.7573(16)	2.734(2)	2.7184(17)	2.7011(14)	2.6791(13)
Ln1–N2	2.6587(16)	2.634(2)	2.6162(17)	2.5962(14)	2.5683(13)
Ln1–N7	2.6484(17)	2.620(2)	2.6079(17)	2.5868(14)	2.5604(13)
Ln1–N12	2.6704(16)	2.647(2)	2.6324(17)	2.6155(14)	2.5930(13)
Bonds	Length/Å				
	**Eu1**	**Gd1**	**Tb1**	**Dy1**	**Ho1**
Ln1–O2	2.4199(11)	2.4097(11)	2.3987(10)	2.3847(10)	2.367(3)
Ln1–O3	2.4302(12)	2.4171(11)	2.4036(11)	2.3849(11)	2.357(4)
Ln1–O1	2.3859(11)	2.3767(11)	2.3568(11)	2.3450(10)	2.345(4)
Ln1–N6	2.6454(13)	2.6362(13)	2.6235(12)	2.6125(12)	2.611(4)
Ln1–N1	2.6830(13)	2.6758(13)	2.6703(13)	2.6593(12)	2.655(4)
Ln1–N11	2.6656(12)	2.6544(12)	2.6444(12)	2.6298(12)	2.616(4)
Ln1–N2	2.5553(13)	2.5445(12)	2.5309(13)	2.5188(12)	2.502(4)
Ln1–N7	2.5491(13)	2.5404(12)	2.5255(12)	2.5093(12)	2.497(4)
Ln1–N12	2.5832(12)	2.5734(12)	2.5626(12)	2.5517(12)	2.541(4)

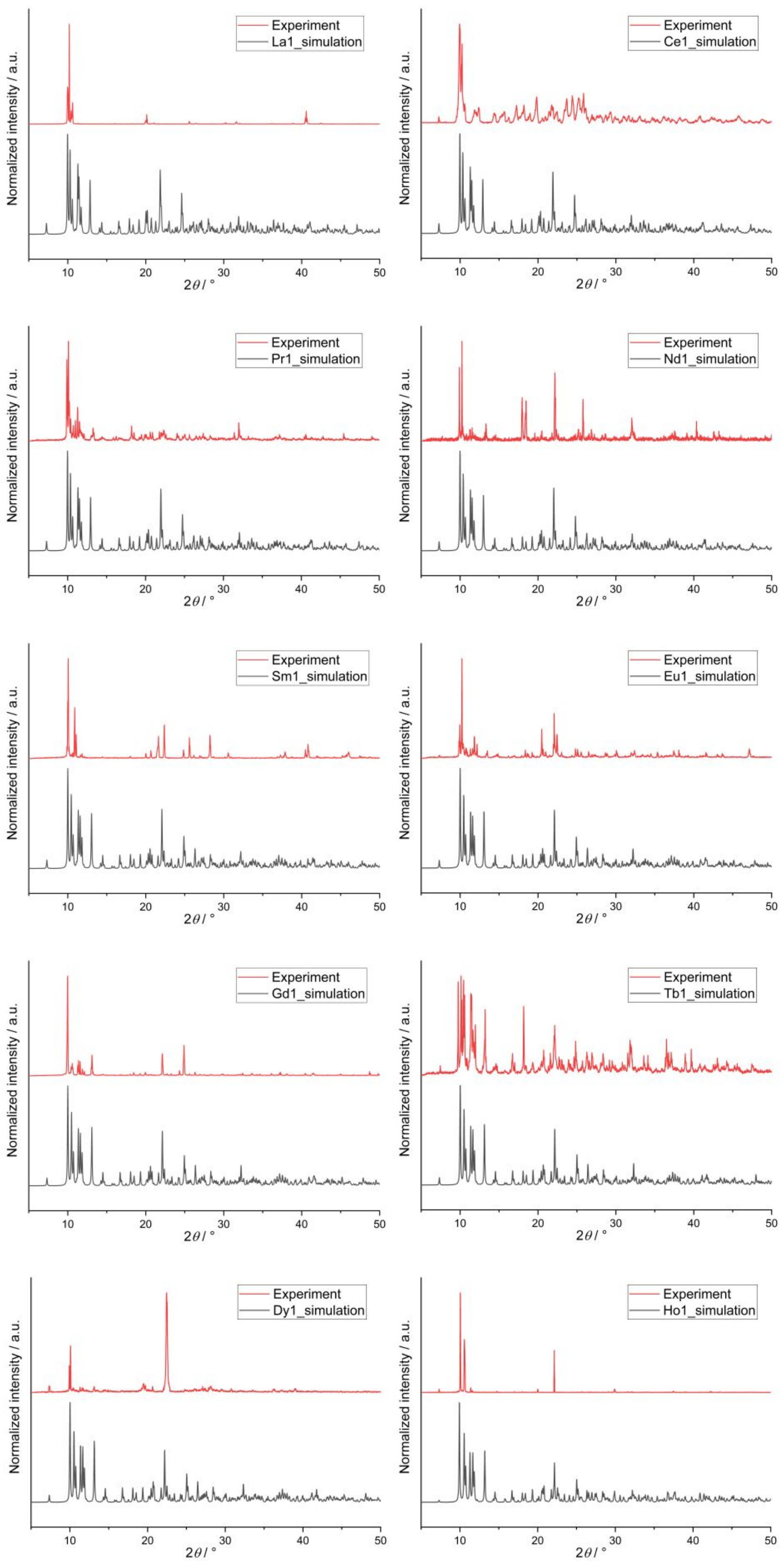

Figure S2.1. Simulated (**Ln1**) and experimental PXRD pattern of the isolated Ln product.

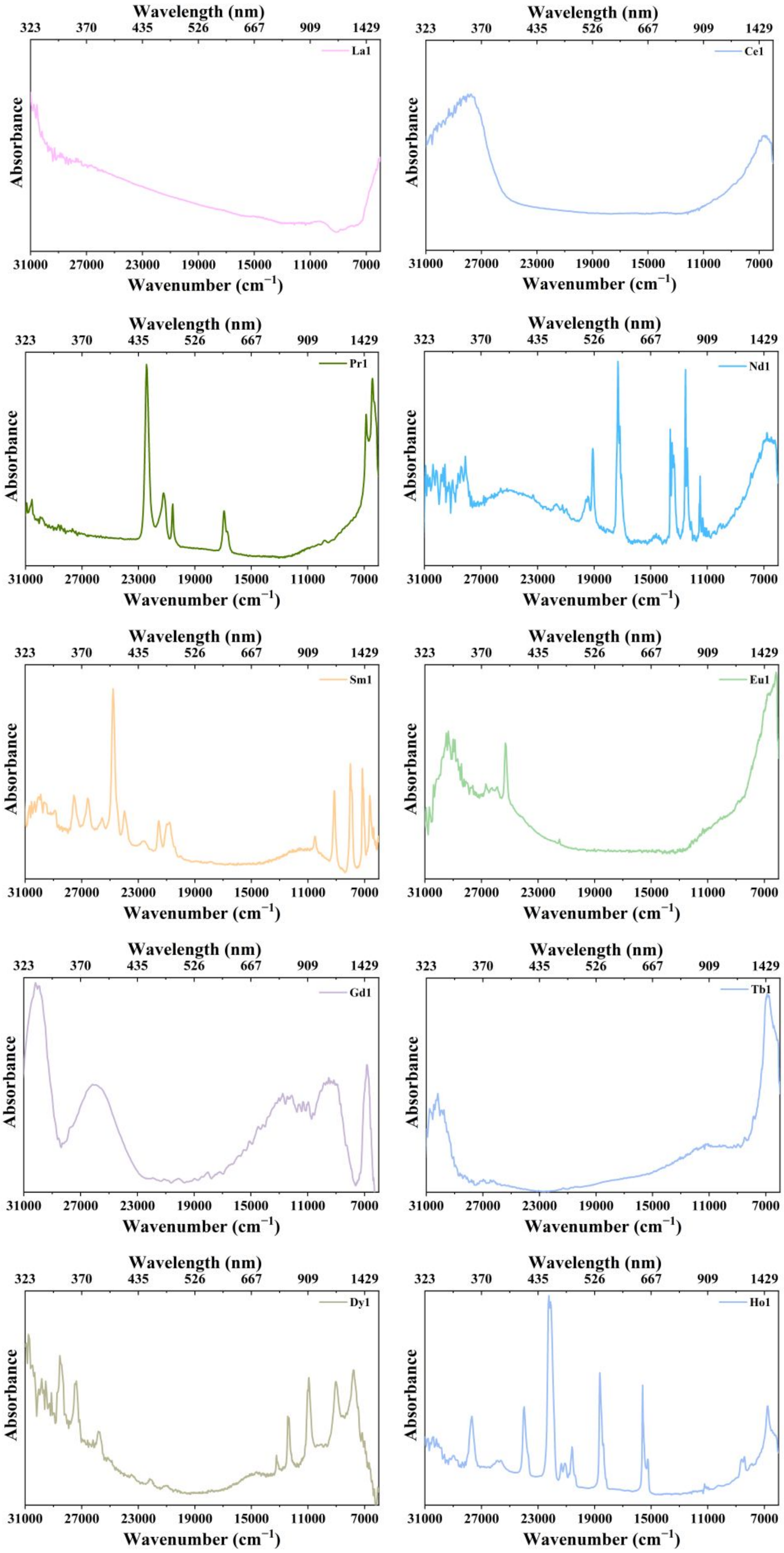

Figure S2.2. Solid-state UV-vis-NIR absorption spectrum of **Ln1**.

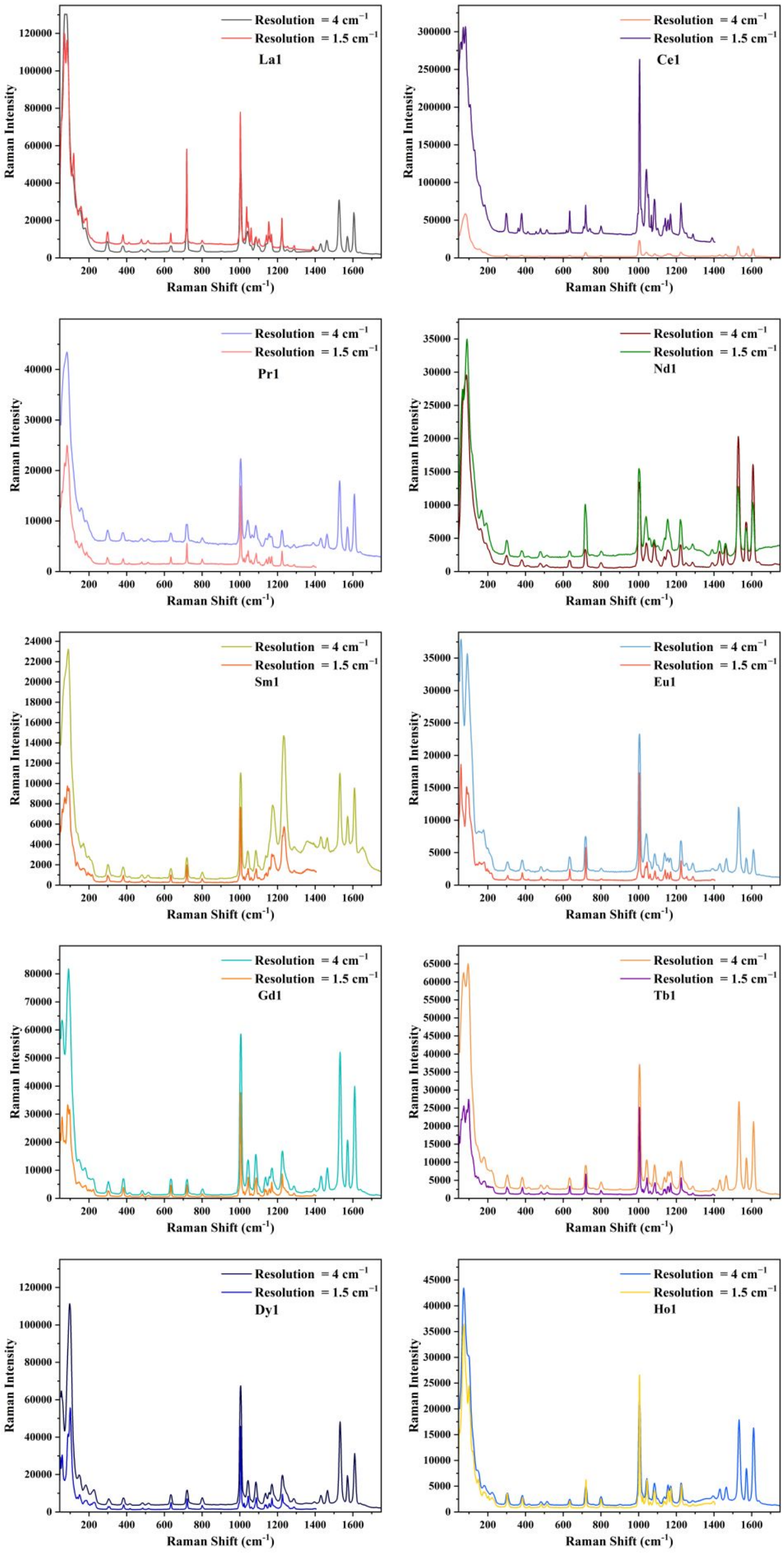

Figure S2.3. Raman spectrum of **Ln1** acquired from single crystals.

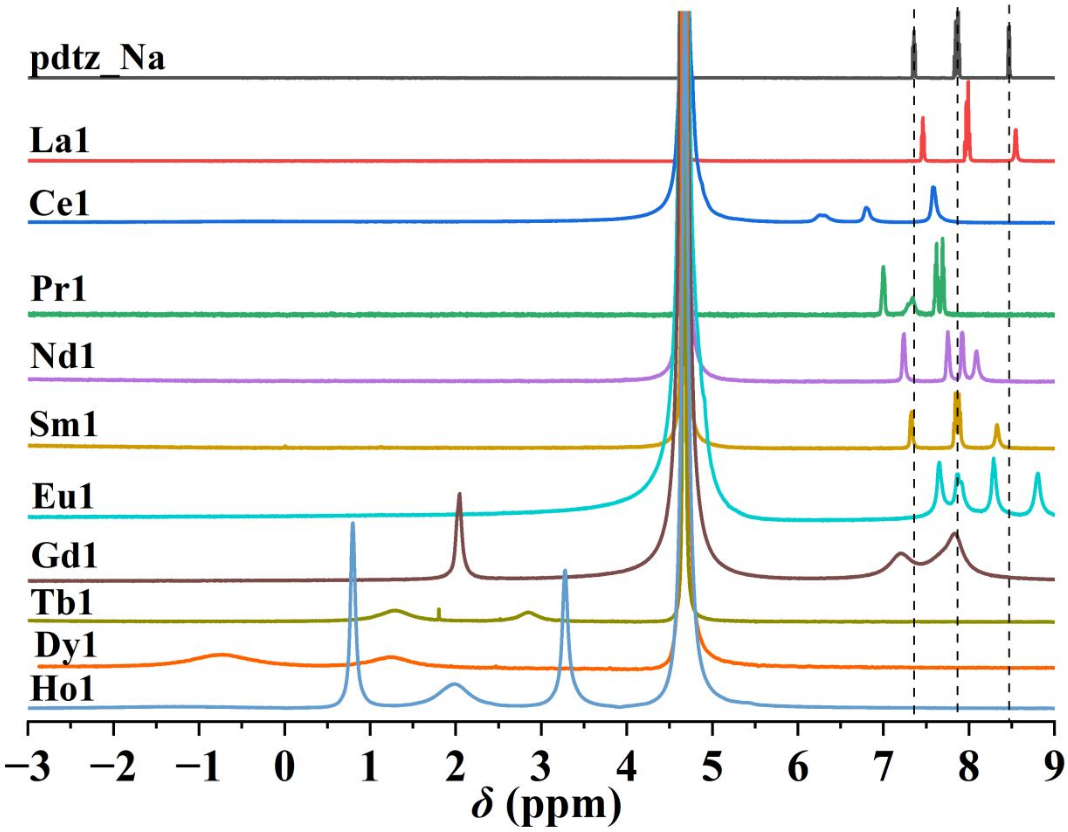

Figure S2.4. ^{1}H NMR spectrum of **Ln1** acquired in D_2O solution.

Section S3: Crystallographic data for **Pu1**, **Am1** and **Cm1**

Identification code	**Pu1**	**Am1**	**Cm1**
Empirical formula	$C_{18}H_{24.98}N_{15}O_{6.5}Pu$	$C_{18}H_{24.97}AmN_{15}O_{6.5}$	$C_{18}H_{24.94}CmN_{15}O_{6.5}$
Formula weight/g·mol^{-1}	797.53	798.53	803.53
Temperature/K	100.00	100.00	100.00
Crystal system	monoclinic	monoclinic	monoclinic
Space group	$P2_1/n$	$P2_1/n$	$P2_1/n$
a/Å	9.0816(3)	9.0692(9)	9.0763(11)
b/Å	17.7123(5)	17.7014(18)	17.714(2)
c/Å	16.8223(5)	16.7768(17)	16.7778(19)
α/°	90	90	90
β/°	99.8420(10)	99.658(3)	99.686(4)
γ/°	90	90	90
Volume/Å^3	2666.14(14)	2655.1(5)	2659.0(5)
Z	4	4	4
ρ_{calc}g/cm^3	1.987	1.998	2.007
μ/mm^{-1}	2.540	2.957	3.133
F(000)	1536.0	1540.0	1544.0
Crystal size/mm^3	0.1 × 0.1 × 0.1	0.264 × 0.154 × 0.14	0.164 × 0.125 × 0.08
Radiation	Mo K_α (λ = 0.71073 Å)	Mo K_α (λ = 0.71073 Å)	Mo K_α (λ = 0.71073 Å)
2θ range/°	4.916 to 54.958	4.926 to 61.146	4.798 to 61.148
Index ranges	$-11 \le h \le 11$, $-23 \le k \le 21$, $-20 \le l \le 21$	$-12 \le h \le 12$, $-25 \le k \le 25$, $-24 \le l \le 23$	$-12 \le h \le 12$, $-25 \le k \le 25$, $-24 \le l \le 23$
Reflections collected	18407	76303	74702
Independent reflections	6084 [R_{int} = 0.0307, R_{sigma} = 0.0347]	8128 [R_{int} = 0.0389, R_{sigma} = 0.0183]	8128 [R_{int} = 0.0529, R_{sigma} = 0.0249]
Data/restraints/parameters	6084/0/453	8128/7/399	8128/0/445
Goodness-of-fit on F^2	1.266	1.278	1.190
Final R indexes [I≥2σ (I)]	R_1 = 0.0361, wR_2 = 0.0590	R_1 = 0.0184, ωR_2 = 0.0423	R_1 = 0.0238, ωR_2 = 0.0538
Final R indexes [all data]	R_1 = 0.0422, wR_2 = 0.0604	R_1 = 0.0188, ωR_2 = 0.0425	R_1 = 0.0250, ωR_2 = 0.0543
Largest diff. peak/hole/e Å^{-3}	1.99/−3.17	1.01/−1.20	2.26/−1.59

R_1, = $\sum||F_c|-|F_c|/\sum|F_o|$.

$\omega R_2 = [\sum\{\omega(F_o^2-F_c^2)^2/\sum\omega(F_o^2)^2\}^{1/2}$

Table S3.2. Bond Lengths (Å) for **Pu1**, **Am1** and **Cm1**.

Bonds	**Pu1**	**Am1**	**Cm1**
An11–O2	2.482(3)	2.4701(14)	2.465(2)
An11–O3	2.483(4)	2.4796(15)	2.471(2)
An11–O1	2.427(4)	2.4280(15)	2.4236(19)
An11–N6	2.676(4)	2.6677(17)	2.661(2)
An11–N1	2.706(4)	2.6958(18)	2.693(2)
An11–N11	2.705(4)	2.6852(17)	2.677(2)
An11–N2	2.591(4)	2.5782(17)	2.570(2)
An11–N7	2.585(3)	2.5711(18)	2.569(2)
An11–N12	2.611(4)	2.5995(17)	2.594(2)

Table S3.3. Hydrogen Bonds for **Pu1**.

D	H	A	d(D-H)/Å	d(H-A)/Å	d(D-A)/Å	D-H-A/°
O6	H6A	O7	0.87	1.89	2.737(10)	163.1
O6	H6A	$O7^{1}$	0.87	2.06	2.783(10)	139.3
O6	H6B	$N3^{2}$	0.87	2.07	2.893(6)	156.8
O2	H2A	$N9^{3}$	0.87(6)	1.86(6)	2.719(5)	173(5)
O4	H4A	$N4^{4}$	0.80(6)	2.06(6)	2.830(6)	164(5)
O5	H5A	$N14^{5}$	0.75(6)	2.16(6)	2.887(5)	165(6)
O3	H3B	O6	0.74(6)	2.04(6)	2.766(6)	169(6)
O5	H5B	$N15^{6}$	0.84(7)	1.98(7)	2.822(5)	178(6)
O2	H2B	$N5^{4}$	0.86(7)	1.96(7)	2.797(5)	166(6)
O1	H1A	O4	0.72(6)	2.01(6)	2.716(5)	164(6)
O1	H1B	O5	0.80(6)	1.84(6)	2.629(5)	165(6)

$^{1}2-x, 1-y, 2-z$; $^{2}-1/2+x, 1/2-y, -1/2+z$; $^{3}1/2+x, 1/2-y, 1/2+z$; $^{4}2-x, 1-y, 1-z$; $^{5}1-x, 1-y, 1-z$; $^{6}1+x, +y, +z$.

Table S3.4. Hydrogen Bonds for **Am1**.

D	H	A	d(D-H)/Å	d(H-A)/Å	d(D-A)/Å	D-H-A/°
O5	H5A	N15	0.87	1.95	2.819(2)	176.5
O5	H5B	$N14^{1}$	0.87	2.03	2.886(3)	168.7
O1	H1A	$O5^{2}$	0.87	1.77	2.633(2)	173.4
O1	H1B	$O4^{3}$	0.87	1.87	2.719(2)	163.5
O6	H6A	O7	0.87	1.94	2.744(5)	152.3
O6	H6A	$O7^{4}$	0.87	2.00	2.792(5)	150.1
O6	H6B	$N3^{5}$	0.87	2.09	2.897(3)	154.8
O2	H2A	$N9^{6}$	0.75(4)	1.97(4)	2.722(2)	177(4)
O4	H4A	N4	0.77(4)	2.07(4)	2.831(3)	173(4)
O2	H2B	$N5^{3}$	0.86(4)	1.98(4)	2.804(2)	162(4)

$^{1}2-x, 1-y, 2-z$; $^{2}-1/2+x, 1/2-y, -1/2+z$; $^{3}1/2+x, 1/2-y, 1/2+z$; $^{4}2-x, 1-y, 1-z$; $^{5}1-x, 1-y, 1-z$; $^{6}1+x, +y, +z$.

Table S3.5. Hydrogen Bonds for **Cm1**.

D	H	A	d(D-H)/Å	d(H-A)/Å	d(D-A)/Å	D-H-A/°
O5	H5A	N15	0.87	1.95	2.823(3)	175.1
O5	H5B	N14[1]	0.87	2.03	2.889(3)	169.0
O3	H3A	O6[2]	0.88	2.18	2.751(4)	122.5
O1	H1A	O5[3]	0.87	1.77	2.634(3)	169.4
O1	H1B	O4[4]	0.87	1.86	2.720(3)	167.8
O6	H6A	O7	0.87	1.94	2.797(7)	165.8
O6	H6A	O7[5]	0.87	2.07	2.754(7)	135.3
O2	H2A	N9[6]	0.75(5)	1.98(5)	2.729(3)	175(5)
O4	H4A	N4[2]	0.73(5)	2.10(5)	2.834(3)	174(5)
O2	H2B	N5[7]	0.89(5)	1.97(5)	2.810(3)	159(4)

[1]2−*x*, 1−*y*, 2−*z*; [2]−1/2+*x*, 1/2−*y*, −1/2+*z*; [3]1/2+*x*, 1/2−*y*, 1/2+*z*; [4]2−*x*, 1−*y*, 1−*z*; [5]1−*x*, 1−*y*, 1−*z*; [6]1+*x*, +*y*, +*z*.

Section S4: Additional crystal structures

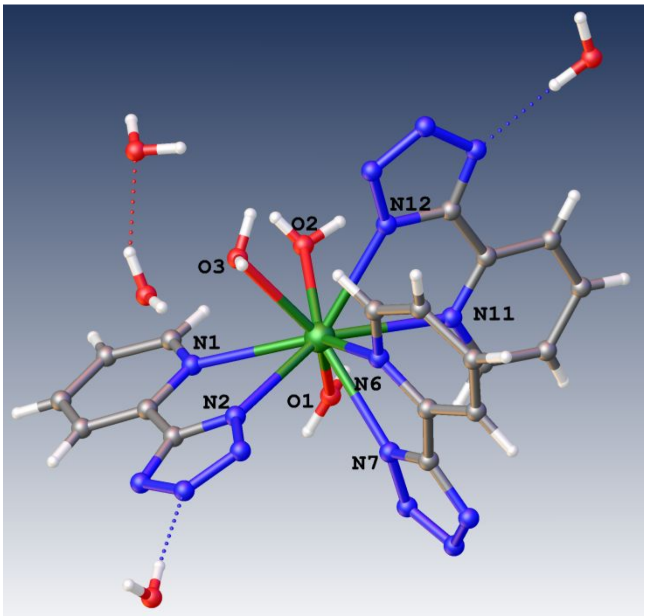

Figure S4.1. Molecular structure of $[M(pdtz)_3(H_2O)_3]\cdot 3.5H_2O$ (M = La – Nd, Sm – Ho, and Pu – Cm, green; N, blue; O, red; C, gray; H, white) with coordinated with metal center atoms label from Olex-2. The atom labels correspond to atom names discussed.

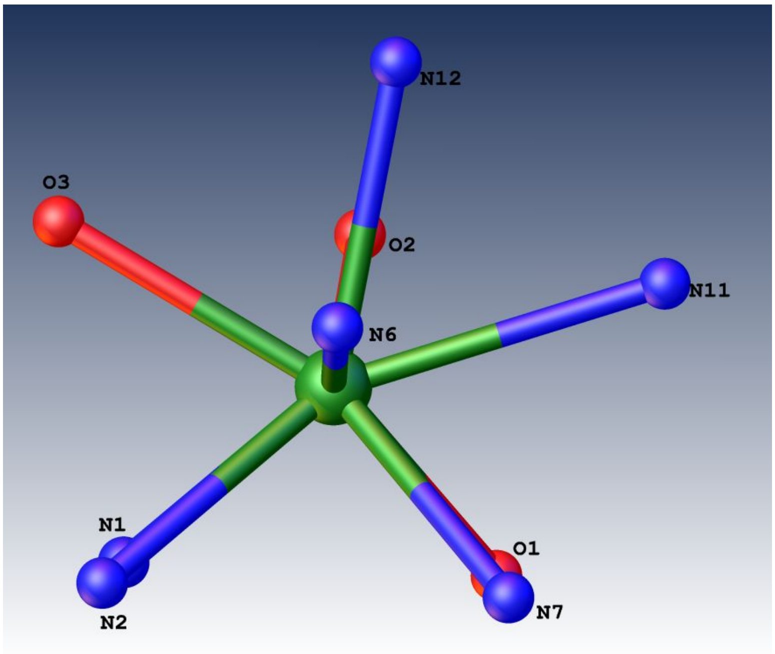

Figure S4.2. Figure of the bonding atoms showing the capped square antiprism coordination environment.

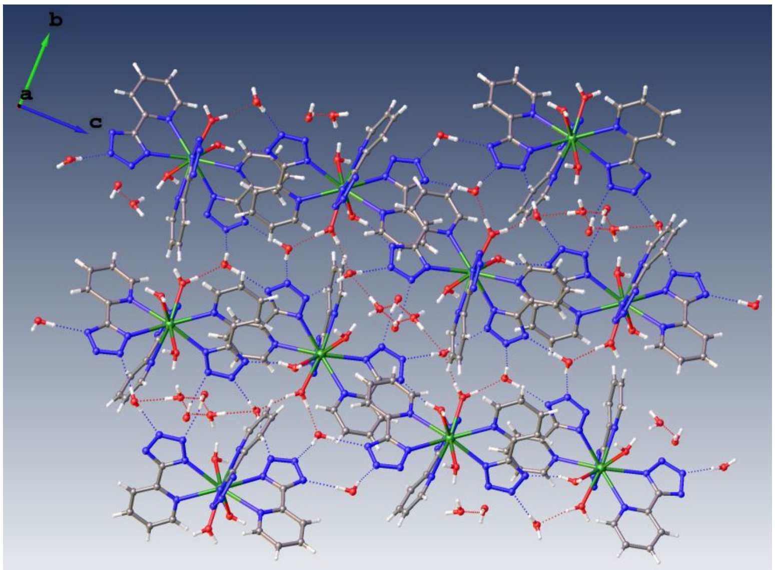

Figure S4.3. The packing structure viewed along the *a*-axis, of $[M(pdtz)_3(H_2O)_3]\cdot 3.5H_2O$ (M = La – Nd, Sm – Ho, and Pu – Cm, light green; N, light blue; O, red; C, gray; H, white).

Section S5: SHAPE results

Table S5.1 The deviation results of $[M(pdtz)_3(H_2O)_3]\cdot 3.5H_2O$ (M = La – Nd, Sm – Ho, and Pu – Cm) from ideal capped square antiprism and muffin from the shape program.[15]

Compounds	Capped square antiprism	Muffin
**La1**	0.822	1.014
**Ce1**	0.775	0.978
**Pr1**	0.745	0.975
**Nd1**	0.743	0.927
**Sm1**	0.690	0.943
**Eu1**	0.670	0.925
**Gd1**	0.657	0.919
**Tb1**	0.658	0.919
**Dy1**	0.644	0.904
**Ho1**	0.639	0.880
**Pu1**	0.680	0.941
**Am1**	0.659	0.918
**Cm1**	0.641	0.899

Section S6: Additional bond length analysis

Table S6.1 Bond lengths for **Ce1**, **Pr1**, **Nd1**, **Sm1**, **Pu1**, **Am1**, and **Cm1**. For a clearer and more direct comparison of the bond lengths of **Ce1**, **Pr1**, **Nd1**. **Sm1**. **Pu1**. **Am1**, and **Cm1**, the summary table is presented again. The average bond lengths are shown in bold.

	**Compounds**			
Bond (Å)	**Pu1**	**Ce1**	**Pr1**	
M1–O2	2.482(3)	2.4980(17)	2.4822(14)	
M1–O3	2.483(4)	2.5089(18)	2.4915(15)	
M1–O1	2.427(4)	2.4563(18)	2.4392(15)	
Average M–O	**2.464**	**2.4877**	**2.4715**	
M1–N6	2.676(4)	2.712(2)	2.7003(18)	
M1–N1	2.706(4)	2.738(2)	2.7242(18)	
M1–N11	2.705(4)	2.734(2)	2.7184(17)	
Average M–$N_{pyridine}$	**2.696**	**2.7280**	**2.7144**	
M1–N2	2.591(4)	2.634(2)	2.6162(17)	
M1–N7	2.585(3)	2.620(2)	2.6079(17)	
M1–N12	2.611(4)	2.647(2)	2.6324(17)	
Average M–$N_{tetrazole}$	**2.596**	**2.634**	**2.6191**	
	**Compounds**			
Bond (Å)	**Nd1**	**Am1**	**Cm1**	**Sm1**
M1–O2	2.4617(12)	2.4701(14)	2.465(2)	2.4318(11)
M1–O3	2.4722(13)	2.4796(15)	2.471(2)	2.4436(12)
M1–O1	2.4253(12)	2.4280(15)	2.4236(19)	2.3948(11)
Average M–O	**2.4531**	**2.4594**	**2.453**	**2.4235**
M1–N6	2.6811(14)	2.6677(17)	2.661(2)	2.6568(13)
M1–N1	2.7095(14)	2.6958(18)	2.693(2)	2.6940(13)
M1–N11	2.7011(14)	2.6852(17)	2.677(2)	2.6791(13)
Average M–$N_{pyridine}$	**2.6973**	**2.6831**	**2.677**	**2.6766**
M1–N2	2.5962(14)	2.5782(17)	2.570(2)	2.5683(13)
M1–N7	2.5868(14)	2.5711(18)	2.569(2)	2.5604(13)
M1–N12	2.6155(14	2.5995(17)	2.594(2)	2.5930(13)
Average M–$N_{tetrazole}$	**2.5994**	**2.5829**	**2.578**	**2.5738**

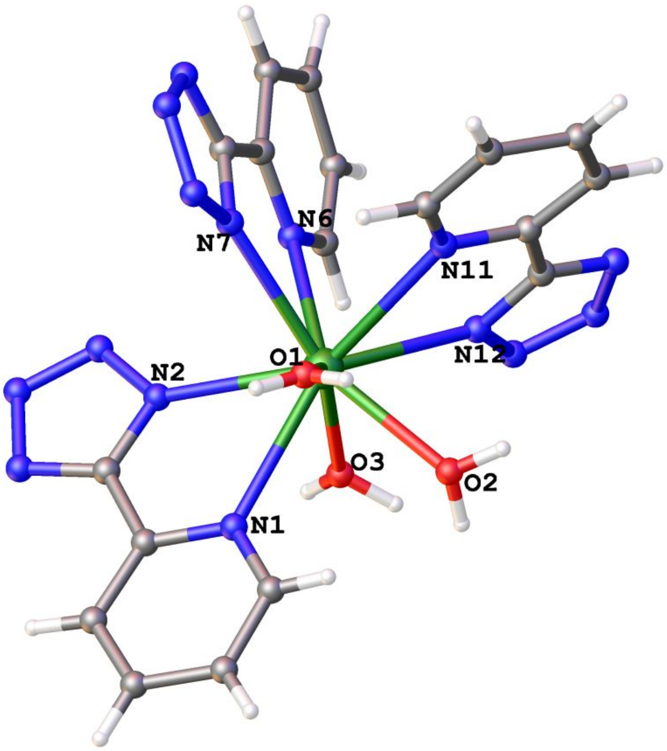

(a)

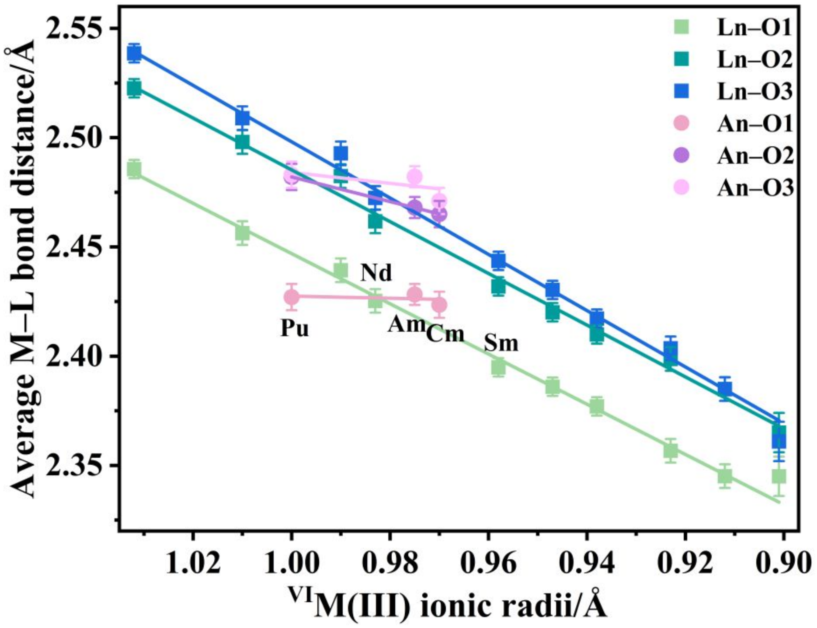

(b)

Figure S6.1. (a). Molecular structure of $[M(pdtz)_3(H_2O)_3]$ (M = La – Nd, Sm – Ho, and Pu – Cm, green; N, blue; O, red; C, gray; H, white) with coordinated with metal center atoms label from Olex-2. The atom labels correspond to atom names discussed in this section. (b) The M–H_2O bond length (Å) of isostructural *f*-element-pdtz, $[M(pdtz)_3(H_2O)_3]\cdot 3.5H_2O$ with trend lines.

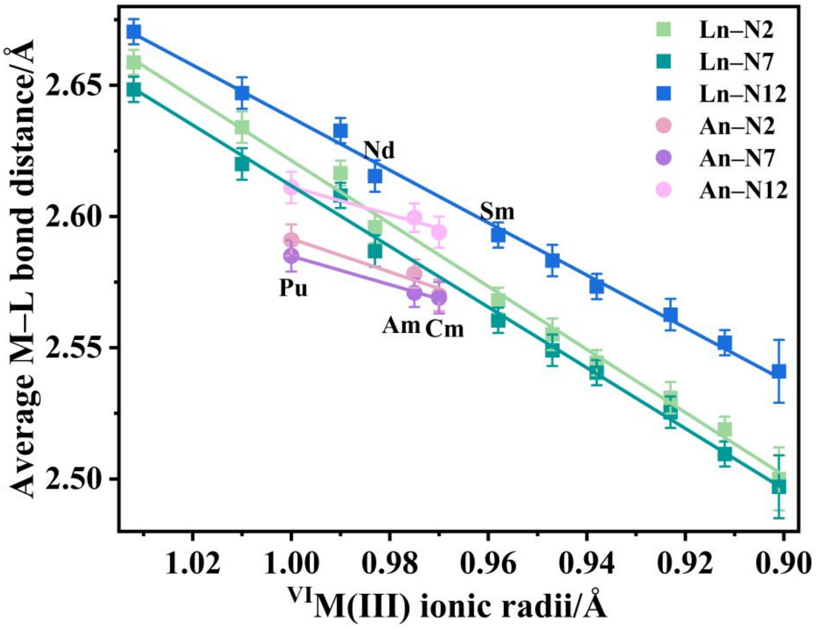

Figure S6.2. The M–N$_{tetrazolate}$ bond length (Å) of isostructural *f*-element-pdtz, $[M(pdtz)_3(H_2O)_3]\cdot 3.5H_2O$ with trend lines.

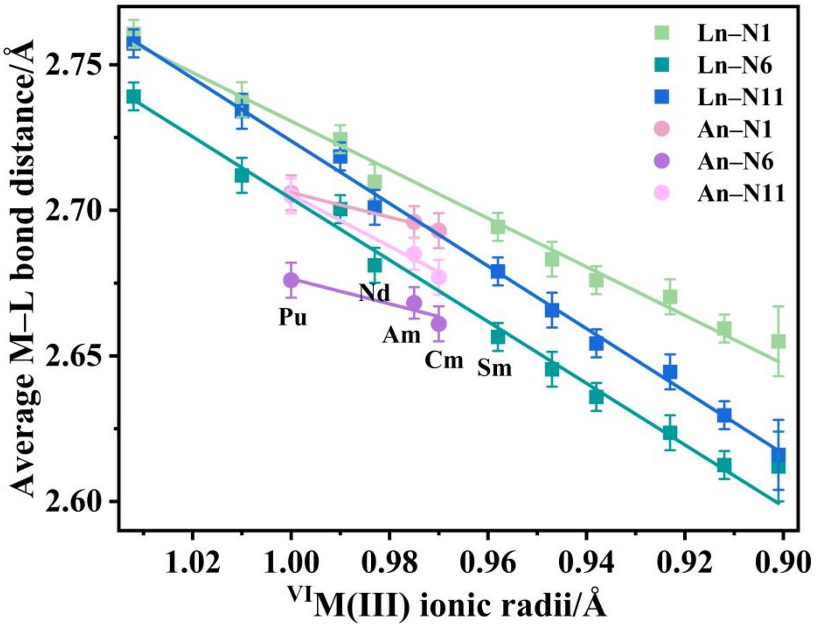

Figure S6.3. The M–N$_{pyridyl}$ bond length (Å) of isostructural *f*-element-pdtz, $[M(pdtz)_3(H_2O)_3]\cdot 3.5H_2O$ with trend lines.

Section S7: The quantum theory of atoms in molecules (QTAIM) and natural localized molecular orbital (NLMO)

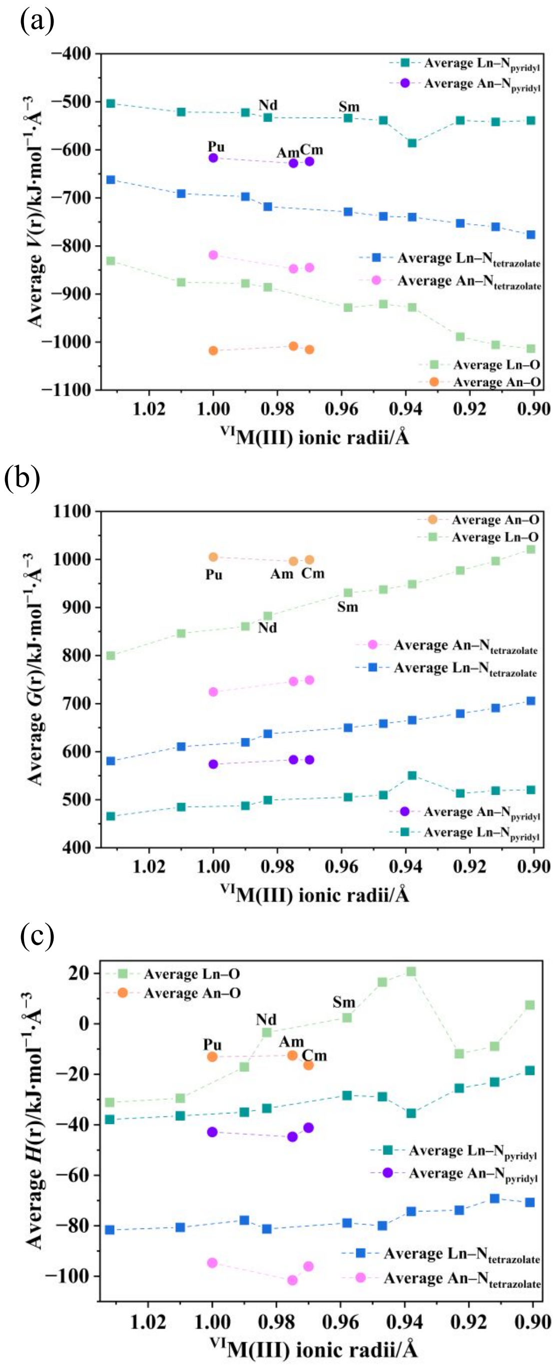

Figure S7.1. QTAIM metrics of M–H_2O and M–$N_{tetrazolate}$ and M–$N_{pyridyl}$ bonds in $[M(pdtz)_3(H_2O)_3]\cdot 3.5H_2O$, including (a) potential (V), (b) kinetic (G), and (c) total (H) energy densities in kJ·mol^{-1}·Å^{-3}, at the bond critical points (BCPs).[16]

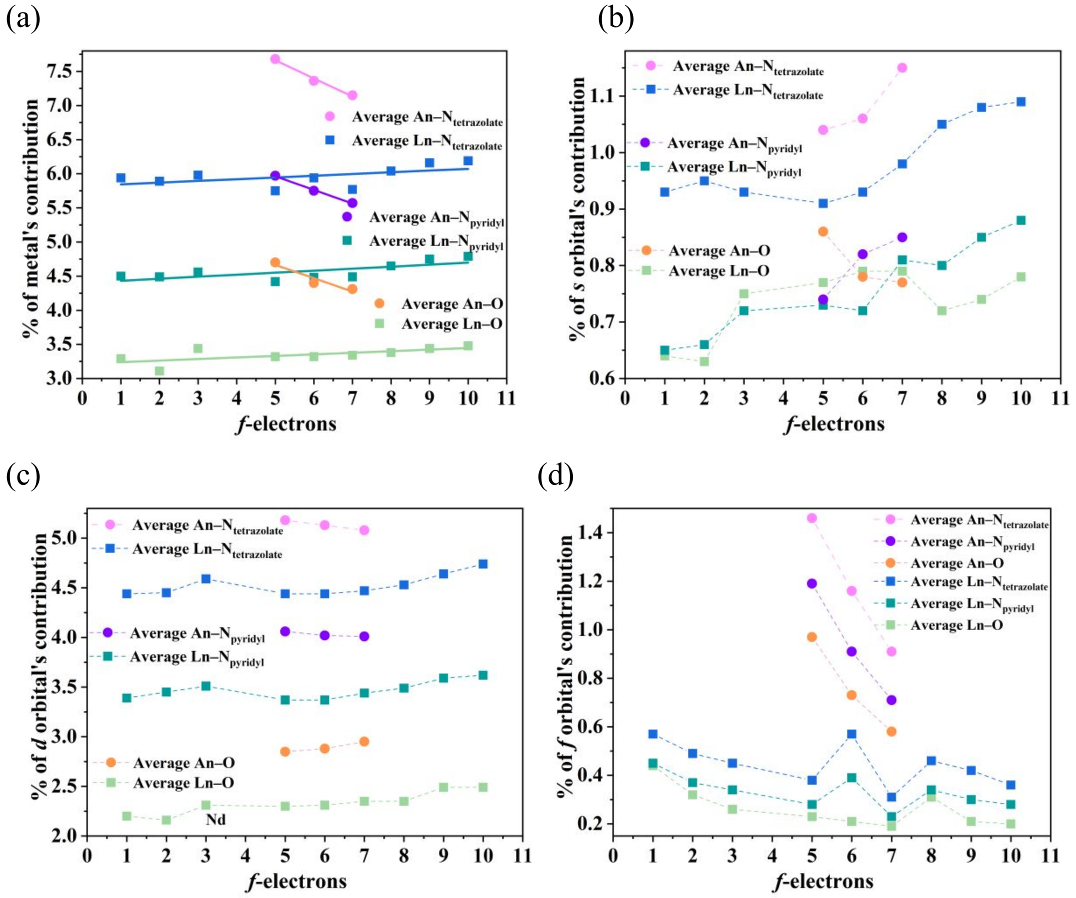

Figure S7.2. β spin of composition (a) Total metal contribution (b) 6*s*/7*s* orbital contribution, (c) 5*d*/6*d* orbital contribution (d) 4*f*/5*f* orbital contribution of M–O and $M–N_{tetrazolate}$ and $M–N_{pyridyl}$ bonds.

La1

La−L	*ρ(r)*	*δ(r)*	*V(r)*	*G(r)*	*H(r)*	*|V(r)|/G(r)*	*H(r)/ρ(r)*	*λ(Nd)*	*OS(M)*
La1−O1	0.3022	0.2281	−828.2676	794.3764	−33.8911	1.042664	−112.139	53.6086	3.4
La1−O2	0.2928	0.2322	−784.7371	751.7933	−32.9438	1.04382	−112.525		
La1−O3	0.3115	0.2315	−880.2080	853.7749	−26.4331	1.03096	−84.8617		
**Average La−O**	**0.3022**	**0.2306**	**−831.0709**	**799.9815**	**−31.0893**	**1.038863**	**−102.891**		
La1−N4	0.2349	0.2137	−519.8848	473.1136	−46.7710	1.098858	−199.072		
La1−N5	0.2248	0.2012	−493.2327	460.0103	−33.2224	1.072221	−147.788		
La1−N6	0.2258	0.1984	−496.9506	463.3401	−33.6105	1.07254	−148.819		
**Average La−$N_{pyridyl}$**	**0.2285**	**0.204433**	**−503.3560**	**465.4880**	**−37.8680**	**1.081351**	**−165.702**		
La1−N7	0.2771	0.2532	−662.5926	578.9471	−83.6455	1.144479	−301.815		
La1−N8	0.2850	0.2633	−687.6639	593.4327	−94.2312	1.15879	−330.621		
La1−N9	0.2675	0.2349	−636.5570	569.5287	−67.0284	1.117691	−250.55		
**Average La−$N_{tetrazolate}$**	**0.2766**	**0.250467**	**−662.2712**	**580.6362**	**−81.6350**	**1.140596**	**−295.18**		

Ce1

Ce−L	*ρ(r)*	*δ(r)*	*V(r)*	*G(r)*	*H(r)*	*|V(r)|/G(r)*	*H(r)/ρ(r)*	*λ(Ce)*	*OS(M)*
Ce1−O1	0.3079	0.2347	−860.1210	831.0783	−29.0427	1.034946	−94.3349	54.6004	3.4
Ce1−O2	0.3036	0.2378	−832.7463	796.8812	−35.8649	1.045007	−118.141		
Ce1−O3	0.3218	0.2403	−933.9506	910.4475	−23.5031	1.025815	−73.0263		
**Average Ce−O**	**0.3111**	**0.2376**	**−875.6060**	**846.1357**	**−29.4702**	**1.034829**	**−94.7301**		
Ce1−N4	0.2408	0.219	−542.8805	495.3880	−47.4924	1.095869	−197.23		
Ce1−N5	0.2275	0.2038	−507.5093	478.1417	−29.3675	1.06142	−129.112		
Ce1−N6	0.2297	0.2024	−513.3665	480.9198	−32.4467	1.067468	−141.244		
**Average Ce−$N_{pyridyl}$**	**0.2327**	**0.2084**	**−521.2521**	**484.8165**	**−36.4355**	**1.075153**	**−156.605**		
Ce1−N7	0.2829	0.2595	−689.4028	606.3290	−83.0737	1.137011	−293.601		
Ce1−N8	0.2933	0.2704	−724.7240	629.1032	−95.6208	1.151995	−325.971		
Ce1−N9	0.2718	0.239	−659.2689	596.0580	−63.2107	1.106048	−232.552		
**Average Ce−$N_{tetrazolate}$**	**0.2827**	**0.2563**	**−691.1319**	**610.4967**	**−80.6351**	**1.132081**	**−285.231**		

Pr1

Pr−L	*ρ(r)*	*δ(r)*	*V(r)*	*G(r)*	*H(r)*	*|V(r)|/G(r)*	*H(r)/ρ(r)*	*λ(Pr)*	*OS(M)*
Pr1−O1	0.3101	0.2324	−872.1696	844.3539	−27.8158	1.032943	−89.6965	55.6126	3.4
Pr1−O2	0.2940	0.2303	−813.8674	804.4142	−9.4534	1.011752	−32.1568		
Pr1−O3	0.3227	0.2388	−947.7006	933.7504	−13.9502	1.01494	−43.231		
**Average Pr−O**	**0.3089**	**0.233833**	**−877.9126**	**860.8395**	**−17.0731**	**1.019833**	**−55.2661**		
Pr1−N4	0.2405	0.2208	−541.3399	493.3651	−47.9749	1.09724	−199.448		
Pr1−N5	0.2260	0.2024	−507.3474	483.3434	−24.0040	1.049662	−106.189		
Pr1−N6	0.2311	0.2037	−518.1259	485.1197	−33.0063	1.068037	−142.846		
**Average Pr−$N_{pyridyl}$**	**0.2325**	**0.208967**	**−522.2711**	**487.2761**	**−34.9950**	**1.071818**	**−150.484**		
Pr1−N7	0.2850	0.2598	−700.8027	619.6676	−81.1350	1.130933	−284.662		
Pr1−N8	0.2936	0.2722	−727.5599	633.6673	−93.8926	1.148173	−319.821		
Pr1−N9	0.2717	0.2416	−663.5562	605.1767	−58.3794	1.096467	−214.875		
**Average Pr−$N_{tetrazolate}$**	**0.2834**	**0.257867**	**−697.3062**	**619.5039**	**−77.8024**	**1.125588**	**−274.503**		

Nd1

Nd−L	*ρ(r)*	*δ(r)*	*V(r)*	*G(r)*	*H(r)*	*|V(r)|/G(r)*	*H(r)/ρ(r)*	*λ(Nd)*	*OS(M)*
Nd1−O1	0.3051	0.2212	−872.4509	868.8095	−3.6413	1.004191	−11.9332	56.6245	3.4
Nd1−O2	0.2918	0.2162	−822.6471	832.2418	9.5948	0.988471	32.88437		
Nd1−O3	0.3261	0.2349	−962.6135	946.4321	−16.1815	1.017097	−49.6167		
**Average Nd−O**	**0.3077**	**0.2241**	**−885.9038**	**882.4945**	**−3.4093**	**1.003863**	**−11.0807**		
Nd1−N4	0.2428	0.2237	−555.3759	512.0152	−43.3608	1.084686	−178.559		
Nd1−N5	0.2277	0.2008	−512.3806	487.0594	−25.3211	1.051988	−111.22		
Nd1−N6	0.2338	0.2041	−530.3925	498.6391	−31.7533	1.06368	−135.801		
**Average Nd−$N_{pyridyl}$**	**0.2348**	**0.209533**	**−532.7163**	**499.2379**	**−33.4784**	**1.067059**	**−142.598**		
Nd1−N7	0.2917	0.2617	−727.0817	641.5415	−85.5401	1.133335	−293.266		
Nd1−N8	0.2997	0.2732	−752.5335	654.9458	−97.5878	1.149001	−325.636		
Nd1−N9	0.2749	0.244	−675.3851	614.7191	−60.6661	1.098689	−220.712		
**Average Nd−$N_{tetrazolate}$**	**0.2887**	**0.259633**	**−718.3334**	**637.0688**	**−81.2647**	**1.12756**	**−281.443**		

Sm1

Sm−L	*ρ(r)*	*δ(r)*	*V(r)*	*G(r)*	*H(r)*	*|V(r)|/G(r)*	*H(r)/ρ(r)*	*λ(Sm)*	*OS(M)*
Sm1−O1	0.3129	0.2131	−916.2451	918.9543	2.7091	0.997052	8.658049	58.6489	3.4
Sm1−O2	0.2982	0.2108	−856.9907	870.9280	13.937	0.983997	46.73993		
Sm1−O3	0.3345	0.2347	−1011.537	1002.234	−9.303	1.009283	−27.815		
**Average Sm−O**	**0.3152**	**0.2195**	**−928.2579**	**930.7056**	**2.4477**	**0.99737**	**7.765844**		
Sm1−N4	0.2452	0.2204	−565.4443	522.3015	−43.14	1.082601	−175.93		
Sm1−N5	0.2233	0.1937	−504.7314	488.7354	−15.99	1.032729	−71.62		
Sm1−N6	0.2325	0.2003	−531.0284	505.0527	−25.97	1.051432	−111.7		
**Average Sm−$N_{pyridyl}$**	**0.2337**	**0.2048**	**−533.7347**	**505.3632**	**−28.37**	**1.056141**	**−121.40**		
Sm1−N7	0.2940	0.2635	−740.5958	657.6227	−82.97	1.126171	−282.19		
Sm1−N8	0.3007	0.2753	−760.0560	665.4177	−94.64	1.142224	−314.78		
Sm1−N9	0.2386	0.2376	−685.5387	626.5133	−59.03	1.094213	−247.41		
**Average Sm−$N_{tetrazolate}$**	**0.2778**	**0.2588**	**−728.7302**	**649.8512**	**−78.87**	**1.12138**	**−283.99**		

Eu1

Eu−L	*ρ(r)*	*δ(r)*	*V(r)*	*G(r)*	*H(r)*	*|V(r)|/G(r)*	*H(r)/ρ(r)*	*λ(Eu)*	*OS(M)*
Eu1−O1	0.3092	0.2141	−911.1029	926.6307	15.5278	0.983243	50.21982	59.6609	3.3
Eu1−O2	0.2949	0.2107	−855.5669	883.3995	27.8327	0.968494	94.37245		
Eu1−O3	0.3284	0.2277	−996.1623	1002.3648	6.2025	0.993812	18.88953		
**Average Eu−O**	**0.3108**	**0.2175**	**−920.9441**	**937.4650**	**16.5210**	**0.982377**	**53.15195**		
Eu1−N4	0.2456	0.2181	−568.4668	526.7411	−41.7258	1.079215	−169.883		
Eu1−N5	0.2251	0.1919	−509.5390	491.3669	−18.1722	1.036983	−80.7227		
Eu1−N6	0.2343	0.2038	−537.5515	510.8886	−26.6629	1.052189	−113.78		
**Average Eu−$N_{pyridyl}$**	**0.2350**	**0.2046**	**−538.5191**	**509.6655**	**−28.8536**	**1.056613**	**−122.769**		
Eu1−N7	0.2984	0.2704	−756.0476	668.1222	−87.9254	1.131601	−294.674		
Eu1−N8	0.3022	0.2617	−771.7979	681.7707	−90.0273	1.132049	−297.953		
Eu1−N9	0.2778	0.2421	−687.1736	625.1599	−62.0137	1.099197	−223.233		
**Average Eu−$N_{tetrazolate}$**	**0.2928**	**0.258067**	**−738.3397**	**658.3509**	**−79.9888**	**1.121499**	**−273.207**		

Gd1

Gd−L	*ρ(r)*	*δ(r)*	*V(r)*	*G(r)*	*H(r)*	*|V(r)|/G(r)*	*H(r)/ρ(r)*	*λ(Gd)*	*OS(M)*
Gd1−O1	0.3075	0.2086	−910.0963	932.6793	22.5831	0.975787	73.43495	60.6779	3.3
Gd1−O2	0.2964	0.2075	−866.0008	897.1334	31.1326	0.965298	105.0195		
Gd1−O3	0.3301	0.2239	−1007.1005	1015.6551	8.5545	0.991577	25.91791		
**Average Gd−O**	**0.3113**	**0.213333**	**−927.7325**	**948.4892**	**20.7567**	**0.978116**	**66.6681**		
Gd1−N4	0.2442	0.2106	−566.9069	529.3179	−37.5892	1.071014	−153.905		
Gd1−N5	0.2226	0.1851	−503.9878	490.2359	−13.7520	1.028052	−61.7906		
Gd1−N6	0.2761	0.1949	−686.8698	631.9701	−54.8998	1.086871	−198.808		
**Average Gd−$N_{pyridyl}$**	**0.2476**	**0.196867**	**−585.9215**	**550.5079**	**−35.4136**	**1.064329**	**−143.001**		
Gd1−N7	0.2971	0.2512	−759.0292	680.0569	−78.9723	1.116126	−265.799		
Gd1−N8	0.3024	0.259	−773.8469	684.7898	−89.0571	1.13005	−294.521		
Gd1−N9	0.2761	0.2279	−686.8698	631.9701	−54.8998	1.086871	−198.808		
**Average Gd−$N_{tetrazolate}$**	**0.2919**	**0.246033**	**−739.9153**	**665.6056**	**−74.3097**	**1.111642**	**−254.591**		

Tb1

Tb−L	*ρ(r)*	*δ(r)*	*V(r)*	*G(r)*	*H(r)*	*|V(r)|/G(r)*	*H(r)/ρ(r)*	*λ(Tb)*	*OS(M)*
Tb1−O1	0.3248	0.2224	−960.8085	949.6029	−11.2056	1.0118	−34.5031	61.6823	3.3
Tb1−O2	0.3259	0.2302	−953.0447	928.5043	−24.5403	1.02643	−75.3031		
Tb1−O3	0.3407	0.2277	−1053.0047	1053.2147	0.2101	0.999801	0.616801		
**Average Tb−O**	**0.3305**	**0.226767**	**−988.9526**	**977.1073**	**−11.8453**	**1.012123**	**−35.8453**		
Tb1−N4	0.2460	0.2073	−573.9918	536.1965	−37.7952	1.070488	−153.639		
Tb1−N5	0.2215	0.1829	−499.3209	484.9147	−14.4062	1.029709	−65.0332		
Tb1−N6	0.2350	0.1904	−542.6129	518.3600	−24.2530	1.046788	−103.205		
**Average Tb−$N_{pyridyl}$**	**0.2342**	**0.193533**	**−538.6419**	**513.1571**	**−25.4848**	**1.049663**	**−108.829**		
Tb1−N7	0.3015	0.2518	−775.0205	691.3186	−83.7019	1.121076	−277.619		
Tb1−N8	0.3061	0.2565	−791.3649	702.0957	−89.2693	1.127147	−291.642		
Tb1−N9	0.2759	0.2199	−692.4001	643.9415	−48.4588	1.075253	−175.613		
**Average Tb−$N_{tetrazolate}$**	**0.2945**	**0.242733**	**−752.9285**	**679.1186**	**−73.8100**	**1.108685**	**−250.619**		

Dy1

Dy−L	*ρ(r)*	*δ(r)*	*V(r)*	*G(r)*	*H(r)*	*|V(r)|/G(r)*	*H(r)/ρ(r)*	*λ(Dy)*	*OS(M)*
Dy1−O1	0.3269	0.221	−973.9885	965.2069	−8.7816	1.009098	−26.8612	62.6846	3.3
Dy1−O2	0.3308	0.2313	−980.4964	958.7820	−21.7143	1.022648	−65.644		
Dy1−O3	0.3418	0.2271	−1062.5748	1066.4626	3.8880	0.996354	11.37327		
**Average Dy−O**	**0.3332**	**0.226467**	**−1005.6866**	**996.8172**	**−8.8693**	**1.008898**	**−26.6196**		
Dy1−N4	0.2462	0.205	−576.0577	539.3841	−36.6736	1.067992	−148.942		
Dy1−N5	0.2208	0.1807	−500.9635	490.8097	−10.1539	1.020688	−45.9776		
Dy1−N6	0.2360	0.1901	−548.5094	526.1901	−22.3193	1.042417	−94.5806		
**Average Dy−$N_{pyridyl}$**	**0.2344**	**0.191933**	**−541.8435**	**518.7947**	**−23.0489**	**1.044428**	**−98.352**		
Dy1−N7	0.3019	0.249	−779.7139	698.8976	−80.8163	1.115634	−267.71		
Dy1−N8	0.3084	0.2556	−806.8559	721.7747	−85.0811	1.117878	−275.84		
Dy1−N9	0.2748	0.2163	−693.9567	652.2859	−41.6709	1.063884	−151.657		
**Average Dy−$N_{tetrazolate}$**	**0.2950**	**0.2403**	**−760.1755**	**690.9860**	**−69.1894**	**1.100131**	**−234.516**		

Ho1

Ho−L	*ρ(r)*	*δ(r)*	*V(r)*	*G(r)*	*H(r)*	*|V(r)|/G(r)*	*H(r)/ρ(r)*	*λ(Ho)*	*OS(M)*
Ho1−O1	0.3353	0.2235	−1016.7037	1008.0710	−8.6328	1.008564	−25.7427	63.6944	3.3
Ho1−O2	0.3274	0.2166	−1000.5442	1016.1111	15.5669	0.98468	47.55236		
Ho1−O3	0.3320	0.2168	−1023.6901	1039.1819	15.4919	0.985092	46.66616		
**Average Ho−O**	**0.3316**	**0.218967**	**−1013.6460**	**1021.1213**	**7.4754**	**0.992679**	**22.54592**		
Ho1−N4	0.2399	0.2002	−561.3213	535.7527	−25.5687	1.047725	−106.561		
Ho1−N5	0.2182	0.1756	−493.2965	485.7678	−7.5286	1.015498	−34.5087		
Ho1−N6	0.2393	0.1888	−562.1394	539.8224	−22.3171	1.041341	−93.242		
**Average Ho−$N_{pyridyl}$**	**0.2325**	**0.1882**	**−538.9190**	**520.4476**	**−18.4715**	**1.035491**	**−79.4522**		
Ho1−N7	0.3086	0.2469	−809.8133	726.7734	−83.0400	1.114258	−269.057		
Ho1−N8	0.3117	0.2531	−819.5140	731.1041	−88.4099	1.120927	−283.594		
Ho1−N9	0.2760	0.2187	−700.4695	659.7326	−40.7369	1.061748	−147.587		
**Average Ho−$N_{tetrazolate}$**	**0.2988**	**0.239567**	**−776.5990**	**705.8700**	**−70.7289**	**1.100201**	**−236.71**		

Pu1

Pu−L	$\rho(r)$	$\delta(r)$	$V(r)$	$G(r)$	$H(r)$	$\lvert V(r)\rvert/G(r)$	$H(r)/\rho(r)$	$\lambda(Pu)$	$OS(M)$
Pu1−O1	0.3320	0.2508	−993.7238	979.3227	−14.4011	1.014705	−43.3822	90.4883	3.5
Pu1−O2	0.3192	0.2465	−943.1342	941.6967	−1.4375	1.001526	−4.50295		
Pu1−O3	0.3575	0.2782	−1117.1968	1093.8974	−23.2995	1.0213	−65.18		
**Average Pu−O**	**0.3362**	**0.2585**	**−1018.0183**	**1004.9723**	**−13.0460**	**1.012981**	**−38.802**		
Pu1−N4	0.2693	0.252	−650.7010	590.1093	−60.5917	1.102679	−225.014		
Pu1−N5	0.2492	0.2334	−596.7717	568.6068	−28.1649	1.049533	−113.04		
Pu1−N6	0.2533	0.232	−602.6063	562.6845	−39.9218	1.070949	−157.579		
**Average Pu−$N_{pyridyl}$**	**0.2573**	**0.239133**	**−616.6930**	**573.8002**	**−42.8928**	**1.074752**	**−166.729**		
Pu1−N7	0.3154	0.2988	−825.7982	725.6872	−100.1110	1.137953	−317.37		
Pu1−N8	0.3209	0.3085	−843.3060	733.7512	−109.5547	1.149308	−341.38		
Pu1−N9	0.3022	0.2773	−787.8166	713.3465	−74.4701	1.104395	−246.386		
**Average Pu−$N_{tetrazolate}$**	**0.3129**	**0.294867**	**−818.9736**	**724.2616**	**−94.7119**	**1.13077**	**−302.721**		

Am1

Am−L	$\rho(r)$	$\delta(r)$	$V(r)$	$G(r)$	$H(r)$	$\lvert V(r)\rvert/G(r)$	$H(r)/\rho(r)$	$\lambda(Am)$	$OS(M)$
Am1−O1	0.3286	0.2431	−988.5748	985.8251	−2.7498	1.002789	−8.36753	91.5303	3.5
Am1−O2	0.3323	0.2612	−978.7822	947.7088	−31.0734	1.032788	−93.5101		
Am1−O3	0.3426	0.2608	−1058.9028	1055.1078	−3.7951	1.003597	−11.0764		
**Average Am−O**	**0.3345**	**0.255033**	**−1008.7533**	**996.2139**	**−12.5394**	**1.012587**	**−37.485**		
Am1−N4	0.2707	0.2422	−660.0722	602.3722	−57.7000	1.095788	−213.112		
Am1−N5	0.2493	0.2328	−593.0494	560.3723	−32.6770	1.058313	−131.05		
Am1−N6	0.2608	0.2281	−630.5674	586.7332	−43.8341	1.074709	−168.068		
**Average Am−$N_{pyridyl}$**	**0.2603**	**0.234367**	**−627.8963**	**583.1592**	**−44.7370**	**1.076715**	**−171.865**		
Am1−N7	0.3215	0.3022	−848.0025	740.0220	−107.9806	1.145915	−335.816		
Am1−N8	0.3301	0.3054	−879.2887	759.6650	−119.6236	1.157469	−362.348		
Am1−N9	0.3086	0.2665	−815.7787	738.6388	−77.1400	1.104435	−249.929		
**Average Am−$N_{tetrazolate}$**	**0.3201**	**0.291367**	**−847.6900**	**746.1086**	**−101.5814**	**1.136148**	**−317.333**		

Cm1

Cm−L	*ρ(r)*	*δ(r)*	*V(r)*	*G(r)*	*H(r)*	*|V(r)|/G(r)*	*H(r)/ρ(r)*	*λ(Cm)*	*OS(M)*
Cm1−O1	0.3275	0.24	−978.9736	972.3507	−6.6229	1.006811	−20.2235	92.5702	3.4
Cm1−O2	0.3324	0.2582	−980.9795	951.8286	−29.1509	1.030626	−87.7104		
Cm1−O3	0.3499	0.2523	−1087.1042	1073.8146	−13.2895	1.012376	−37.9845		
**Average Cm−O**	**0.3366**	**0.250167**	**−1015.6857**	**999.3313**	**−16.3545**	**1.016365**	**−48.5916**		
Cm1−N4	0.2675	0.2379	−650.4091	597.4643	−52.9448	1.088616	−197.945		
Cm1−N5	0.2499	0.2187	−595.9453	563.7331	−32.2121	1.057141	−128.885		
Cm1−N6	0.2585	0.2221	−626.0256	587.6945	−38.3310	1.065223	−148.298		
**Average Cm−$N_{pyridyl}$**	**0.2586**	**0.226233**	**−624.1267**	**582.9639**	**−41.1627**	**1.070609**	**−159.16**		
Cm1−N7	0.3251	0.2845	−868.5687	763.4799	−105.0889	1.137644	−323.25		
Cm1−N8	0.3265	0.2888	−871.0188	761.3088	−109.7100	1.144107	−336.002		
Cm1−N9	0.3037	0.2569	−795.6269	722.1961	−73.4308	1.101677	−241.81		
**Average Cm−$N_{tetrazolate}$**	**0.3184**	**0.276733**	**−845.0714**	**748.9949**	**−96.0765**	**1.128274**	**−301.72**		

Section S8: Wiberg bond indices (WBIs)

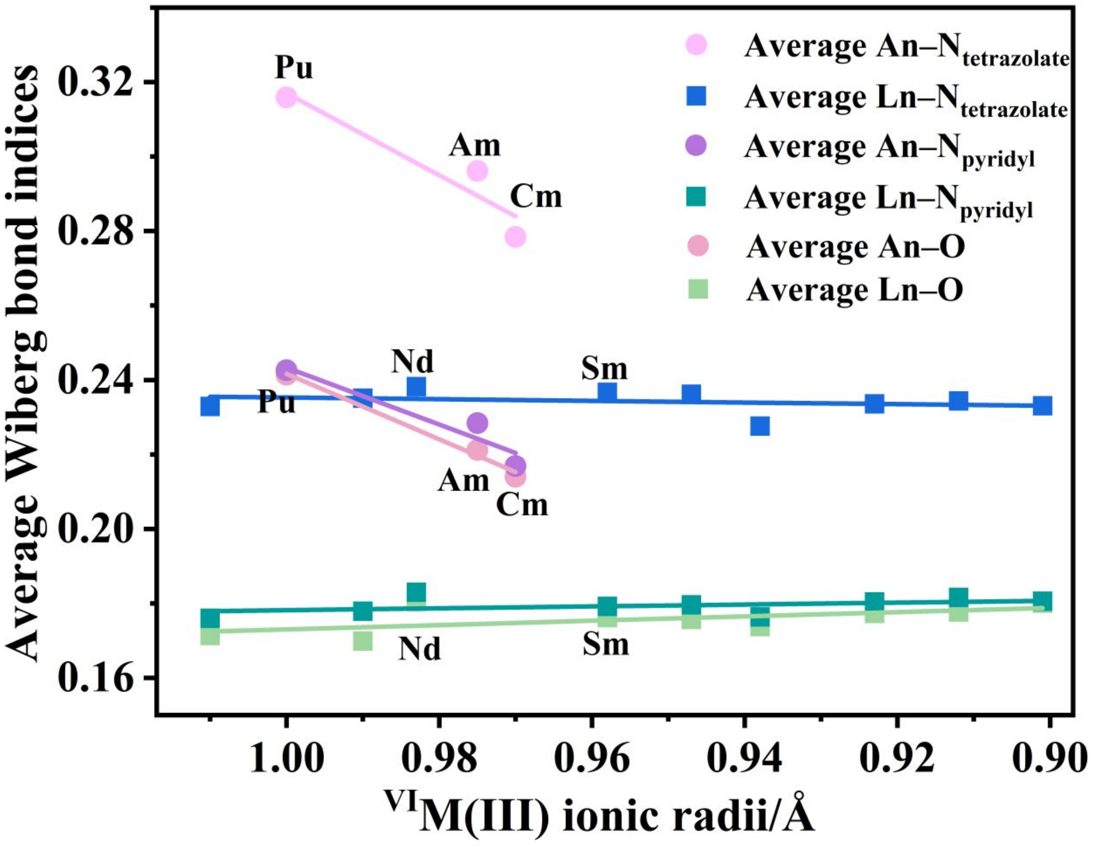

Figure S8.1. Wiberg bond indices (WBI).

Table S8.1. Wiberg bond indices (WBI). (Corresponding atoms numbers within crystal structures are shown in Section S6)

Bond	Ce1	Pr1	Nd1	Sm1	Eu1	Gd1	Tb1	Dy1	Ho1	Pu1	Am1	Cm1
M–O2	0.1601	0.1580	0.1694	0.1642	0.1636	0.1626	0.1670	0.1679	0.1702	0.2172	0.2125	0.2052
M–O3	0.1738	0.1741	0.1908	0.1867	0.1850	0.1845	0.1802	0.1816	0.1877	0.2501	0.2198	0.2160
M–O4	0.1803	0.1775	0.1813	0.1776	0.1785	0.1742	0.1847	0.1837	0.1823	0.2570	0.2314	0.2208
**M–O Average**	**0.1714**	**0.1699**	**0.1805**	**0.1762**	**0.1757**	**0.1738**	**0.1773**	**0.1777**	**0.1801**	**0.2414**	**0.2212**	**0.2140**
M–N5	0.1872	0.1914	0.1972	0.1940	0.1909	0.1892	0.1913	0.1910	0.1907	0.2570	0.2357	0.2282
M–N7	0.1719	0.1711	0.1748	0.1698	0.1693	0.1682	0.1753	0.1765	0.1736	0.2355	0.2293	0.2128
M–N9	0.1688	0.1712	0.1771	0.1738	0.1786	0.1718	0.1745	0.1772	0.1775	0.2352	0.2203	0.2098
**M–$N_{pyridyl}$ Average**	**0.1760**	**0.1779**	**0.1830**	**0.1792**	**0.1796**	**0.1764**	**0.1804**	**0.1816**	**0.1806**	**0.2426**	**0.2284**	**0.2169**
M–N11	0.2379	0.2379	0.2416	0.2427	0.2497	0.2327	0.2426	0.2448	0.2398	0.3210	0.3087	0.2848
M–N12	0.2451	0.2488	0.2494	0.2497	0.2340	0.2365	0.2437	0.2429	0.2426	0.3324	0.3103	0.2892
M–N13	0.2158	0.2186	0.2237	0.2176	0.2249	0.2135	0.2146	0.2154	0.2170	0.2944	0.2693	0.2611

**M–$N_{tetrazolate}$ Average**	**0.2329**	**0.2351**	**0.2382**	**0.2367**	**0.2362**	**0.2276**	**0.2336**	**0.2344**	**0.2331**	**0.3159**	**0.2961**	**0.2784**
**M–N Average**	**0.2045**	**0.2065**	**0.2106**	**0.2079**	**0.2079**	**0.2020**	**0.2070**	**0.2080**	**0.2069**	**0.2793**	**0.2623**	**0.2477**

Table S8.2. ΔWBI between **Nd1** with **Pu1**, **Am1** and **Cm1**.

Bond	Δ **Nd1** with **Pu1**	Δ**Nd1** with **Am1**	Δ **Nd1** with **Cm1**
Average M–O	0.0609	0.0407	0.0335
Average M–$N_{pyridine}$	0.0595	0.0454	0.0339
Average M–$N_{tetrazolate}$	0.0777	0.0579	0.0401

Table S8.3. ΔWBI between **Sm1** with **Pu1**, **Am1** and **Cm1**.

Bond	Δ **Sm1** with **Pu1**	Δ **Sm1** with **Am1**	Δ **Sm1** with **Cm1**
Average M–O	0.0653	0.0451	0.0378
Average M–$N_{pyridine}$	0.0634	0.0492	0.0377
Average M–$N_{tetrazolate}$	0.0793	0.0594	0.0417

Section S9: Interacting quantum atoms (IQA)

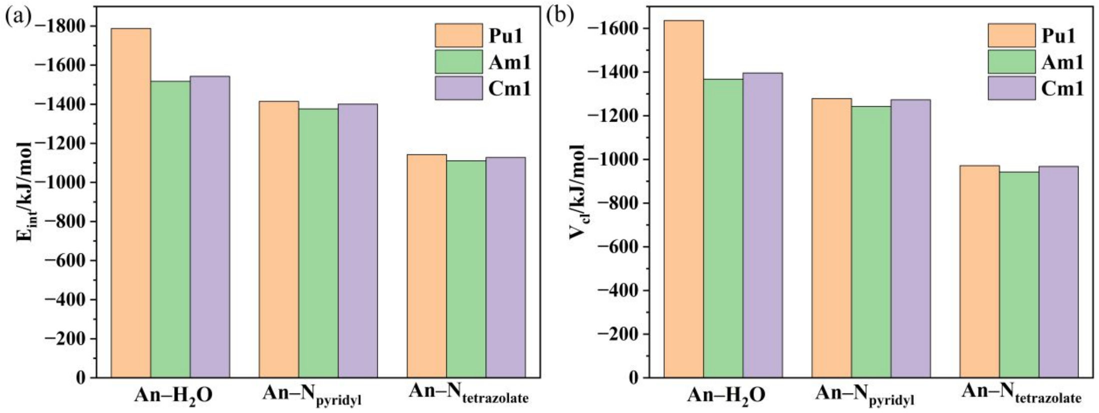

Figure S9.1. The energy decomposition on the base of IQA definition including (a) $E_{int} = V_{nn} + V_{en} + V_{ne} + V_{ee}$, (b) $V_{cl} = V_{nn} + V_{en} + V_{ne} + V_{eeC}$

Table S9.1 The energy decomposition on the base of IQA definition (au).

E_IQA_Inter(A,B)/2 = E_{int} = V_{ne}(A,B)/2 + V_{en}(A,B)/2 + V_{ee}(A,B)/2 + V_{nn}(A,B)/2 = Contribution to E_IQA(A) from "interaction" of Atom A With Atom B = Half of total IQA "interaction" energy between atom A and atom B.

V_{ne}(A,B)/2 = Half of attraction energy between nucleus of atom A and electron density distribution of atom B

V_{en}(A,B)/2 = Half of attraction energy between electron density distribution of atom A and nucleus of Atom B

V_{ee}(A,B)/2 = Half of two-electron interaction energy between atom A and atom B

V_{nn}(A,B)/2 = Half of repulsion energy between nucleus of atom A and nucleus of atom B

V_{ee}C(A,B)/2 = Coulomb part of Vee(A,B)/2

V_{ee}Cl(A,B)/2 = the total classical electrostatic interaction between atom A and atom B = nuclear–nuclear repulsive energy + electron-electron coulombic repulsion energy + the electron-nuclear attraction energy between atom A and atom B.

V_{ee}X(A,B)/2 = Exchange-correlation part of Vee(A,B)/2

The exchange–correlation component (V_{ee}X(A,B)) serves as a reliable indicator of the covalent contribution to the interatomic energy.

V_{cl} = the total classical electrostatic interaction between atom A and atom B = nuclear-nuclear repulsive energy + electron-electron coulombic repulsion energy + the electron-nuclear attraction energy between atom A and atom B.

V_{XC} = exchange-correlation part of two-electron interaction energy between atom A and atom B.

V_{XC}/E_{int} = exchange-correlation contributions to total interaction energy between atom A and atom B.[13]

A	B	E_{int}(A,B)/2	V_{ne} (A,B)/2	V_{en} (A,B)/2	V_{ee} (A,B)/2	V_{nn} (A,B)/2	V_{eeC} (A,B)/2	V_{eeCl} (A,B)/2	V_{eeX} (A,B)/2	V_{eeX} /E_{int}
Pu1	O2	−0.2900	−91.8762	−78.3783	89.7865	80.1780	89.8143	−0.2622	−0.0278	0.0960
Pu1	O3	−0.3534	−94.5523	−78.3312	92.3774	80.1526	92.4048	−0.3260	−0.0274	0.0776
Pu1	O4	−0.3783	−97.2836	−80.1070	95.0438	81.9685	95.0753	−0.3467	−0.0316	0.0834
**Average Pu–O**		**−0.3406**	**−94.5707**	**−78.9388**	**92.4026**	**80.7664**	**92.4315**	**−0.3117**	**−0.0289**	**0.0849**
Pu1	N5	−0.2721	−75.8407	−63.5616	74.0946	65.0355	74.1218	−0.2450	−0.0271	0.0998
Pu1	N7	−0.2656	−75.0557	−62.8957	73.3487	64.3371	73.3741	−0.2402	−0.0254	0.0955
Pu1	N9	−0.2708	−75.2994	−62.9307	73.5815	64.3778	73.6067	−0.2456	−0.0252	0.0931
**Average Pu–$N_{pyridyl}$**		**−0.2695**	**−75.3986**	**−63.1293**	**73.6749**	**64.5835**	**73.7008**	**−0.2436**	**−0.0259**	**0.0961**
Pu1	N11	−0.2143	−75.1936	−65.6736	73.4571	67.1958	73.4902	−0.1812	−0.0331	0.1543
Pu1	N12	−0.2231	−75.6631	−65.8161	73.9110	67.3451	73.9451	−0.1890	−0.0341	0.1529
Pu1	N13	−0.2155	−74.8605	−65.1564	73.1416	66.6599	73.1722	−0.1849	−0.0306	0.1421
**Average Pu–$N_{tetrazolate}$**		**−0.2176**	**−75.2391**	**−65.5487**	**73.5033**	**67.0669**	**73.5359**	**−0.1850**	**−0.0326**	**0.1498**

A	B	E_{int}(A,B)/2	V_{ne} (A,B)/2	V_{en} (A,B)/2	V_{ee} (A,B)/2	V_{nn} (A,B)/2	V_{eeC} (A,B)/2	V_{eeCl} (A,B)/2	V_{eeX} (A,B)/2	V_{eeX} /E_{int}
Am1	O2	−0.2905	−93.6299	−79.7122	91.5722	81.4794	91.5997	−0.2630	−0.0275	0.0947
Am1	O3	−0.2894	−92.9725	−79.2474	90.9149	81.0156	90.9439	−0.2604	−0.0290	0.1002
Am1	O6	−0.2872	−94.5867	−80.9893	92.4781	82.8106	92.5073	−0.2581	−0.0292	0.1017
**Average Am–O**		**−0.2890**	**−93.7297**	**−79.9830**	**91.6551**	**81.7685**	**91.6836**	**−0.2605**	**−0.0286**	**0.0988**
Am1	N9	−0.2647	−76.8497	−64.5072	75.1499	65.9423	75.1771	−0.2375	−0.0272	0.1028
Am1	N11	−0.2594	−76.0895	−63.8455	74.4110	65.2646	74.4349	−0.2355	−0.0239	0.0921
Am1	N13	−0.2626	−76.4848	−64.1170	74.8033	65.5359	74.8289	−0.2370	−0.0255	0.0971
**Average Am–$N_{pyridyl}$**		**−0.2622**	**−76.4747**	**−64.1566**	**74.7881**	**65.5809**	**74.8136**	**−0.2367**	**−0.0255**	**0.0974**
Am1	N15	−0.2125	−76.4425	−66.7472	74.7317	68.2455	74.7642	−0.1800	−0.0325	0.1529
Am1	N16	−0.2140	−76.6467	−66.9282	74.9302	68.4307	74.9638	−0.1804	−0.0336	0.1570
Am1	N17	−0.2084	−75.9240	−66.2226	74.2489	67.6894	74.2789	−0.1783	−0.0300	0.1440
**Average Am–$N_{tetrazolate}$**		**−0.2116**	**−76.3377**	**−66.6327**	**74.6369**	**68.1219**	**74.6690**	**−0.1796**	**−0.0320**	**0.1514**

A	B	E_{int}(A,B)/2	V_{ne} (A,B)/2	V_{en} (A,B)/2	V_{ee} (A,B)/2	V_{nn} (A,B)/2	V_{eeC} (A,B)/2	V_{eeCl} (A,B)/2	V_{eeX} (A,B)/2	V_{eeX} /E_{int}
Cm1	O2	−0.2944	−94.7136	−80.6559	92.6160	82.4592	92.6429	−0.2675	−0.0269	0.0914
Cm1	O3	−0.2956	−94.3892	−80.4120	92.2857	82.2198	92.3142	−0.2671	−0.0285	0.0964
Cm1	O6	−0.2916	−95.7822	−81.9940	93.6360	83.8486	93.6645	−0.2630	−0.0286	0.0980
**Average**		**−0.2939**	**−94.9617**	**−81.0206**	**92.8459**	**82.8425**	**92.8739**	**−0.2659**	**−0.0280**	**0.0953**

**Cm–O**										
Cm1	N9	−0.2694	−77.8910	−65.3371	76.1519	66.8068	76.1774	−0.2439	−0.0255	0.0947
Cm1	N11	−0.2632	−76.9945	−64.5809	75.2884	66.0239	75.3117	−0.2399	−0.0233	0.0885
Cm1	N13	−0.2677	−77.5640	−64.9672	75.8441	66.4194	75.8681	−0.2437	−0.0240	0.0896
**Average Cm–N$_{pyridyl}$**		**−0.2668**	**−77.4832**	**−64.9617**	**75.7615**	**66.4167**	**75.7857**	**−0.2425**	**−0.0243**	**0.0910**
Cm1	N15	−0.2138	−77.4652	−67.6653	75.7260	69.1907	75.7574	−0.1824	−0.0314	0.1467
Cm1	N16	−0.2177	−77.6129	−67.6658	75.8659	69.1951	75.8976	−0.1861	−0.0316	0.1452
Cm1	N17	−0.2131	−76.9893	−67.0423	75.2770	68.5416	75.3053	−0.1847	−0.0283	0.1330
**Average Cm–N$_{tetrazolate}$**		**−0.2148**	**−77.3558**	**−67.4578**	**75.6230**	**68.9758**	**75.6534**	**−0.1844**	**−0.0304**	**0.1417**

Section S10: Absorption UV-vis-NIR spectrum of Pu1

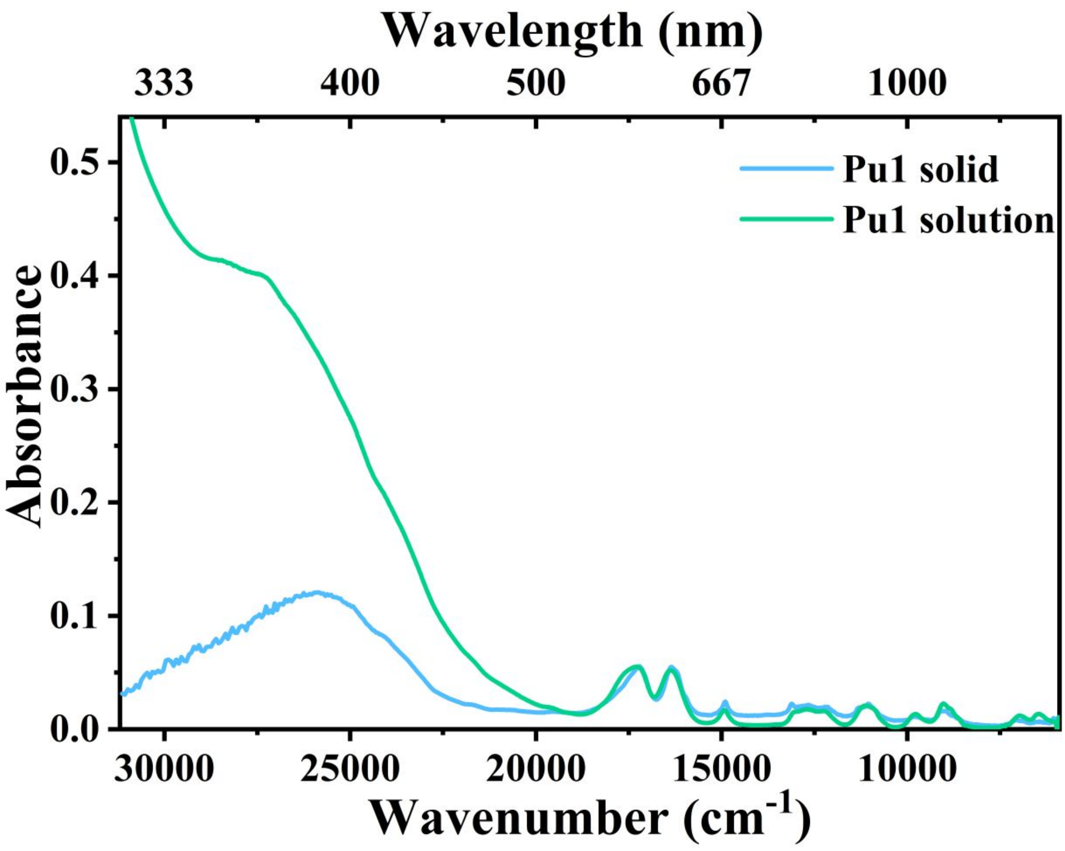

Figure S10.1. Solid state and solution phase in DMSO UV-vis-NIR absorption spectra of **Pu1**.

Section S11: CASSCF Calculations

To verify the accuracy of the DFT results, the same ground state geometry as was used in the DFT calculations, that being the crystal structures, with optimized hydrogens, of the **Pu1**, **Am1,** and **Cm1** structures were used to calculate the multiconfigurational wavefunction at the CASSCF with NEVPT2 correction level of theory with the all electron SARC-DKH-TZVPP basis set and functional for the metal, and DKH-def2-SVP for all other atoms. Relativistic corrections including "picturechange" and "dosoc" inputs on ORCA were included as spin orbit coupling corrections required for actinide complexes. The CASSCF active space included all 5*f* orbitals and electrons, as well as the two highest spin states (for example Cm multiplicities 8 and 6 were included). 30 roots were included for **Cm1** and **Am1** and 91 roots for **Pu1**, these roots account for each of the highest multiplicity spin states and at least half of the second highest multiplicity (for example **Pu1** includes 91 roots, 21 for the 21 sextets and 70 of the 140 quartet states.

The electronic structures of **Pu1**, **Am1** and **Cm1** were analyzed through complete-active-space multi-configuration approach with second order perturbation theoretical correction (NEVPT2) implemented in ORCA 6.1.1 program.[14] Then we used the CASSCF ground-state wavefunction to obtain orbital compositions via natural localized molecular orbital (NLMO) analysis from the NBO 7.0 program,[17] while molecular electron densities were examined using QTAIM calculations performed with MultiWFN.[18] **Figure S11.1.** NLMO. The electron density of M–N bonds of **Pu1**, **Am1** and **Cm1**, are shown in Table S11.2.

(a) (b)

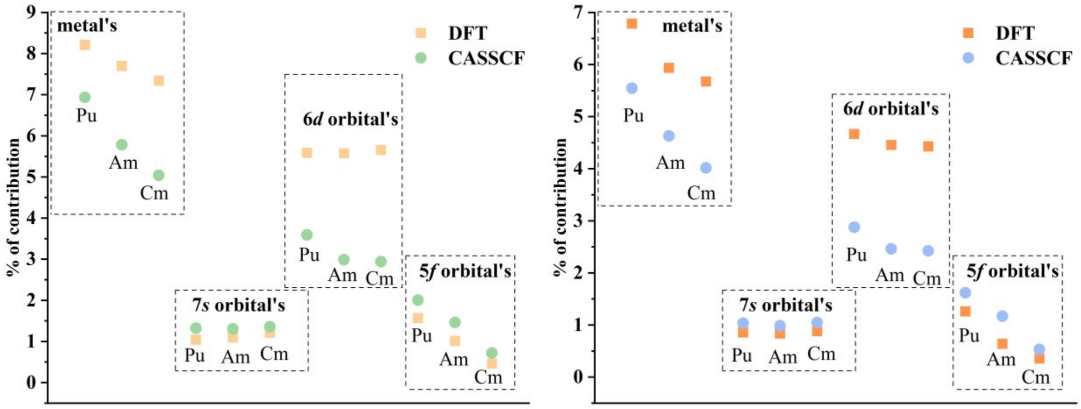

Figure S11.1. The comparison of NLMO contribution between CASSCF and DFT to average (a) M–$N_{tetrazolate}$ and (b) M–$N_{pyridyl}$ bonds of **Pu1**, **Am1** and **Cm1**.

Table S11.1. The comparison of NLMO contribution between CASSCF and DFT to Average M–$N_{tetrazolate}$ and Average M–$N_{pyridyl}$ bonds of **Pu1**, **Am1** and **Cm1**.

Complex	Bond		CASSCF	DFT
**Pu1**	Average Pu–$N_{pyridyl}$	metal	5.55	6.79
		7*s*	1.04	0.86
		6*d*	2.88	4.67
		5*f*	2.01	1.26
	Average Pu–$N_{tetrazolate}$	metal	6.94	8.21
		7*s*	1.32	1.04
		6*d*	3.59	5.59
		5*f*	1.62	1.57
**Am1**	Average Am–$N_{pyridyl}$	metal	4.63	5.94
		7*s*	0.99	0.84
		6*d*	2.46	4.46
		5*f*	1.17	0.64
	Average Am–$N_{tetrazolate}$	metal	5.78	7.70
		7*s*	1.31	1.10
		6*d*	2.99	5.58
		5*f*	1.47	1.01
**Cm1**	Average Cm–$N_{pyridyl}$	metal	4.02	5.68
		7*s*	1.05	0.88
		6*d*	2.42	4.43
		5*f*	0.53	0.36
	Average Cm–$N_{tetrazolate}$	metal	5.04	7.34
		7*s*	1.36	1.21
		6*d*	2.94	5.66
		5*f*	0.72	0.46

Table S11.2. QTAIM metrics the electron density ρ(r) in e·Å^{-3} and a.u. of M–N$_{tetrazolate}$ and M–N$_{pyridyl}$ bonds in **Pu1**, **Am1** and **Cm1**, at the bond critical points (BCPs).[16]

**Bonds**	ρ(r) in e·Å^{-3}	ρ(r) in a.u.
**Complex**	**Pu1**	
Pu–N6	0.2510	3.72E-02
Pu–N11	0.2463	3.65E-02
Pu–N1	0.2362	3.50E-02
**Average Pu–N$_{pyridyl}$**	**0.2450**	**3.63E-02**
Pu–N7	0.3070	4.55E-02
Pu–N12	0.2909	4.31E-02
Pu–N2	0.3037	4.50E-02
**Average Pu–N$_{tetrazolate}$**	**0.3003**	**4.45E-02**
**Bonds**	ρ(r) in e·Å^{-3}	ρ(r) in a.u.
**Complex**	**Am1**	
Am–N6	0.2483	3.68E-02
Am–N11	0.2375	3.52E-02
Am–N1	0.2335	3.46E-02
**Average Am–N$_{pyridyl}$**	**0.2396**	**3.55E-02**
Am–N7	0.3070	4.55E-02
Am–N12	0.2753	4.08E-02
Am–N2	0.3010	4.46E-02
**Average Am–N$_{tetrazolate}$**	**0.2942**	**4.36E-02**
**Bonds**	ρ(r) in e·Å^{-3}	ρ(r) in a.u.
**Complex**	**Cm1**	
Cm–N6	0.2470	3.66E-02
Cm–N11	0.2382	3.53E-02
Cm–N1	0.2294	3.40E-02
**Average Cm–N$_{pyridyl}$**	**0.2382**	**3.53E-02**
Cm–N7	0.3023	4.48E-02
Cm–N12	0.2814	4.17E-02
Cm–N2	0.3030	4.49E-02
**Average Cm–N$_{tetrazolate}$**	**0.2956**	**4.38E-02**

Table S11.3. Comparison between DFT and CASSCF for QTAIM metrics the electron density ρ(r) in e·Å^{-3} of average M–N$_{tetrazolate}$ and M–N$_{pyridyl}$ bonds in **Pu1**, **Am1** and **Cm1**, at the bond critical points (BCPs).[16]

	CASSCF	DFT	Difference
Average Pu–N$_{pyridyl}$	0.2450	0.2573	0.0123
Average Am–N$_{pyridyl}$	0.2396	0.2603	0.0207
Average Cm–N$_{pyridyl}$	0.2382	0.2586	0.0204
Average Pu–N$_{tetrazolate}$	0.3003	0.3129	0.0126
Average Am–N$_{tetrazolate}$	0.2942	0.3201	0.0259
Average Cm–N$_{tetrazolate}$	0.2956	0.3184	0.0228

Frequency Calculations

To further analyze the crystal structure geometry to confirm the structure is in a ground state local minimum, the frequencies were calculated for each **An1** complex that was used for DFT calculations. However, it is known that small magnitude imaginary nodes arise in DFT calculations from noise, which is amplified by heavy elements and relativistic effects when a large enough exchange correlation integration grid is not calculated. Unfortunately, calculating an intense enough integration grid for the actinide series is often not computationally feasible. With a PBE/TZP all electron basis set and functional each frequency calculation resulted in confidence in the local minimum ground state structure. However, each calculation resulted in a few negative frequencies with a magnitude less than than 60 cm^{-1}. This is not unexpected because the grid produced by PBE is not of high enough quality to show no imaginary frequencies for actinide systems, but running a frequency calculation with an increased grid to achieve only positive frequencies is not computationally feasible at this time. These results in combination with the multiconfiguration CASSCF/NEVPT2 give the authors confidence in the DFT results. All frequency calculations were performed on AMS software with the DFT input PBE/TZP no frozen core and COSMO solvent "Water" with the numerical quality upgraded to "VeryGood."

Cm1 frequency results

Index	Frequency (cm-1)	Red. mass (u)	F const (Ha/Bohr^2)	Intensity (km/mol)
7	50.3174	1.2723	0.000122	49.3233
8	-46.4247	5.3016	-0.000432	1.2766
9	-34.4393	4.8484	-0.000218	10.0299
10	-39.7437	5.8670	-0.000351	2.4647
11	28.9780	5.4574	0.000173	3.1818
12	-10.5696	6.6980	-0.000028	14.2561
13	-24.3573	7.9072	-0.000178	13.8296
14	-13.9001	7.4043	-0.000054	18.7952
15	21.5777	7.7759	0.000137	12.6353
16	43.0043	5.6467	0.000395	23.5080
17	64.6280	5.8720	0.000928	0.9078
18	69.3602	5.6384	0.001027	10.5781
19	71.6076	4.5035	0.000874	26.9343
20	84.1635	5.3842	0.001443	1.1748
21	90.5797	5.9257	0.001840	1.0777
22	91.1496	5.0732	0.001595	1.5613
23	95.6451	4.9643	0.001719	6.0007
24	100.3625	5.9058	0.002251	4.3966
25	104.6943	7.4662	0.003097	4.2553
26	112.9551	6.7403	0.003254	3.3704
27	122.0799	7.9016	0.004457	38.4140
28	124.9740	10.5284	0.006223	24.2879
29	127.6256	8.2147	0.005064	44.3106

30	136.6843	13.0750	0.009244	34.9193
31	148.5033	8.0384	0.006709	24.2989
32	156.8538	8.6333	0.008038	14.4711
33	176.5423	4.1320	0.004874	18.5959
34	179.7770	5.2376	0.006406	4.7409
35	182.8850	6.6401	0.008405	8.7664
36	185.5538	4.6075	0.006003	17.2219
37	195.2222	3.3797	0.004874	30.3716
38	225.6432	1.5106	0.002911	176.5884
39	248.6291	1.7598	0.004117	47.2681
40	270.6768	3.3532	0.009297	173.6733
41	274.2953	5.0707	0.014438	27.8150
42	277.9958	2.5862	0.007564	46.0639
43	281.8732	3.1514	0.009476	71.9618
44	283.1787	3.3945	0.010301	114.4999
45	287.4195	2.7661	0.008648	84.9968
46	313.8301	6.7143	0.025025	24.2931
47	366.5816	8.4719	0.043084	18.6038
48	367.6974	8.6428	0.044221	10.4250
49	370.2384	9.2307	0.047884	5.6708
50	382.0074	1.2760	0.007047	130.1028
51	389.5042	3.0075	0.017267	46.1234
52	397.4483	2.7743	0.016584	29.6427
53	401.3342	3.7628	0.022936	12.8506
54	420.9457	1.2218	0.008193	102.9012
55	455.8580	1.3070	0.010278	228.2072
56	466.1526	6.7251	0.055303	3.5589
57	470.8522	7.1593	0.060066	3.8473
58	471.3326	5.4040	0.045432	34.9824
59	494.7159	3.2610	0.030204	5.3838
60	497.9261	2.7486	0.025789	18.4730
61	499.5755	3.4970	0.033029	2.1693
62	515.7283	1.2326	0.012407	193.7573
63	577.4077	1.1081	0.013981	170.2973
64	628.4834	7.0492	0.105370	20.8653
65	630.6765	7.0172	0.105626	9.3920
66	632.2762	6.9967	0.105852	10.0283
67	711.6184	6.4409	0.123434	21.0409
68	713.0142	7.0430	0.135501	22.4197
69	713.5976	7.0799	0.136434	26.8179
70	713.9129	8.8021	0.169772	11.0155
71	714.4477	9.2713	0.179090	1.9041
72	714.5003	8.8509	0.170995	5.2744
73	718.9222	3.6596	0.071580	83.1038

74	719.7258	4.1930	0.082196	42.5398
75	720.7784	3.5745	0.070277	24.3890
76	732.9037	1.9497	0.039632	25.6311
77	733.9565	1.9478	0.039708	28.8199
78	734.6791	2.1151	0.043203	17.0749
79	784.1793	3.3690	0.078401	18.6783
80	784.7982	3.4381	0.080135	21.9771
81	786.0203	3.1388	0.073389	20.3490
82	872.7560	1.3471	0.038832	0.7846
83	875.0305	1.3442	0.038948	0.8588
84	882.4596	1.3338	0.039308	0.5764
85	949.9519	1.4061	0.048017	2.9827
86	955.2097	1.3866	0.047877	6.2450
87	961.0467	1.3851	0.048411	2.7016
88	982.7036	1.3302	0.048611	0.8981
89	984.0078	1.3284	0.048676	0.5929
90	988.6959	1.3102	0.048467	0.1040
91	1001.1752	6.8309	0.259113	27.0775
92	1004.2906	7.4040	0.282602	20.9976
93	1005.2542	7.0337	0.268986	17.2062
94	1010.2006	10.8998	0.420946	4.7567
95	1015.3123	10.6834	0.416775	15.4895
96	1016.7634	10.4451	0.408645	9.0686
97	1031.5531	12.3082	0.495644	25.2871
98	1033.3828	11.5302	0.465964	37.8541
99	1035.7719	11.4808	0.466113	32.1539
100	1055.5248	1.9981	0.084247	27.7329
101	1059.5945	6.4063	0.272195	3.4909
102	1061.4948	2.1310	0.090870	24.1038
103	1063.0666	2.6571	0.113637	18.5102
104	1067.3127	5.3911	0.232410	4.2450
105	1076.5247	8.8569	0.388437	1.9204
106	1098.8318	1.7610	0.080468	12.0478
107	1099.3703	1.5766	0.072112	20.9376
108	1110.5015	1.8067	0.084318	4.0786
109	1115.0570	10.9149	0.513576	37.9624
110	1117.1522	10.6954	0.505144	41.9900
111	1122.9632	13.5967	0.648870	26.3820
112	1140.7409	1.0809	0.053229	5.3650
113	1142.4255	1.1275	0.055690	4.5082
114	1147.2897	1.0690	0.053252	5.8697
115	1169.4712	4.9127	0.254269	15.4094
116	1172.8559	5.5918	0.291095	10.3809
117	1173.6835	5.3832	0.280628	6.9615

118	1224.7729	7.8390	0.445004	2.3091
119	1229.5544	9.2879	0.531381	0.1847
120	1229.7737	9.5056	0.544029	3.6841
121	1268.7172	1.5660	0.095391	48.5733
122	1274.9974	1.5443	0.095004	19.0636
123	1281.7783	1.5547	0.096661	27.3871
124	1325.2295	10.4845	0.696821	5.4325
125	1331.8185	9.5135	0.638589	7.3561
126	1337.0194	6.7034	0.453484	3.0902
127	1385.4737	3.1928	0.231935	68.9860
128	1387.9138	3.4354	0.250438	27.8143
129	1394.8106	3.5634	0.262355	33.1723
130	1425.2831	3.2552	0.250250	114.4425
131	1430.8842	3.2989	0.255602	130.7611
132	1431.6889	3.8420	0.298019	119.2810
133	1460.3167	2.7599	0.222730	18.0930
134	1462.3810	2.7318	0.221085	15.0647
135	1467.7993	2.5104	0.204674	22.2354
136	1519.1611	7.0300	0.613983	26.3814
137	1523.4036	6.4328	0.564969	31.9579
138	1532.9137	6.4131	0.570287	45.6897
139	1561.1818	1.0904	0.100574	176.6409
140	1573.5792	5.5183	0.517098	52.5446
141	1587.4810	1.1948	0.113948	125.5391
142	1587.5772	3.9079	0.372734	42.6265
143	1595.1689	1.1386	0.109641	150.2046
144	1604.0251	6.3157	0.614946	21.7480
145	1619.1329	6.2082	0.615917	111.1525
146	1619.8770	6.3714	0.632689	78.1724
147	1640.7830	6.0399	0.615348	79.2077
148	3110.8097	1.0881	0.398487	13.6954
149	3113.8178	1.0881	0.399247	8.4716
150	3115.0278	1.0909	0.400593	2.1597
151	3117.8811	1.0885	0.400456	7.8709
152	3122.2200	1.0898	0.402054	4.9748
153	3132.0306	1.0914	0.405170	5.0657
154	3133.7103	1.0954	0.407068	14.9865
155	3135.1621	1.0962	0.407740	17.1118
156	3138.8173	1.0960	0.408646	4.1636
157	3139.7332	1.0963	0.408971	5.7424
158	3140.9510	1.0961	0.409231	4.2502
159	3151.9753	1.0938	0.411240	17.3598
160	3476.5829	1.0580	0.483939	317.3960
161	3528.0385	1.0549	0.496886	264.5080

162	3638.8403	1.0454	0.523836	112.4876
163	3651.9527	1.0704	0.540228	293.9752
164	3701.2983	1.0738	0.556710	254.1016
165	3720.4380	1.0819	0.566696	199.1785

Am1 frequency results

Index	Frequency (cm-1)	Red. mass (u)	F const (Ha/Bohr^2)	Intensity (km/mol)
7	57.7973	1.5120	0.000191	27.7759
8	-57.0993	3.9457	-0.000487	17.8066
9	-52.6525	5.0980	-0.000535	0.8833
10	-40.6973	5.7353	-0.000359	2.8640
11	-37.0563	6.0773	-0.000316	5.4909
12	-23.8791	7.5414	-0.000163	15.7500
13	-18.4464	6.0744	-0.000078	22.6009
14	-11.7724	6.7316	-0.000035	16.8053
15	25.4435	7.0064	0.000172	5.0709
16	22.7591	4.6892	0.000092	28.8656
17	54.8137	6.5977	0.000750	9.3187
18	56.0578	5.8939	0.000701	3.2464
19	68.0235	4.2505	0.000744	12.2756
20	76.8358	5.4206	0.001211	5.3535
21	86.4431	6.4772	0.001832	4.6121
22	88.8151	4.0392	0.001206	4.8037
23	93.4600	3.6492	0.001206	4.2039
24	97.6542	6.1554	0.002221	4.5573
25	100.0009	7.2018	0.002725	2.1419
26	109.5116	5.8319	0.002647	2.3010
27	116.7727	6.3576	0.003281	20.1975
28	123.4250	12.3204	0.007103	71.6261
29	126.7675	10.3259	0.006280	21.8914
30	133.6207	14.5961	0.009862	53.3808
31	144.3925	8.7174	0.006878	18.2611
32	148.2422	5.6753	0.004720	13.8661
33	169.6724	3.8036	0.004144	42.4665
34	176.2660	4.9903	0.005868	8.9322
35	179.8335	4.3171	0.005284	18.8036
36	181.6069	4.2299	0.005279	36.6805
37	188.8750	3.9227	0.005296	20.4503
38	208.7828	1.5869	0.002618	146.0441
39	237.1812	1.7654	0.003758	85.8330
40	254.8131	1.5390	0.003782	346.0914
41	271.5063	5.2147	0.014547	8.6012
42	275.4906	4.6959	0.013487	14.5953
43	277.8749	4.7940	0.014008	14.8657
44	281.6975	4.6679	0.014018	14.6630
45	286.8891	4.7986	0.014946	11.8945
46	320.7468	6.6659	0.025952	29.3373
47	355.4158	1.2127	0.005797	101.1303

48	366.9736	7.9952	0.040747	14.1321
49	368.1225	8.6356	0.044286	9.8101
50	370.1175	8.8346	0.045799	6.8270
51	383.2239	1.6071	0.008932	121.3102
52	393.4473	2.2639	0.013262	40.7959
53	396.4886	2.7199	0.016181	14.4242
54	399.6925	3.7132	0.022449	10.2487
55	438.0226	1.2581	0.009135	232.7627
56	466.5731	6.9127	0.056948	1.7891
57	470.0582	7.2667	0.060762	1.1577
58	470.6494	6.9894	0.058590	14.9541
59	492.6479	3.3644	0.030901	1.0326
60	496.8706	3.2437	0.030305	11.3771
61	498.7821	3.3653	0.031684	6.1889
62	523.2071	1.1420	0.011831	190.2427
63	561.7782	1.1056	0.013205	185.3149
64	629.4214	7.0494	0.105688	19.7009
65	630.0730	7.0059	0.105254	4.7132
66	632.3616	7.0223	0.106268	7.4371
67	710.8660	4.3496	0.083179	37.1415
68	712.7845	3.5599	0.068446	56.1882
69	713.4268	6.2936	0.121225	13.5474
70	713.6984	9.8764	0.190380	5.7413
71	714.1524	6.5415	0.126255	19.7615
72	715.0105	6.9181	0.133846	24.9274
73	715.3936	3.5529	0.068812	60.3200
74	716.0597	4.3366	0.084147	45.7967
75	717.4892	3.4743	0.067684	15.4826
76	728.3430	2.3766	0.047711	13.9983
77	729.1576	2.2122	0.044510	21.4887
78	729.7155	2.6086	0.052565	7.4439
79	779.8853	3.5369	0.081410	9.9191
80	780.3352	3.6414	0.083912	25.0725
81	782.0343	3.4296	0.079375	17.9009
82	865.0578	1.3568	0.038425	0.8263
83	867.5147	1.3527	0.038525	1.1431
84	873.7823	1.3420	0.038776	0.6805
85	941.7805	1.3995	0.046975	2.2169
86	945.6610	1.3868	0.046932	4.2624
87	952.9537	1.3763	0.047299	2.3939
88	973.0780	1.3253	0.047491	0.5426
89	976.0470	1.3239	0.047730	0.2907
90	977.9097	1.3135	0.047534	0.0308
91	1003.2436	6.9601	0.265105	26.0232

92	1004.5885	8.3924	0.320519	8.8093
93	1005.2879	7.3567	0.281356	15.7480
94	1008.3126	9.2995	0.357800	2.9585
95	1016.9443	10.4916	0.410610	13.9381
96	1018.0216	10.0394	0.393744	9.1810
97	1032.4948	10.8753	0.438741	13.3386
98	1034.0246	12.4024	0.501831	28.4746
99	1036.5291	12.0423	0.489627	21.0582
100	1061.4910	2.0172	0.086013	13.7660
101	1063.8432	2.0475	0.087695	6.1048
102	1064.0543	2.1847	0.093606	27.9345
103	1067.9102	8.1904	0.353482	8.7995
104	1070.2085	9.3152	0.403758	2.5697
105	1072.3235	8.7549	0.380973	5.8796
106	1099.1846	1.6017	0.073233	25.0018
107	1101.3319	1.6142	0.074093	8.1996
108	1111.2418	1.7929	0.083783	5.6742
109	1113.9533	12.6836	0.595620	43.3611
110	1122.3676	12.1531	0.579361	46.8641
111	1128.8392	13.8415	0.667481	32.7451
112	1142.2438	1.0821	0.053427	5.1832
113	1146.9266	1.1250	0.056004	4.0740
114	1147.4311	1.0708	0.053351	5.9934
115	1172.5539	4.8728	0.253536	13.4940
116	1174.4366	5.6815	0.296559	13.5190
117	1174.9302	5.7664	0.301247	3.9050
118	1228.5121	8.2725	0.472486	1.5885
119	1229.6189	8.2926	0.474486	4.1003
120	1231.4155	8.8457	0.507614	0.9920
121	1272.8321	1.5359	0.094166	45.4930
122	1278.5733	1.5112	0.093491	15.7036
123	1283.8174	1.5223	0.094952	22.9968
124	1332.4071	9.7999	0.658399	4.3641
125	1340.3864	7.1289	0.484701	3.6483
126	1342.2705	9.6390	0.657210	6.3023
127	1394.2649	3.1921	0.234831	52.7774
128	1396.2113	3.3226	0.245113	82.8514
129	1397.6167	3.3940	0.250887	0.9614
130	1434.0828	3.4390	0.267653	121.6687
131	1440.0436	3.8601	0.302931	8.4607
132	1440.5528	3.8449	0.301949	232.1982
133	1466.5883	2.8384	0.231037	27.3457
134	1466.9821	2.7657	0.225239	9.9224
135	1471.1182	2.5366	0.207752	18.1832

136	1526.3991	6.9143	0.609645	40.6487
137	1536.3654	6.6672	0.595562	38.3596
138	1536.7528	6.4202	0.573780	29.4138
139	1579.1845	1.0979	0.103611	153.1590
140	1587.7618	5.3833	0.513580	37.3570
141	1592.8833	6.2846	0.603441	38.4460
142	1603.6052	1.0837	0.105466	101.8576
143	1604.2034	6.1172	0.595747	31.2655
144	1614.7438	1.1190	0.110417	166.2221
145	1626.1247	5.9536	0.595769	83.3684
146	1628.2049	6.1687	0.618880	63.2928
147	1647.9119	6.0549	0.622250	66.9566
148	3094.1556	1.0898	0.394838	10.6442
149	3100.4694	1.0907	0.396793	1.2782
150	3101.4240	1.0893	0.396506	3.5862
151	3101.8535	1.0887	0.396420	3.8763
152	3114.3717	1.0907	0.400330	0.5388
153	3116.2756	1.0931	0.401725	1.0698
154	3119.4440	1.0949	0.403182	3.1708
155	3121.3546	1.0957	0.403973	0.8960
156	3122.3373	1.0976	0.404953	1.1465
157	3123.8053	1.0987	0.405723	3.7907
158	3127.6139	1.0981	0.406495	10.0096
159	3151.5656	1.0930	0.410830	11.9452
160	3365.3051	1.0603	0.454443	342.9988
161	3389.3560	1.0584	0.460144	319.5227
162	3513.7060	1.0487	0.489957	135.9455
163	3532.1975	1.0691	0.504771	297.2921
164	3573.9300	1.0714	0.517873	256.6427
165	3587.8867	1.0794	0.525840	202.1606

Pu1 frequency results

Index	Frequency (cm-1)	Red. mass (u)	F const (Ha/Bohr^2)	Intensity (km/mol)
7	27.1960	1.4539	0.000041	45.8161
8	-75.0572	4.6150	-0.000984	19.1816
9	-64.2772	4.5575	-0.000713	8.0946
10	-57.6529	7.3517	-0.000925	1.6087
11	-49.8088	5.5378	-0.000520	19.0393
12	-34.1400	4.5398	-0.000200	58.6997
13	-33.3165	5.7567	-0.000242	12.6578
14	-30.9405	5.6530	-0.000205	40.1638
15	13.9709	3.9784	0.000029	19.5314
16	41.7393	5.7879	0.000382	7.5486
17	36.7683	5.1258	0.000262	43.1068
18	50.3768	5.7599	0.000553	8.6803
19	55.9187	5.1468	0.000609	10.1858
20	71.1184	5.2339	0.001002	1.4214
21	76.3570	4.8838	0.001078	2.6193
22	83.6735	5.6438	0.001495	6.0215
23	85.2374	3.1670	0.000871	4.1244
24	88.2961	6.8759	0.002029	3.4237
25	96.5436	8.1764	0.002884	11.4459
26	101.5672	5.2921	0.002066	15.0505
27	108.5232	7.9570	0.003546	42.9480
28	119.8554	10.3115	0.005606	44.6993
29	124.7885	8.9996	0.005304	18.0726
30	128.3734	10.7430	0.006700	15.6433
31	137.1402	7.7129	0.005490	54.1399
32	143.4950	6.4155	0.004999	24.7284
33	151.6118	3.3365	0.002902	58.3163
34	166.4509	3.6468	0.003824	18.7739
35	173.3708	4.7855	0.005443	7.8981
36	176.9514	5.1723	0.006129	17.1053
37	180.5107	5.9056	0.007282	5.3429
38	186.5793	3.9278	0.005174	14.9256
39	248.6664	1.7405	0.004073	39.5489
40	266.9398	5.5188	0.014882	10.1140
41	271.1736	5.1366	0.014294	41.7547
42	276.8362	4.5870	0.013303	44.6819
43	277.1814	4.4413	0.012913	55.0760
44	282.0139	3.8929	0.011717	26.1639
45	299.9299	1.2790	0.004354	296.0816
46	317.8035	7.2480	0.027703	36.6470
47	364.1116	8.9355	0.044831	17.1935
48	364.7924	8.8197	0.044416	7.9615

49	367.0535	8.9364	0.045563	3.2500
50	380.9049	1.4517	0.007971	89.3348
51	386.2904	2.9042	0.016400	33.0805
52	396.3764	2.3752	0.014123	27.7392
53	400.4928	3.6787	0.022329	15.4821
54	414.9576	1.1916	0.007765	100.5093
55	448.9592	1.2285	0.009371	264.5358
56	466.6519	6.8863	0.056750	0.5071
57	468.7879	5.7565	0.047874	6.6380
58	470.1156	6.2001	0.051856	29.0369
59	479.3699	1.2528	0.010895	135.5849
60	493.0772	3.1933	0.029381	4.5286
61	496.4929	3.3011	0.030794	12.6267
62	502.2960	3.2704	0.031226	32.5475
63	548.3780	1.1422	0.012999	167.0669
64	628.3591	7.0587	0.105471	13.2459
65	629.3130	7.0375	0.105474	5.5266
66	631.8755	7.0375	0.106334	6.0855
67	709.8756	6.0559	0.115488	28.5325
68	711.8582	6.0478	0.115978	24.3489
69	712.3034	8.1581	0.156643	9.5521
70	713.1446	6.6625	0.128228	24.6095
71	713.6361	9.2155	0.177608	2.2788
72	714.9958	9.2928	0.179782	3.3558
73	715.4896	4.9939	0.096747	64.1676
74	717.2181	4.7549	0.092562	33.6996
75	718.0429	4.9702	0.096976	17.0494
76	733.0912	1.8513	0.037651	30.8453
77	733.3122	1.9080	0.038828	19.7971
78	733.3853	1.8756	0.038176	39.1995
79	782.2161	3.1936	0.073949	12.2505
80	782.6478	3.3239	0.077049	32.5256
81	784.1628	3.0988	0.072111	20.5640
82	874.5568	1.3382	0.038734	0.5560
83	876.6369	1.3373	0.038891	1.1292
84	882.5270	1.3320	0.039262	0.6938
85	948.0468	1.4099	0.047954	2.6826
86	955.4475	1.3804	0.047689	5.9469
87	960.3191	1.3866	0.048393	2.3295
88	982.9212	1.3268	0.048512	1.0572
89	983.8345	1.3249	0.048530	0.4581
90	989.4476	1.3079	0.048456	0.1645
91	1002.6253	6.8155	0.259280	17.7741
92	1004.5677	7.8461	0.299645	12.6701

93	1005.7326	7.5849	0.290341	12.1632
94	1008.7384	9.5262	0.366832	1.6706
95	1014.4656	10.5970	0.412712	11.8545
96	1021.1311	10.2903	0.406055	12.8712
97	1033.1371	10.9425	0.442001	18.3806
98	1034.0577	11.8410	0.479146	25.6574
99	1039.5671	12.0698	0.493623	21.7337
100	1051.8271	1.9524	0.081742	20.6576
101	1058.5990	10.8342	0.459462	16.4435
102	1064.6797	9.7690	0.419064	8.3002
103	1065.5873	1.9521	0.083882	15.2336
104	1070.8756	2.0932	0.090839	6.4050
105	1080.8985	9.1404	0.404136	1.2157
106	1097.2603	1.6387	0.074662	13.0663
107	1100.0191	1.5810	0.072397	19.4428
108	1111.7399	13.2441	0.619472	27.0881
109	1116.9654	1.6698	0.078839	13.4514
110	1127.7549	13.8164	0.664991	38.9950
111	1131.2221	13.4934	0.653443	24.0484
112	1143.4345	1.0810	0.053485	5.3559
113	1144.9684	1.1484	0.056976	3.9051
114	1149.3322	1.0676	0.053367	5.6923
115	1163.0322	5.7706	0.295388	8.0257
116	1178.6871	6.2030	0.326131	14.0680
117	1186.8251	4.9338	0.262997	10.8228
118	1221.0580	7.9482	0.448472	0.4615
119	1231.6721	7.9519	0.456515	1.4902
120	1242.2352	8.7990	0.513846	2.0709
121	1272.0075	1.5561	0.095279	35.9292
122	1278.2420	1.4893	0.092087	9.3786
123	1286.2250	1.4847	0.092953	17.3029
124	1335.9528	9.7188	0.656424	4.2216
125	1340.3846	8.7588	0.595518	5.8249
126	1347.8918	5.6378	0.387626	1.4875
127	1388.8520	3.1523	0.230110	47.3092
128	1409.6303	2.7512	0.206884	29.3429
129	1411.9734	3.0114	0.227203	24.2951
130	1437.2498	3.4175	0.267160	118.8850
131	1448.6781	2.7499	0.218399	95.7575
132	1458.4052	4.3591	0.350873	87.6344
133	1462.9591	2.8736	0.232748	23.2873
134	1472.2096	3.1788	0.260736	42.8191
135	1474.2294	4.9922	0.410592	75.0504
136	1517.3880	6.5090	0.567151	29.6995

137	1527.3891	7.5035	0.662455	2.8751
138	1535.4217	5.9746	0.533037	43.7295
139	1555.2927	1.0910	0.099870	181.1817
140	1570.9145	5.9424	0.554956	47.8073
141	1587.2887	1.0846	0.103411	114.3196
142	1594.7188	2.6842	0.258331	155.4574
143	1596.2813	1.4446	0.139303	51.7550
144	1614.5274	6.3764	0.629006	46.2697
145	1623.9606	6.6590	0.664582	46.1394
146	1626.1844	6.4143	0.641919	69.6902
147	1661.3730	6.3458	0.662841	64.5203
148	3109.1676	1.0881	0.398065	13.3425
149	3113.1465	1.0908	0.400078	3.1665
150	3113.5500	1.0875	0.398971	5.4419
151	3118.0962	1.0881	0.400359	4.5403
152	3124.5649	1.0909	0.403039	3.8118
153	3129.7337	1.0904	0.404183	2.9973
154	3133.8008	1.0947	0.406862	13.3324
155	3134.2516	1.0957	0.407335	19.2208
156	3134.8876	1.0962	0.407692	8.1823
157	3138.0414	1.0971	0.408845	8.2278
158	3139.6125	1.0962	0.408917	5.9657
159	3148.5645	1.0951	0.410841	21.5106
160	3486.6353	1.0574	0.486444	302.2404
161	3532.6398	1.0546	0.498064	238.1136
162	3632.1359	1.0455	0.521942	109.7720
163	3648.6486	1.0709	0.539541	277.0038
164	3711.0045	1.0741	0.559760	263.7738
165	3713.9397	1.0816	0.564594	191.7466

Section S12: Cartesian coordinates for all structures

La1

La	3.4089	5.2563	11.3498
O	5.3550	4.0151	12.3672
H	6.1134	4.3630	12.1196
H	5.3479	4.1265	13.2259
O	5.3281	6.6535	10.4506
H	5.0506	7.4536	10.2468
H	5.9597	6.7766	11.0587
O	2.6941	2.8852	11.5623
H	2.3965	2.3740	10.9365
H	2.9550	2.3625	12.1920
N	2.0475	7.6285	11.4991
N	-1.3287	4.7428	11.5785
N	4.3844	3.7412	9.2584
N	-1.2390	6.0848	11.7328
N	2.5553	4.8284	13.9365
N	5.4529	8.1487	14.6156
N	2.7673	5.9459	8.8636
N	0.7612	5.1994	11.3705
N	4.4028	6.7717	13.3111
N	-0.1577	4.2086	11.3625
N	5.2654	7.8024	13.3601
N	4.7200	7.3528	15.4221
N	1.9779	6.8946	8.3228
N	1.8632	6.6723	7.0345
N	2.5730	5.5810	6.6884
C	0.7273	7.6125	11.7816
C	4.0120	4.0117	7.9900
C	3.1465	5.4678	14.9716
C	0.0697	6.3222	11.6078
C	4.0857	6.5214	14.5899
C	3.1227	5.1600	7.8332
C	5.1829	2.6778	9.4544
H	5.4410	2.4658	10.3438
C	2.6941	8.7947	11.6415
H	3.6127	8.8332	11.4033
C	1.3349	3.4998	15.5174
H	0.6948	2.8160	15.6783
C	0.0432	8.7247	12.2754
H	-0.8848	8.6758	12.4710
C	1.6612	3.8738	14.2302
H	1.2272	3.4296	13.5110

C	5.6489	1.8741	8.4248
H	6.2175	1.1349	8.6060
C	4.4350	3.2557	6.8996
H	4.1577	3.4783	6.0187
C	2.0871	9.9496	12.1196
H	2.5816	10.7568	12.2019
C	0.7480	9.9024	12.4744
H	0.3197	10.6640	12.8476
C	2.8793	5.1494	16.3004
H	3.3164	5.6101	17.0069
C	1.9634	4.1463	16.5704
H	1.7675	3.9038	17.4679
C	5.2685	2.1717	7.1259
H	5.5740	1.6398	6.4006

Ce1

Ce	3.3766	5.2458	11.3282
O	5.2970	4.0147	12.3463
H	6.0508	4.3507	12.0842
H	5.2996	4.1422	13.1999
O	5.2678	6.6285	10.4302
H	4.9909	7.4256	10.2245
H	5.8913	6.7541	11.0376
O	2.6701	2.9017	11.5289
H	2.3953	2.3971	10.8898
H	2.9631	2.3721	12.1358
N	2.0389	7.6003	11.4681
N	-1.3324	4.7174	11.5499
N	4.3604	3.7401	9.2634
N	-1.2423	6.0572	11.7090
N	2.5227	4.8193	13.8905
N	5.4113	8.1454	14.5641
N	2.7357	5.9306	8.8670
N	0.7574	5.1754	11.3339
N	4.3646	6.7563	13.2641
N	-0.1607	4.1831	11.3262
N	5.2307	7.7896	13.3090
N	4.6746	7.3553	15.3678
N	1.9429	6.8785	8.3270
N	1.8342	6.6606	7.0354
N	2.5492	5.5727	6.6917
C	0.7230	7.5891	11.7551
C	3.9901	4.0040	7.9900
C	3.1078	5.4612	14.9253

C	0.0662	6.2937	11.5801
C	4.0465	6.5179	14.5445
C	3.0984	5.1520	7.8364
C	5.1562	2.6715	9.4601
H	5.4070	2.4554	10.3507
C	2.6789	8.7724	11.5965
H	3.5942	8.8137	11.3469
C	1.2996	3.4922	15.4713
H	0.6605	2.8086	15.6328
C	0.0384	8.6998	12.2438
H	-0.8891	8.6504	12.4421
C	1.6293	3.8599	14.1867
H	1.2009	3.4095	13.4683
C	5.6290	1.8702	8.4329
H	6.1998	1.1332	8.6152
C	4.4163	3.2488	6.9030
H	4.1418	3.4695	6.0207
C	2.0756	9.9291	12.0746
H	2.5699	10.7368	12.1524
C	0.7438	9.8807	12.4346
H	0.3164	10.6433	12.8066
C	2.8350	5.1508	16.2518
H	3.2645	5.6206	16.9568
C	1.9259	4.1460	16.5272
H	1.7320	3.9053	17.4254
C	5.2507	2.1680	7.1357
H	5.5597	1.6362	6.4117

Pr1

Pr	3.3505	5.2319	11.3039
O	5.2601	4.0131	12.3184
H	5.9902	4.3545	12.0498
H	5.2626	4.1415	13.1595
O	5.2262	6.6100	10.4150
H	5.9106	6.5658	10.9637
O	2.6480	2.9044	11.5008
H	2.3684	2.3789	10.8948
H	2.9109	2.3896	12.1246
N	2.0317	7.5841	11.4443
N	-1.3429	4.6999	11.5194
N	4.3435	3.7291	9.2601
N	-1.2527	6.0391	11.6864
N	2.4911	4.8074	13.8477
N	5.3828	8.1284	14.5180

N	2.7200	5.9141	8.8581
N	0.7435	5.1636	11.3017
N	4.3433	6.7366	13.2222
N	-0.1675	4.1714	11.2911
N	5.2059	7.7716	13.2667
N	4.6460	7.3433	15.3286
N	1.9283	6.8660	8.3234
N	1.8246	6.6519	7.0285
N	2.5380	5.5611	6.6831
C	0.7120	7.5745	11.7318
C	3.9745	3.9906	7.9862
C	3.0782	5.4499	14.8843
C	0.0540	6.2813	11.5520
C	4.0178	6.5028	14.5011
C	3.0820	5.1383	7.8290
C	5.1397	2.6641	9.4582
H	5.3924	2.4507	10.3487
C	2.6778	8.7560	11.5723
H	3.5936	8.7957	11.3245
C	1.2676	3.4812	15.4290
H	0.6305	2.7953	15.5896
C	0.0314	8.6797	12.2273
H	-0.8949	8.6279	12.4336
C	1.5982	3.8513	14.1430
H	1.1691	3.4027	13.4241
C	5.6114	1.8614	8.4314
H	6.1806	1.1236	8.6175
C	4.4033	3.2337	6.9002
H	4.1288	3.4531	6.0176
C	2.0737	9.9088	12.0473
H	2.5672	10.7173	12.1205
C	0.7364	9.8639	12.4146
H	0.3111	10.6272	12.7869
C	2.8059	5.1369	16.2135
H	3.2397	5.6009	16.9199
C	1.8900	4.1363	16.4839
H	1.6882	3.8998	17.3817
C	5.2410	2.1523	7.1329
H	5.5540	1.6202	6.4108
H	5.2974	7.0946	9.6681

Nd1

Nd	3.3250	5.2239	11.2748
O	5.2165	4.0158	12.2855

O	5.1895	6.5825	10.3860
O	2.6278	2.9093	11.4686
N	2.0233	7.5641	11.4081
N	-1.3497	4.6845	11.4893
N	4.3210	3.7259	9.2486
N	-1.2584	6.0238	11.6561
N	2.4690	4.8001	13.8011
N	5.3512	8.1230	14.4644
N	2.6906	5.9015	8.8503
N	0.7393	5.1503	11.2678
N	4.3171	6.7232	13.1744
N	-0.1716	4.1588	11.2587
N	5.1788	7.7561	13.2132
N	4.6132	7.3451	15.2735
N	1.9000	6.8529	8.3162
N	1.7996	6.6425	7.0244
N	2.5173	5.5570	6.6748
C	0.7053	7.5577	11.6980
C	3.9535	3.9863	7.9764
C	3.0501	5.4470	14.8365
C	0.0462	6.2653	11.5223
C	3.9888	6.4984	14.4525
C	3.0584	5.1317	7.8179
C	5.1186	2.6631	9.4484
C	2.6630	8.7389	11.5254
C	1.2413	3.4817	15.3827
C	0.0270	8.6681	12.1841
C	1.5752	3.8436	14.0960
C	5.5897	1.8568	8.4219
C	4.3831	3.2321	6.8924
C	2.0647	9.8904	11.9931
C	0.7303	9.8488	12.3675
C	2.7727	5.1390	16.1640
C	1.8599	4.1378	16.4360
C	5.2184	2.1548	7.1221
H	3.4999	8.6724	11.2667
H	1.1718	3.4229	13.3623
H	5.3296	2.4831	10.3356
H	3.1950	5.5822	16.8345
H	5.9291	4.1928	12.0386
H	6.1584	1.1026	8.6177
H	1.6796	3.9185	17.2537
H	5.9060	6.6830	10.7365
H	-0.9079	8.5874	12.4048

H	4.0905	3.4477	6.0467
H	5.5168	1.5876	6.4161
H	5.2367	6.9945	9.7624
H	5.2951	3.8884	13.0939
H	0.6686	2.8088	15.5142
H	2.5282	10.6582	12.0651
H	0.3308	10.6033	12.7560
H	2.8639	2.4230	12.0668
H	2.3335	2.4106	10.8641

Sm1

Sm	3.2899	5.2084	11.2551
O	5.1566	4.0215	12.2652
O	5.1330	6.5457	10.3688
O	2.6032	2.9217	11.4389
N	2.0128	7.5347	11.3801
N	-1.3564	4.6560	11.4589
N	4.2970	3.7110	9.2548
N	-1.2656	5.9940	11.6355
N	2.4302	4.7860	13.7572
N	5.3099	8.1091	14.4143
N	2.6577	5.8719	8.8560
N	0.7309	5.1271	11.2336
N	4.2769	6.7028	13.1304
N	-0.1774	4.1335	11.2219
N	5.1403	7.7360	13.1650
N	4.5691	7.3341	15.2279
N	1.8675	6.8239	8.3217
N	1.7729	6.6187	7.0284
N	2.4962	5.5373	6.6767
C	0.6962	7.5289	11.6738
C	3.9314	3.9676	7.9824
C	3.0053	5.4362	14.7931
C	0.0379	6.2398	11.4984
C	3.9460	6.4839	14.4087
C	3.0332	5.1098	7.8212
C	5.0942	2.6502	9.4593
C	2.6538	8.7062	11.4844
C	1.1996	3.4708	15.3373
C	0.0199	8.6410	12.1611
C	1.5377	3.8310	14.0508
C	5.5702	1.8431	8.4343
C	4.3638	3.2099	6.8965
C	2.0583	9.8649	11.9532

C	0.7234	9.8230	12.3373
C	2.7260	5.1329	16.1218
C	1.8145	4.1282	16.3927
C	5.2034	2.1356	7.1334
H	3.4859	8.6562	11.2237
H	1.1356	3.4250	13.3312
H	5.3008	2.4813	10.3394
H	3.1615	5.5829	16.7959
H	5.8712	4.1620	12.0171
H	6.1372	1.0922	8.6269
H	1.6340	3.9128	17.2174
H	5.8365	6.6468	10.7460
H	-0.9152	8.5661	12.3791
H	4.0532	3.4109	6.0267
H	5.4839	1.5552	6.4317
H	5.1926	6.9508	9.7592
H	5.2385	3.8757	13.0701
H	0.6694	2.7658	15.4669
H	2.5362	10.6480	12.0171
H	0.3422	10.5791	12.7411
H	2.8432	2.4689	12.0188
H	2.3083	2.4389	10.8187

Eu1

Eu	3.2889	5.2055	11.2425
O	5.1461	4.0260	12.2502
O	5.1245	6.5271	10.3541
O	2.6053	2.9269	11.4239
N	2.0233	7.5255	11.3656
N	-1.3470	4.6497	11.4452
N	4.2957	3.7105	9.2551
N	-1.2558	5.9870	11.6233
N	2.4328	4.7863	13.7319
N	5.3107	8.1103	14.3836
N	2.6504	5.8642	8.8577
N	0.7414	5.1205	11.2210
N	4.2765	6.6994	13.1039
N	-0.1664	4.1278	11.2068
N	5.1421	7.7312	13.1369
N	4.5682	7.3401	15.1998
N	1.8567	6.8145	8.3257
N	1.7614	6.6132	7.0316
N	2.4883	5.5337	6.6775
C	0.7083	7.5228	11.6606

C	3.9278	3.9664	7.9817
C	3.0049	5.4391	14.7688
C	0.0461	6.2330	11.4861
C	3.9468	6.4872	14.3819
C	3.0272	5.1033	7.8206
C	5.0934	2.6515	9.4608
C	2.6639	8.6997	11.4633
C	1.2017	3.4719	15.3147
C	0.0303	8.6354	12.1455
C	1.5389	3.8314	14.0259
C	5.5689	1.8412	8.4338
C	4.3612	3.2066	6.8989
C	2.0694	9.8599	11.9260
C	0.7356	9.8180	12.3145
C	2.7247	5.1385	16.0981
C	1.8123	4.1327	16.3694
C	5.1986	2.1325	7.1349
H	3.5228	8.6511	11.1977
H	1.1341	3.4198	13.2957
H	5.2823	2.4902	10.3510
H	3.1444	5.6077	16.7817
H	5.8692	4.1850	11.9818
H	6.1202	1.0851	8.6178
H	1.6195	3.9111	17.2060
H	5.8344	6.6187	10.7092
H	-0.9010	8.5751	12.3796
H	4.0580	3.4233	6.0148
H	5.4929	1.5606	6.4324
H	5.1599	6.9774	9.7485
H	5.2261	3.9040	13.0514
H	0.6426	2.7641	15.4299
H	2.5407	10.6323	11.9818
H	0.3533	10.5686	12.6932
H	2.8739	2.4389	11.9851
H	2.3209	2.4460	10.8412

Gd1

Gd	3.2812	5.1997	11.2329
O	5.1324	4.0271	12.2350
O	5.1063	6.5077	10.3384
O	2.6008	2.9296	11.4129
N	2.0246	7.5138	11.3555
N	-1.3471	4.6427	11.4325
N	4.2875	3.7062	9.2539

N	-1.2547	5.9800	11.6115
N	2.4287	4.7871	13.7125
N	5.3091	8.1063	14.3541
N	2.6419	5.8537	8.8585
N	0.7423	5.1151	11.2113
N	4.2734	6.6925	13.0794
N	-0.1660	4.1206	11.1953
N	5.1391	7.7225	13.1083
N	4.5650	7.3405	15.1728
N	1.8511	6.8070	8.3297
N	1.7582	6.6110	7.0343
N	2.4826	5.5303	6.6777
C	0.7099	7.5133	11.6489
C	3.9202	3.9603	7.9802
C	2.9998	5.4402	14.7482
C	0.0484	6.2265	11.4755
C	3.9430	6.4843	14.3581
C	3.0196	5.0970	7.8201
C	5.0850	2.6486	9.4598
C	2.6662	8.6884	11.4462
C	1.1923	3.4795	15.2946
C	0.0338	8.6304	12.1302
C	1.5337	3.8329	14.0069
C	5.5625	1.8383	8.4341
C	4.3549	3.2017	6.8961
C	2.0713	9.8509	11.9042
C	0.7397	9.8115	12.2927
C	2.7171	5.1433	16.0765
C	1.8027	4.1407	16.3496
C	5.1958	2.1286	7.1346
H	3.5276	8.6373	11.1765
H	1.1210	3.4281	13.2863
H	5.2768	2.4898	10.3277
H	3.1112	5.6104	16.7640
H	5.8952	4.1844	11.9776
H	6.1251	1.0956	8.6366
H	1.6336	3.9352	17.1843
H	5.8426	6.6140	10.7068
H	-0.9377	8.5472	12.3649
H	4.0419	3.4051	6.0176
H	5.5022	1.5691	6.4346
H	5.1386	6.9339	9.7146
H	5.2139	3.8857	13.0621
H	0.6118	2.7725	15.4174

H	2.5280	10.6482	11.9627
H	0.3424	10.5757	12.6764
H	2.8645	2.4332	11.9974
H	2.3061	2.4367	10.7859

Tb1

Tb	3.2474	5.1913	11.2290
O	5.0851	4.0215	12.2327
H	5.8056	4.3600	11.9630
H	5.0869	4.1520	13.0664
O	5.0632	6.4900	10.3387
H	4.7940	7.2788	10.1848
H	5.6433	6.5855	10.9390
O	2.5731	2.9396	11.4015
H	2.2994	2.4419	10.7793
H	2.8575	2.4006	11.9852
N	2.0074	7.5003	11.3437
N	-1.3670	4.6331	11.4253
N	4.2625	3.6987	9.2612
N	-1.2751	5.9689	11.6083
N	2.3933	4.7733	13.6964
N	5.2674	8.0989	14.3390
N	2.6123	5.8402	8.8666
N	0.7236	5.1044	11.2020
N	4.2330	6.6812	13.0662
N	-0.1850	4.1110	11.1834
N	5.0999	7.7126	13.0937
N	4.5224	7.3356	15.1586
N	1.8186	6.7921	8.3361
N	1.7303	6.5979	7.0417
N	2.4591	5.5216	6.6837
C	0.6930	7.5016	11.6455
C	3.8979	3.9524	7.9868
C	2.9599	5.4316	14.7330
C	0.0295	6.2149	11.4711
C	3.9010	6.4764	14.3448
C	2.9952	5.0875	7.8262
C	5.0623	2.6407	9.4686
H	5.3144	2.4328	10.3606
C	2.6499	8.6735	11.4258
H	3.5591	8.7089	11.1530
C	1.1520	3.4681	15.2814
H	0.5118	2.7852	15.4420
C	0.0211	8.6175	12.1288

H	-0.9024	8.5718	12.3466
C	1.4988	3.8196	13.9915
H	1.0798	3.3601	13.2734
C	5.5400	1.8339	8.4417
H	6.1075	1.0964	8.6319
C	4.3335	3.1946	6.9030
H	4.0586	3.4095	6.0195
C	2.0572	9.8385	11.8883
H	2.5518	10.6484	11.9320
C	0.7291	9.8004	12.2861
H	0.3123	10.5687	12.6585
C	2.6737	5.1367	16.0634
H	3.0970	5.6138	16.7677
C	1.7593	4.1324	16.3388
H	1.5504	3.9024	17.2366
C	5.1752	2.1215	7.1399
H	5.4969	1.5919	6.4199

Dy1

Dy	3.2390	5.1826	11.2170
O	5.0680	4.0218	12.2139
H	5.8061	4.3524	11.9517
H	5.0679	4.1548	13.0535
O	5.0428	6.4616	10.3235
H	4.7501	7.2115	10.0367
H	5.6020	6.6690	10.9433
O	2.5653	2.9429	11.3870
H	2.2775	2.4292	10.7757
H	2.8849	2.4263	11.9787
N	2.0151	7.4879	11.3278
N	-1.3613	4.6271	11.4156
N	4.2478	3.6956	9.2568
N	-1.2674	5.9623	11.5960
N	2.3930	4.7763	13.6735
N	5.2659	8.0978	14.2975
N	2.5926	5.8277	8.8696
N	0.7313	5.0958	11.1952
N	4.2313	6.6730	13.0350
N	-0.1799	4.1048	11.1735
N	5.0989	7.7024	13.0555
N	4.5214	7.3412	15.1223
N	1.8001	6.7800	8.3415
N	1.7100	6.5920	7.0483
N	2.4398	5.5198	6.6866

C	0.7026	7.4928	11.6302
C	3.8807	3.9485	7.9841
C	2.9566	5.4363	14.7082
C	0.0347	6.2068	11.4614
C	3.9004	6.4766	14.3154
C	2.9763	5.0799	7.8253
C	5.0481	2.6377	9.4623
H	5.2996	2.4291	10.3544
C	2.6575	8.6595	11.3981
H	3.5664	8.6920	11.1235
C	1.1452	3.4827	15.2643
H	0.5031	2.8025	15.4286
C	0.0323	8.6134	12.1101
H	-0.8904	8.5690	12.3318
C	1.4932	3.8250	13.9726
H	1.0732	3.3626	13.2567
C	5.5268	1.8330	8.4373
H	6.0969	1.0974	8.6271
C	4.3160	3.1942	6.8999
H	4.0410	3.4104	6.0167
C	2.0650	9.8290	11.8512
H	2.5589	10.6398	11.8828
C	0.7397	9.7947	12.2576
H	0.3253	10.5649	12.6290
C	2.6677	5.1491	16.0385
H	3.0904	5.6287	16.7413
C	1.7514	4.1499	16.3161
H	1.5415	3.9249	17.2148
C	5.1598	2.1203	7.1357
H	5.4805	1.5905	6.4153

Ho1

Ho	3.1518	5.1976	11.2566
O	4.9449	4.0210	12.2551
H	5.6811	4.3094	11.9410
H	4.9792	4.2087	13.0837
O	4.9316	6.4409	10.3427
H	4.9068	6.9663	9.6503
H	5.7062	6.5974	10.7060
O	2.4795	2.9568	11.4336
H	2.1561	2.4719	10.8162
H	2.7093	2.4080	12.0391
N	1.9309	7.5037	11.3629
N	-1.4391	4.6446	11.4764

N	4.1327	3.7118	9.2881
N	-1.3409	5.9763	11.6409
N	2.3192	4.8049	13.7042
N	5.1538	8.1713	14.2867
N	2.4447	5.8232	8.9409
N	0.6561	5.1111	11.2608
N	4.1474	6.7026	13.0444
N	-0.2630	4.1177	11.2460
N	5.0100	7.7387	13.0461
N	4.3983	7.4325	15.1209
N	1.6362	6.7916	8.4358
N	1.5622	6.6332	7.1343
N	2.3149	5.5935	6.7509
C	0.6265	7.5108	11.6672
C	3.7598	3.9949	8.0211
C	2.8569	5.5081	14.7325
C	-0.0437	6.2166	11.5175
C	3.7919	6.5388	14.3278
C	2.8588	5.1182	7.8747
C	4.9258	2.6472	9.4789
H	5.1778	2.4242	10.3677
C	2.5553	8.6911	11.3941
H	3.4621	8.7276	11.1123
C	1.0229	3.5694	15.3101
H	0.3662	2.9020	15.4731
C	-0.0526	8.6448	12.1230
H	-0.9729	8.6045	12.3565
C	1.4215	3.8702	14.0250
H	1.0289	3.3759	13.3145
C	5.3947	1.8604	8.4407
H	5.9563	1.1144	8.6190
C	4.2107	3.2454	6.9286
H	3.9498	3.4766	6.0444
C	1.9659	9.8518	11.8071
H	2.4564	10.6652	11.8059
C	0.6582	9.8323	12.2250
H	0.2497	10.6147	12.5751
C	2.5253	5.2553	16.0669
H	2.9282	5.7587	16.7642
C	1.6067	4.2690	16.3598
H	1.3788	4.0714	17.2600
C	5.0363	2.1683	7.1524
H	5.3550	1.6455	6.4253

Pu1

Pu	3.2916	5.2211	11.2819
O	5.1916	3.9902	12.2984
O	5.1569	6.5943	10.3890
O	2.5986	2.9030	11.4780
N	1.9906	7.5561	11.4283
N	-1.3851	4.6725	11.4913
N	4.2929	3.7267	9.2603
N	-1.2830	6.0098	11.6620
N	2.4344	4.7983	13.8117
N	5.3228	8.1158	14.4631
N	2.6615	5.9071	8.8642
N	0.7077	5.1401	11.2741
N	4.2933	6.7112	13.1786
N	-0.2069	4.1429	11.2592
N	5.1555	7.7456	13.2183
N	4.5771	7.3435	15.2703
N	1.8822	6.8635	8.3255
N	1.7916	6.6598	7.0260
N	2.5096	5.5758	6.6829
C	0.6708	7.5454	11.7067
C	3.9328	3.9959	7.9857
C	3.0150	5.4483	14.8410
C	0.0111	6.2471	11.5261
C	3.9635	6.4969	14.4515
C	3.0453	5.1472	7.8249
C	5.0799	2.6533	9.4559
C	-0.0135	8.6542	12.1808
C	1.5371	3.8471	14.1051
C	5.5645	1.8616	8.4216
C	2.0240	9.8835	12.0051
C	0.6963	9.8374	12.3763
C	2.7381	5.1383	16.1653
C	1.8197	4.1464	16.4438
C	5.1943	2.1644	7.1321
H	5.5049	1.6354	6.4068
H	1.1210	3.4008	13.3261
H	5.2707	2.4797	10.2929
H	3.1302	5.5971	16.8731
H	5.9979	4.1447	12.0167
H	6.0846	1.1336	8.5857
H	1.6110	3.9498	17.2543
H	5.9507	6.6421	10.8233
H	-0.9372	8.5905	12.3647

H	4.0906	3.4716	6.0498
H	5.2600	6.9964	9.7791
H	5.2484	3.8967	13.1438
H	2.4752	10.6274	12.1161
H	0.2838	10.6451	12.8123
H	2.8528	2.5151	12.0332
H	2.3094	2.4620	10.8730
C	4.3648	3.2431	6.9034
C	2.6319	8.7251	11.5426
H	3.5480	8.7649	11.2964
C	1.1921	3.4893	15.3714
H	0.5435	2.8128	15.5272

Am1

Am	3.3185	5.2205	11.2646
O	5.2127	4.0035	12.2754
O	5.1939	6.5708	10.3589
H	4.9070	7.3693	10.1455
H	5.7823	6.7141	10.9895
O	2.6304	2.9000	11.4604
H	2.3328	2.3394	10.8660
H	2.9708	2.3977	12.0834
N	2.0282	7.5518	11.4053
N	-1.3430	4.6723	11.4730
N	4.3128	3.7240	9.2546
N	-1.2461	6.0098	11.6441
N	2.4713	4.7941	13.7763
N	5.3597	8.1147	14.4275
N	2.6842	5.8965	8.8588
N	0.7486	5.1385	11.2528
N	4.3254	6.7055	13.1455
N	-0.1625	4.1457	11.2402
N	5.1866	7.7405	13.1796
N	4.6200	7.3395	15.2407
N	1.8997	6.8519	8.3259
N	1.8006	6.6469	7.0324
N	2.5207	5.5678	6.6809
C	0.7128	7.5440	11.6875
C	3.9482	3.9915	7.9811
C	3.0534	5.4423	14.8109
C	0.0571	6.2560	11.5112
C	3.9929	6.4865	14.4222
C	3.0580	5.1352	7.8241
C	5.1065	2.6582	9.4562

C	2.6660	8.7243	11.5186
H	3.5814	8.7626	11.2664
C	1.2397	3.4863	15.3540
C	0.0304	8.6569	12.1692
C	1.5754	3.8424	14.0737
C	5.5812	1.8563	8.4255
C	4.3830	3.2362	6.8971
C	2.0684	9.8827	11.9820
C	0.7288	9.8377	12.3500
C	2.7796	5.1378	16.1378
C	1.8638	4.1393	16.4137
C	5.2153	2.1592	7.1273
H	1.1746	3.4057	13.3486
H	5.3049	2.4835	10.3418
H	3.2083	5.6113	16.8533
H	5.9109	4.1598	12.0569
H	6.1367	1.1471	8.5986
H	1.6324	3.9297	17.2667
H	-0.9563	8.5498	12.3877
H	4.0696	3.3810	6.0367
H	5.5012	1.6462	6.4171
H	5.2745	3.9474	13.1320
H	0.6639	2.8145	15.4971
H	2.5391	10.6562	12.0454
H	0.3324	10.6031	12.7020

Cm1

Cm	3.3166	5.2216	11.2688
O	5.2090	4.0078	12.2779
O	5.1869	6.5538	10.3548
H	4.8899	7.3374	10.0950
H	5.7522	6.7397	10.9960
O	2.6301	2.9058	11.4662
H	2.3584	2.3832	10.8258
H	2.9447	2.3644	12.0700
N	2.0319	7.5483	11.4080
N	-1.3447	4.6671	11.4730
N	4.3148	3.7263	9.2638
N	-1.2552	6.0026	11.6453
N	2.4695	4.8055	13.7739
N	5.3645	8.1215	14.4194
N	2.6753	5.8911	8.8721
N	0.7485	5.1344	11.2590
N	4.3240	6.7095	13.1398

N	-0.1646	4.1437	11.2435
N	5.1963	7.7424	13.1710
N	4.6198	7.3533	15.2355
N	1.8871	6.8472	8.3375
N	1.7892	6.6427	7.0443
N	2.5138	5.5663	6.6917
C	0.7134	7.5437	11.6885
C	3.9478	3.9880	7.9891
C	3.0529	5.4522	14.8064
C	0.0569	6.2522	11.5147
C	3.9952	6.4943	14.4171
C	3.0536	5.1303	7.8307
C	5.1095	2.6592	9.4680
C	2.6706	8.7227	11.5162
H	3.5866	8.7586	11.2677
C	1.2273	3.4985	15.3457
C	0.0288	8.6563	12.1681
C	1.5741	3.8531	14.0691
C	5.5865	1.8568	8.4334
C	4.3851	3.2309	6.9055
C	2.0714	9.8858	11.9690
C	0.7354	9.8391	12.3411
C	2.7729	5.1532	16.1386
C	1.8596	4.1504	16.4109
C	5.2178	2.1576	7.1372
H	1.1625	3.4277	13.3963
H	5.2761	2.4800	10.3532
H	3.1558	5.6153	16.8198
H	5.8958	4.2336	12.0401
H	6.1143	1.1160	8.6332
H	1.6359	3.9502	17.2167
H	-0.9406	8.5559	12.3709
H	4.0865	3.4188	6.1028
H	5.5536	1.5943	6.3343
H	5.2801	4.0034	13.1482
H	0.7286	2.7988	15.5132
H	2.5529	10.6993	12.0567
H	0.3650	10.6107	12.7513

Section S13: Table of N-donors' ligands for practical An/Ln separation

Ligands	Advantages	Disadvantages
2,6-Di(5,6-dialkyl-l,2,4-triazin-3-yl)pyridines (BTP)[19,20]	Good at An/Ln separation	decomposed by 1-brornohexanoic acid
2,6-bis(5,6-iso-propyl-1,2,4-triazin-3-yl)-pyridine (*i*Pr-BTP)[21]	Good at An/Ln separation	radiolysis of the solvent destructed the *i*Pr-BTP
2,6-bis(5,5,8,8-tetramethyl-5,6,7,8-tetrahydrobenzo[e][1,2,4]triazin-3-yl)pyridine ($CyMe_4$-BTP)[22]	Good at An/Ln separation	Too efficiently, resulting in problems during stripping
2,6-bis(5,6-di(sulfophenyl)-1,2,4-triazin-3-yl)pyridine (SO_3-Ph-BTP)[23]	innovative-SANEX processes for heterogenous recycling of the actinides from Np to Cm	contains sulfur and is thus incompatible with the CHON principle
6,60-bis(1,2,4-triazin-3-yl)-2,20-bipyridine (BTBP)[24,25]	The increased number of nitrogen donor atoms significantly enhances the stripping ability of An(III)[25]	The increased number of nitrogen donor atoms significantly enhances the stripping ability of An(III), leading to slow extraction kinetics and require the addition ligands as phase transfer agents to shorten the extraction equilibrium time.[25]
2,2′:6′,2″-terpyridine and its derivatives	Good at An/Ln separation	high basicity
2,6-Di(5-alkyl-1,2,4-triazol-3-yl)pyridines[19]	Effectively extract and separate An(lll) and Ln(lIl)	Can not extract the elements as nitrates.
2,6-bis[1-(propan-1-ol)-1,2,3-triazol-4-yl]pyridine (PyTri-Diol)[26]	highly selective water-soluble stripping agent for trivalent actinides in the innovative-SANEX process	More stages needed
Phenanthroline-derived triazine (BTPhen)[25]	Stable under highly acidic conditions and strong extraction capability	Multi-step synthesis, limited stripping efficiency[27]

Section S14: Reference

(1) Huffman, Z. K.; Sperling, J. M.; Windorff, C. J.; Long, B. N.; Cordova, L.; Ramanantoanina, H.; Celis-Barros, C.; Albrecht-Schönzart, T. E. Synthesis and Characterization of a Bimetallic Americium(III) Pyrithionate Coordination Complex. *Chem. Commun.* **2022**, *58* (84), 11791–11794. https://doi.org/10.1039/D2CC03352F.

(2) *CrysAlisPro, Version 1.171.41.115a*; Rigaku Oxford Diffraction: Oxford, UK, 2021.

(3) *APEX4, Version 2021.4-0*; Bruker AXS Inc.: Madison, Wisconsin, USA, 2021.

(4) Sheldrick, G. M. SHELXT – Integrated Space-Group and Crystal-Structure Determination. *Acta Crystallogr., Sect. A: Found. Adv.* **2015**, *71* (1), 3–8. https://doi.org/10.1107/S2053273314026370.

(5) Sheldrick, G. M. Crystal Structure Refinement with *SHELXL*. *Acta Crystallogr., Sect. C: Struct. Chem.* **2015**, *71* (1), 3–8. https://doi.org/10.1107/S2053229614024218.

(6) Dolomanov, O. V.; Bourhis, L. J.; Gildea, R. J.; Howard, J. A. K.; Puschmann, H. *OLEX2* : A Complete Structure Solution, Refinement and Analysis Program. *J. Appl. Crystallogr.* **2009**, *42* (2), 339–341. https://doi.org/10.1107/S0021889808042726.

(7) Macrae, C. F.; Sovago, I.; Cottrell, S. J.; Galek, P. T. A.; McCabe, P.; Pidcock, E.; Platings, M.; Shields, G. P.; Stevens, J. S.; Towler, M.; Wood, P. A. *Mercury 4.0* : From Visualization to Analysis, Design and Prediction. *J. Appl. Crystallogr.* **2020**, *53* (1), 226–235. https://doi.org/10.1107/S1600576719014092.

(8) te Velde, G.; Bickelhaupt, F. M.; Baerends, E. J.; Fonseca Guerra, C.; van Gisbergen, S. J. A.; Snijders, J. G.; Ziegler, T. Chemistry with ADF. *J. Comput. Chem.* **2001**, *22* (9), 931–967. https://doi.org/10.1002/jcc.1056.

(9) Rodríguez, J. I.; Bader, R. F. W.; Ayers, P. W.; Michel, C.; Götz, A. W.; Bo, C. A High Performance Grid-Based Algorithm for Computing QTAIM Properties. *Chem. Phys. Lett.* **2009**, *472* (1), 149–152. https://doi.org/10.1016/j.cplett.2009.02.081.

(10) Adamo, C.; Barone, V. Toward Reliable Density Functional Methods without Adjustable Parameters: The PBE0 Model. *J. Chem. Phys* **1999**, *110* (13), 6158–6170. https://doi.org/10.1063/1.478522.

(11) Van Lenthe, E.; Baerends, E. J. Optimized Slater-type Basis Sets for the Elements 1–118. *J. Comput. Chem.* **2003**, *24* (9), 1142–1156. https://doi.org/10.1002/jcc.10255.

(12) Van Lenthe, E.; Baerends, E. J.; Snijders, J. G. Relativistic Total Energy Using Regular Approximations. *J. Comput. Chem.* **1994**, *101* (11), 9783–9792. https://doi.org/10.1063/1.467943.

(13) Blanco, M. A.; Martín Pendás, A.; Francisco, E. Interacting Quantum Atoms: A Correlated Energy Decomposition Scheme Based on the Quantum Theory of Atoms in Molecules. *J. Chem. Theory Comput.* **2005**, *1* (6), 1096–1109. https://doi.org/10.1021/ct0501093.

(14) Neese, F.; Wennmohs, F.; Becker, U.; Riplinger, C. The ORCA Quantum Chemistry Program Package. *J. Chem. Phys.* **2020**, *152* (22), 224108. https://doi.org/10.1063/5.0004608.

(15) Casanova, D.; Llunell, M.; Alemany, P.; Alvarez, S. The Rich Stereochemistry of Eight-Vertex Polyhedra: A Continuous Shape Measures Study. *Chem. Eur. J.* **2005**, *11* (5), 1479–1494. https://doi.org/10.1002/chem.200400799.

(16) Bader, R. F. W. *Atoms in Molecules: A Quantum Theory*; International Series of Monographs on Chemistry; Oxford University Press: Oxford, New York, 1994.

(17) Glendening, E. D.; Landis, C. R.; Weinhold, F. NBO 7.0: New Vistas in Localized and Delocalized Chemical Bonding Theory. *Journal of Computational Chemistry* **2019**, *40* (25), 2234–2241. https://doi.org/10.1002/jcc.25873.

(18) Lu, T. A Comprehensive Electron Wavefunction Analysis Toolbox for Chemists, Multiwfn. *J. Chem. Phys.* **2024**, *161* (8), 082503. https://doi.org/10.1063/5.0216272.

(19) Kolarik, Z.; Müllich, U.; Gassner, F. SELECTIVE EXTRACTION OF Am(III) OVER Eu(III) BY 2,6-DITRIAZOLYL- AND 2,6-DITRIAZINYLPYRIDINES1. *Solvent Extr. Ion Exch.* **1999**, *17* (1), 23–32. https://doi.org/10.1080/07360299908934598.

(20) Kolarik (ret.), Z.; Mullich, U.; Gassner, F. EXTRACTION OF Am(Lll) AND Eu(Lll) NITRATES BY 2-6-DI-(5,6-DIPROPYL-1,2,4-TRIAZIN-3-YL)PYRIDINES1. *Solvent Extr. Ion Exch.* **1999**, *17* (5), 1155–1170. https://doi.org/10.1080/07366299908934641.

(21) Madic, C.; Boullis, B.; Baron, P.; Testard, F.; Hudson, M. J.; Liljenzin, J.-O.; Christiansen, B.; Ferrando, M.; Facchini, A.; Geist, A.; Modolo, G.; Espartero, A. G.; De Mendoza, J. Futuristic Back-End of the Nuclear Fuel Cycle with the Partitioning of Minor Actinides. *J. Alloy. Compd.* **2007**, *444–445*, 23–27. https://doi.org/10.1016/j.jallcom.2007.05.051.

(22) Colombo Dugoni, G.; Mossini, E.; Macerata, E.; Sacchetti, A.; Mele, A.; Mariani, M. Deep Eutectic Solvents: Promising Co-Solvents to Improve the Extraction Kinetics of $CyMe_4$ -BTBP. *ACS Omega* **2021**, *6* (5), 3602–3611. https://doi.org/10.1021/acsomega.0c05109.

(23) Carrott, M.; Maher, C.; Mason, C.; Sarsfield, M.; Taylor, R. “TRU-SANEX”: A Variation on the EURO-GANEX and *i* -SANEX Processes for Heterogeneous Recycling of Actinides Np-Cm. *Sep. Sci. Technol.* **2016**, *51* (13), 2198–2213. https://doi.org/10.1080/01496395.2016.1202979.

(24) Retegan, T.; Ekberg, C.; Dubois, I.; Fermvik, A.; Skarnemark, G.; Wass, T. J. Extraction of Actinides with Different 6,6′-Bis(5,6-Dialkyl-[1,2,4]-Triazin-3-yl)-[2,2′]-Bipyridines (BTBPs). *Solvent Extr. Ion Exch.* **2007**, *25* (4), 417–431. https://doi.org/10.1080/07366290701416000.

(25) Yang, X.; Xu, L.; Fang, D.; Zhang, A.; Xiao, C. Progress in Phenanthroline-Derived Extractants for Trivalent Actinides and Lanthanides Separation: Where to Next? *Chem. Commun.* **2024**, *60* (81), 11415–11433. https://doi.org/10.1039/D4CC03810J.

(26) Wilden, A.; Schneider, D.; Paparigas, Z.; Henkes, M.; Kreft, F.; Geist, A.; Mossini, E.; Macerata, E.; Mariani, M.; Gullo, M. C.; Casnati, A.; Modolo, G. Selective Actinide(III) Separation Using 2,6-Bis[1-(Propan-1-Ol)-1,2,3-Triazol-4-Yl]Pyridine (PyTri-Diol) in the Innovative-SANEX Process: Laboratory Scale Counter Current Centrifugal Contactor Demonstration. *Radiochim. Acta* **2022**, *110* (6–9), 515–525. https://doi.org/10.1515/ract-2022-0014.
(27) Geist, A.; Panak, P. J. Recent Progress in Trivalent Actinide and Lanthanide Solvent Extraction and Coordination Chemistry with Triazinylpyridine N Donor Ligands. *Solvent Extr. Ion Exch.* **2021**, *39* (2), 128–151. https://doi.org/10.1080/07366299.2020.1831235.
